# Epidemiology and factors associated with osteoporosis, falls and fractures in patients with chronic inflammatory rheumatic disease: a scoping review

**DOI:** 10.1136/bmjopen-2024-096226

**Published:** 2025-07-28

**Authors:** Catherine Cho, Grace Bak, Bethan Richards, Catherine Sherrington

**Affiliations:** 1 Concord Repatriation General Hospital, Concord, New South Wales, Australia; 2 The University of Sydney, Sydney, New South Wales, Australia; 3 Institute for Musculoskeletal Health, University of Sydney School of Public Health, Sydney, New South Wales, Australia; 4 Rheumatology Department, Institute of Rheumatology & Orthopaedics, Sydney, New South Wales, Australia; 5 Institute for Musculoskeletal Health, University of Sydney, Sydney, New South Wales, Australia

**Keywords:** Rheumatology, Fractures, Bone, EPIDEMIOLOGY, Calcium & bone

## Abstract

**Abstract:**

**Objectives:**

To describe the available literature on the epidemiology and factors associated with osteoporosis, falls and fractures in four chronic inflammatory rheumatic diseases (CIRD): rheumatoid arthritis (RA); psoriatic arthritis (PsA); ankylosing spondylitis (AS); and systemic lupus erythematosus (SLE).

**Design:**

Scoping review, using the Joanna Briggs Institute framework.

**Data sources:**

MEDLINE, Embase and CINAHL from January 2000 to December 2023.

**Eligibility criteria:**

Observational studies reporting on the epidemiology and/or associated factors for osteoporosis, falls and fractures in RA, PsA, AS or SLE.

**Data extraction and synthesis:**

Two independent reviewers used a standard data extraction form including report methods, definitions, outcomes and associated factors. Results are summarised with descriptive statistics.

**Results:**

288 studies met inclusion criteria, with 170 studies on RA, 19 on PsA, 49 on AS and 60 on SLE. Most studies were cross-sectional, with Europe and Asia having the greatest output. Most papers reported on osteoporosis and fractures as outcomes, with only 27 reporting falls, of which 24 were in RA. Participants’ demographics and disease-related parameters were the most frequently explored potential associated factors.

**Conclusions:**

RA was the most well-studied CIRD with regard to the epidemiology and associated factors for osteoporosis, fractures and falls. Cross-sectional was the most common study design, with a higher proportion of cohort studies in RA. There is a paucity of studies assessing falls in CIRDs other than RA. Future observational research should be conducted with large prospective CIRD cohorts, with falls as an outcome and associated factor for fractures. This may enable better understanding of the risk and consequences of osteoporosis, fractures and falls, which may improve preventive care.

**STRENGTHS AND LIMITATIONS OF THIS STUDY**

- Extensive literature review of studies available conducted on the epidemiology and factors associated with osteoporosis, falls and fractures in chronic inflammatory rheumatic diseases (CIRD).
- Identification of a paucity of falls research in CIRD, compared with available literature in osteoporosis and osteoporotic fractures, highlighting the need for further research.
- Included publications from developed and/or resource-rich countries in the English language only is a limitation of the study.

## Background

 Osteoporosis, falls and related fractures are increasing due to the global ageing population, with significant physical and psychological burden to the individual with increased risk of mortality and economic cost to the community.^1^ Chronic inflammatory rheumatic diseases (CIRD), particularly rheumatoid arthritis (RA) and systemic lupus erythematosus (SLE), are associated with higher prevalence of osteoporosis, falls and fractures due to an interplay of complex mechanisms, including disease pathophysiology, treatments, joint deformities and impact on physical activity. ^2^There is an estimated prevalence of osteoporosis of 30% in RA and a twofold increased risk in SLE compared with the general population.^3^,^5^ There is less certainty about the risk of osteoporosis, falls and fractures in spondyloarthropathies, including psoriatic arthritis (PsA) and ankylosing spondylitis (AS), with widely variable results in literature due to challenges with interpreting bone density data, the younger patient demographic and bias in existing studies.^6 7^

With the advent of efficacious disease-modifying agents leading to improved function and life expectancy, there has been an increased focus on addressing CIRD-associated comorbidities, including osteoporosis, falls and related fractures. The European League Against Rheumatism (EULAR) has proposed specific recommendations for the detection and management of relevant comorbidities in routine clinical practice.^8^ The Fracture Risk Assessment Tool (FRAX) is a widely used method of fracture risk assessment inclusive of RA as a risk factor, with its validity studied in several large RA cohorts.^9 10^ However, the validity of population-based fracture risk calculators is not validated in non-RA CIRDs and may underestimate fracture risk, carrying potential implications of under-diagnosis and treatment.^11^

The purpose of this scoping review is to provide a comprehensive overview of the current literature available on the epidemiology and factors associated with osteoporosis, falls and fractures in RA, PsA, AS and SLE; to explore the methodologies used; and to identify gaps in current literature, topics for further research and areas for improvement in the design of future observational studies. This may lead to improved risk stratification of osteoporotic fractures in CIRD patients which may enable earlier diagnosis and intervention.

## Methods

The methodology for this scoping review was adapted from the Joanna Briggs Institute framework.^12^ The study protocol was registered on Open Science (https://doi.org/10.17605/OSF.IO/DTVYE).

### Search strategy

A search of the electronic databases MEDLINE, Embase and CINAHL from January 2000 to December 2023 was performed (online supplemental appendix 1). The beginning of the search period was January 2000 as infliximab, the first biologic disease-modifying antirheumatic drug (DMARD), was approved by the Food and Drug Administration in November 1999, making a new era in the management of CIRDs. After initial identification of a paucity of studies available from Oceania, Africa, South America and the Middle East, an additional manual search was conducted of Google Scholar using search terms adapted from the main search strategy using the same date restrictions.

### Selection criteria

Inclusion criteria included observational studies, including cross-sectional, cohort and case-control studies reporting on the epidemiology and associated factors for osteoporosis, falls and osteoporotic fractures in participants ≥18 years with RA, PsA, AS or SLE. Osteoporosis and falls and CIRD diagnoses of RA, PsA, AS and SLE of all definitions were included for comprehensiveness of the scoping review. The definitions provided by each study for CIRD diagnoses, osteoporosis, falls and fractures for each included study are provided in online supplemental appendix 4. Exclusion criteria included reviews, clinical guidelines, abstracts and non-English articles. Articles reporting on bone mineral density values only and traumatic fractures were also excluded.

### Study selection

Records obtained from searched electronic databases were transferred into Covidence systematic review software. Duplicates were removed, with titles and abstracts screened for relevance. A full-text review was performed by two authors (CC, GB) with exclusion of those which did not meet inclusion criteria. Articles obtained from the manual search, titles and abstracts were screened by author CC, with author GB verifying if inclusion criteria were met.

### Data extraction and synthesis

For included articles, two authors (CC, GB) extracted data to a pre-designed standard data extraction form (online supplemental appendix 2). Information included was study characteristics (date, country, methodology), population (gender, CIRD) and outcomes of interest (osteoporosis, falls, fractures and associated risk factors). Definitions of CIRDs and outcomes were also extracted. Differences in data were resolved through re-discussion by the two authors. Explored associated factors were categorised into demographic factors, disease-related, medications and other (including novel). Results were analysed descriptively.

### Patient and public involvement

No patients or consumer representatives were involved in the development, conduct or analysis of this review.

## Results

### Search results

**Figure 1:**
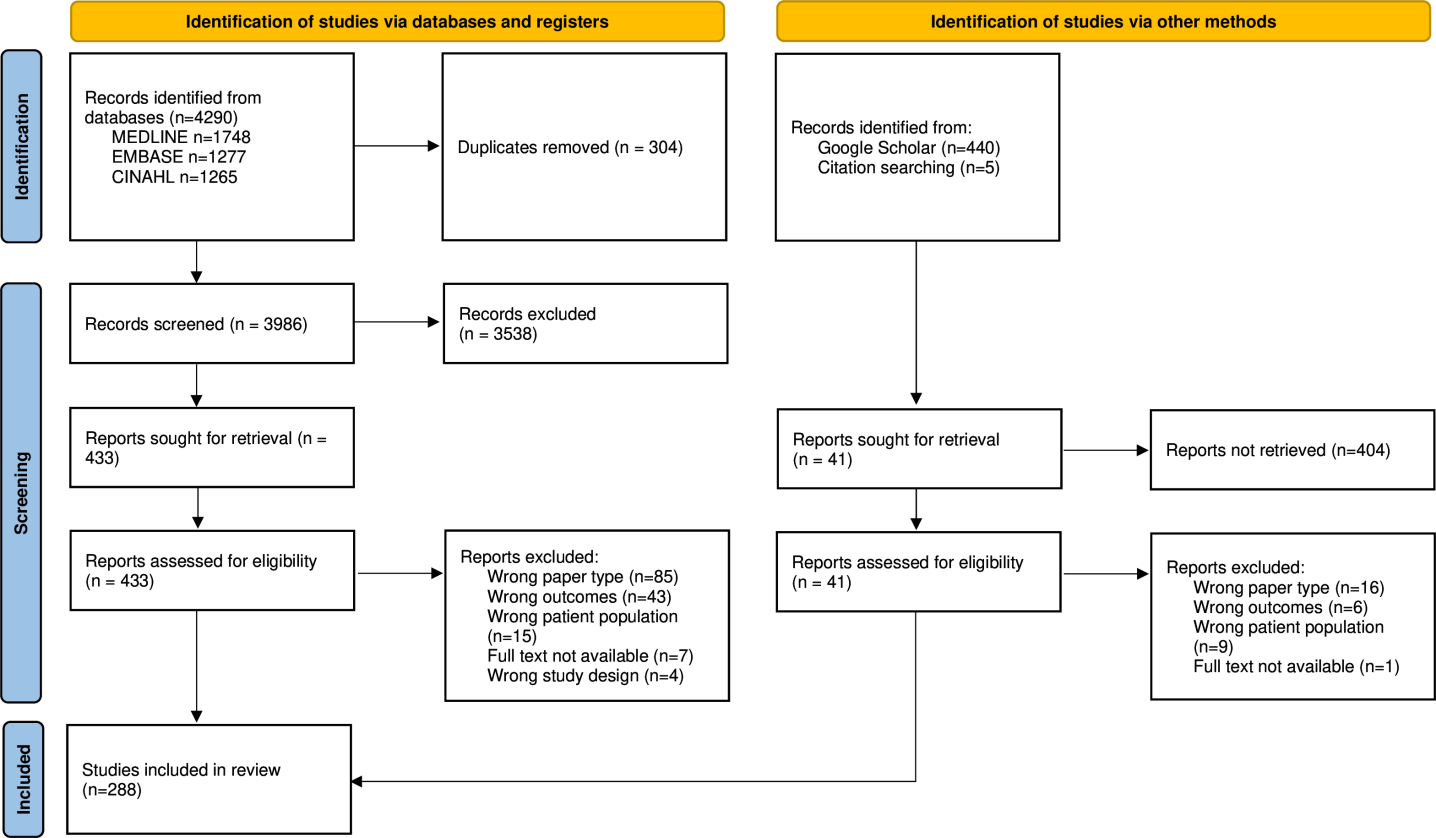
Article extraction flow chart.

After the exclusion of duplicates, 3986 citations were reviewed. 286 articles met inclusion criteria; however, the full text for seven articles was not available. An additional nine studies were identified from the manual search. 288 articles were included for full-text review (figure 1). All articles meeting inclusion criteria are summarised in online supplemental appendix 3.

### Study characteristics

Of the 288 articles, 280 reported on osteoporosis, falls and/or osteoporotic fractures in a single CIRD. Eight articles reported on ≥2 CIRDs. Table 1 summarises the characteristics of all included studies. RA was the most frequently explored rheumatic condition in relation to osteoporosis, falls and fractures (n=170), followed by SLE (n=60), AS (n=49) and PsA (n=19). Most articles were from Asia and Europe (28.8% and 37.8%, respectively). Cross-sectional was the most frequent study design (58.3%), followed by cohort (37.8%) and case control (3.8%). Of the 108 cohort studies, 60 were prospective and 48 were retrospective. The use of public or health insurance databases and disease registries was the most frequent method of recruitment, with three using data from clinical trials. There was a female predominance among study participants, particularly in RA and SLE.

**Table 1:** Number of studies reporting on osteoporosis, falls and fractures in CIRDs and study characteristics

	All (288)	RA (170)	PsA (19)	AS (49)	SLE (60)
Outcomes*					
Osteoporosis	160	84	13	29	38
Falls	27	24	2	1	0
Fractures	162	90	9	32	39
Publication year					
2000–2009	71 (24.7)	35 (20.59)	2 (10.5)	11 (22.45)	24 (40.0)
2010–2019	158 (54.9)	92 (54.12)	12 (63.2)	34 (69.39)	27 (45.0)
2020–2023	59 (20.5)	43 (25.29)	5 (26.3)	4 (8.16)	9 (15.0)
Geographic area					
Europe	109 (37.8)	54 (31.8)	9 (47.4)	33 (67.3)	19 (31.7)
UK	16 (5.6)	11 (6.5)	0 (0.0)	1 (2.0)	4 (6.7)
Asia	83 (28.8)	58 (34.1)	1 (5.3)	7 (14.3)	19 (31.7)
USA/Canada	33 (11.5)	15 (8.8)	4 (21.1)	4 (8.2)	11 (18.3)
South America	19 (6.6)	11 (6.5)	1 (5.3)	0 (0.0)	7 (11.7)
Australia/New Zealand	5 (1.7)	4 (2.4)	2 (10.5)	0 (0.0)	0 (0.0)
Middle East	7 (2.4)	5 (2.9)	1 (5.3)	1 (2.0)	0 (0.0)
Africa	16 (5.6)	12 (7.1)	1 (5.3)	3 (6.1)	0 (0.0)
Gender					
All	195 (67.7)	120 (70.6)	17 (89.5)	40 (81.6)	27 (45.0)
Female	79 (27.4)	47 (27.6)	2 (10.5)	8 (16.3)	31 (51.7)
Male	14 (4.9)	3 (1.8)	0 (0.0)	1 (2.0)	2 (3.3)
Methodology					
Cross-sectional	168 (58.3)	90 (52.9)	14 (73.7)	30 (61.2)	39 (65.0)
Cohort	109 (37.8)	72 (42.4)	5 (26.3)	15 (30.6)	20 (33.3)
Case control	11 (3.8)	8 (4.7)	0 (0.0)	4 (8.2)	1 (1.7)

Results reported in n (%).

* Multiple outcomes and CIRDs in papers.

AS, ankylosing spondylitis; CIRDs, chronic inflammatory rheumatic diseases; PsA, psoriatic arthritis; RA, rheumatoid arthritis; SLE, systemic lupus erythematosus.

### Definitions of osteoporosis, osteoporotic fractures and falls

CIRD and outcome definitions used by included studies are summarised in online supplemental appendix 4. The WHO definition of osteoporosis, with bone mineral density (BMD) ≤−2.5 below the average value for young healthy women (T score of ≤−2.5) was the most frequently used definition. Several retrospective cohort studies used diagnostic codes, including International Classification of Diseases (ICD) and READ codes to identify cases of osteoporosis. Less frequently used definitions of osteoporosis included Trabecular Bone Scores^13 14^ and lateral lumbar BMD values.^15^ One study used the clinical definition of osteoporosis, of either osteoporosis based on BMD or history of osteoporotic fracture.^16^ Twelve studies reported the definition of falls used, all following the Prevention of Falls Network Europe (ProFANE) definition of an unexpected event resulting in one coming to rest on the ground, floor or other level.^17^ There was heterogeneity in the reported outcome of osteoporotic fractures, in the methods used to identify fractures and in the fracture site. Methods ranged from unvalidated self-report to self-report validated by medical records and/or imaging and vertebral based on imaging and ICD codes.

### Rheumatoid arthritis

163 articles that described the epidemiology and/or associated factors for osteoporosis, fractures and falls in RA were identified (table 1). The 1987 American College of Rheumatology (ACR) criteria and 2010 ACR/EULAR criteria were common definitions used as inclusion criteria. Use of ICD, READ or alternate diagnostic codes, with or without verification with DMARD use, was strategies employed by retrospective cohort studies.^18^,^32^ 90 (52.9%) studies were cross-sectional, with 72 (42.2%) cohort studies, of which 44 (61.1%) were prospective. Associated factors explored in RA related to osteoporosis, fractures and falls are summarised in table 2, with relevant references available in online supplemental appendix 5. 84 (49.4%) articles in RA report on the epidemiology and/or associated factors for osteoporosis, 90 (52.9%) on fractures and 24 (14.1%) on fractures. Known demographic risk factors for osteoporosis, including age, gender, BMI and menopause status, were frequently reported, as were disease-specific risk factors including parameters of disease severity and related medications. Of the 24 papers on falls in RA, 13 (54.2%) were cross-sectional studies.

**Table 2:** Number of studies reporting on osteoporosis, fractures and falls and associated factors in RA

	Osteoporosis (84)	Fractures (90)	Falls (24)
Demographics			
Age	51 (60.7)	55 (61.1)	18 (75.0)
Gender	21 (25.0)	26 (28.9)	11 (45.8)
BMI	36 (42.9)	36 (40.0)	8 (33.3)
Ethnicity	0 (0.0)	3 (3.3)	1 (4.2)
Menopausal status	23 (27.4)	19 (21.1)	1 (4.2)
Smoking	14 (16.7)	25 (27.8)	2 (8.3)
Alcohol	3 (3.6)	7 (7.8)	0 (0.0)
Fracture history	9 (10.7)	21 (23.3)	2 (8.3)
Falls history	2 (2.4)	3 (3.3)	5 (20.8)
Physical activity	1 (1.2)	3 (3.3)	3 (12.5)
Marital status	0 (0.0)	1 (1.1)	2 (8.3)
Education attainment	0 (0.0)	2 (3.3)	1 (4.2)
Socioeconomic status	0 (0.0)	0 (0.0)	2 (8.3)
Medications			
Disease-specific			
Glucocorticoids	46 (54.8)	59 (65.6)	9 (37.5)
csDMARDs	12 (14.3)	21 (23.3)	3 (12.5)
bDMARDs	8 (9.5)	18 (20.0)	3 (12.5)
tsDMARDs	0 (0.0)	2 (2.2)	0 (0.0)
DMARDs, unspecified	6 (7.1)	7 (7.8)	1 (4.2)
Analgesics			
Acetaminophen	0 (0.0)	1 (1.1)	0 (0.0)
NSAIDs	4 (4.8)	6 (6.7)	0 (0.0)
Opioids	1 (1.2)	5 (5.6)	0 (0.0)
Osteoporosis-related			
Antiresorptive+/-anabolics	9 (10.7)	21 (23.3)	1 (4.2)
Calcium supplements	5 (6.0)	6 (6.7)	0 (0.0)
Vitamin D supplements	4 (4.8)	9 (10.0)	1 (4.2)
HRT	4 (4.8)	4 (4.4)	0 (0.0)
Other			
Antihypertensives	0 (0.0)	0 (0.0)	2 (8.3)
Diuretics	0 (0.0)	0 (0.0)	1
PPIs	0 (0.0)	7 (7.8)	0 (0.0)
Antidepressants	1 (1.2)	4 (4.4)	2 (8.3)
Psychotropics	0 (0.0)	2	2 (8.3)
Anticoagulants	0 (0.0)	1 (1.1)	1 (4.2)
Anticonvulsants	1 (1.2)	3 (3.3)	0 (0.0)
Oral hypoglycaemics	0 (0.0)	0 (0.0)	1 (4.2)
Thyroid hormone replacement	0 (0.0)	1 (1.1)	0 (0.0)
Disease related			
Disease duration	39 (46.2)	41 (45.6)	14 (58.3)
Disease activity	34 (40.5)	37 (41.1)	16 (66.7)
Inflammatory markers	25 (29.8)	21 (23.3)	4 (16.7)
Autoantibodies	35 (41.7)	27 (30.0)	3 (12.5)
Radiographic damage	8 (9.5)	6 (6.7)	0 (0.0)
Other			
Vitamin D level	7 (8.3)	4 (4.4)	1 (4.2)
Bone turnover markers	3 (3.6)	1 (1.1)	0 (0.0)
Mobility aids	1 (1.2)	1 (1.1)	3 (12.5)
Comorbidities	1 (1.2)	6 (6.7)	3 (12.5)
Mobility/balance tests	0 (0.0)	1 (1.1)	11 (45.8)

bDMARD, biologic DMARD; BMI, body mass index; csDMARD, conventional synthetic DMARD; DMARDs, disease-modifying antirheumatic drugs; HRT, hormone replacement therapy; NSAID, non-steroidal anti-inflammatory drug; PPI, proton pump inhibitor; RA, rheumatoid arthritis; tsDMARD, targeted synthetic DMARD.

Laboratory parameters, including serum albumin, calcium, phosphate, uric acid, parathyroid hormone (PTH) level, bone turnover markers and lipid profile were explored for potential associations with osteoporosis and fractures. Two studies reported on the association between vitamin D receptor and osteoprotegerin (OPG) polymorphisms, with tumour necrosis receptor associated factor 6 genotype associations in one study.^33 34^ Patient-related factors, including body composition, sarcopenia, foot and ankle characteristics and pain in relation to osteoporosis, fractures and falls were studied in three studies.^35^,^37^ Dizziness, visual impairment and vertigo as falls associated factors were studied in two papers.^38 39^

### Psoriatic arthritis

Of the 19 published articles in PsA, 14 (73.7%) were cross-sectional, with only five (26.3%) studies using a cohort design (table 1). All cohort studies were retrospective, using health databases. Six studies defined PsA as per the Classification for Psoriatic Arthritis criteria (17, 42, 97, 210, 221, 261), two as per the Moll and Wrist criteria (31, 74), from ICD or READ codes (119, 123, 124, 198, 224, 226) and four based on a physician diagnosis (91, 95, 187, 232). Two studies did not provide a definition for PsA for inclusion (43, 57). The number of studies exploring associated factors is summarised in table 3, with references available in online supplemental appendix 5. Thirteen explored factors associated with osteoporosis, nine with fractures and two with falls. Most studies exploring associated factors assessed disease-specific parameters, with disease duration, activity and inflammatory markers commonly explored. Three studies reported on the association between disease-related medications (glucocorticoids and DMARDs), osteoporosis and fractures.^40^,^42^ The two studies on falls were both cross-sectional studies, in which ‘falls’ was not clearly defined.^43 44^

**Table 3:** Number of studies reporting on osteoporosis, fractures and falls and associated factors in PsA

	Osteoporosis (13)	Fractures (9)	Falls (2)
Demographics			
Age	4 (30.8)	5 (55.6)	1 (50.0)
Gender	3 (23.1)	3 (33.3)	0 (0.0)
BMI	3 (23.1)	3 (33.3)	2 (100.0)
Ethnicity	1 (7.7)^80^	0 (0.0)	0 (0.0)
Menopausal status	4 (30.8)	3 (33.3)	1 (50.0)
Smoking	1 (7.7)	2 (22.2)	0 (0.0)
Medications			
Disease specific			N/A
Glucocorticoids	2 (15.4)	2 (15.4)	
csDMARDs	1 (7.7)	1 (11.1)	
bDMARDs	1 (7.7)	1 (11.1)	
NSAIDs	1 (7.7)	1 (11.1)	
Disease-related			
Disease duration	5 (38.5)	4 (44.4)	1 (50.0)
Disease activity	4 (30.8)	4 (44.4)	1 (50.0)
Inflammatory markers	5 (38.5)	3 (33.3)	0 (0.0)
Radiographic damage	1 (7.7)	1 (11.1)	0 (0.0)
Disease manifestations	2 (15.4)	3 (33.3)	0 (0.0)
Other			
Vitamin D level	1 (7.7)	0 (0.0)	0 (0.0)
Bone turnover markers	1 (7.7)	0 (0.0)	0 (0.0)
Comorbidities	0 (0.0)	0 (0.0)	1 (50.0)

bDMARDs, biologic disease-modifying antirheumatic drugs; BMI, body mass index; csDMARDs, conventional synthetic disease-modifying antirheumatic drugs; NSAIDs, non-steroidal anti-inflammatory drugs; PsA, psoriatic arthritis.

### Ankylosing spondylitis

Modified New York criteria and ICD codes were the common definitions employed as inclusion criteria for AS. Of the 49 papers exploring epidemiology and associated factors for osteoporosis, falls and fractures in AS, 29 examined osteoporosis and 32 fractures. The number of studies exploring associated factors is outlined in table 4, with references available in online supplemental appendix 5. One cross-sectional study from Turkey reported on falls in AS, assessing demographics and disease-related parameters as associated factors.^45^ Factors associated with osteoporosis and fractures explored in AS are summarised in table 4. Novel associations explored in limited studies include serum calcium, PTH level, tumour-necrosis factor alpha (TNF-α) and interleukin-6 (IL-6) concentrations.^14 15 46 47^ One study evaluated antioxidant markers in relation to osteoporosis.^48^

**Table 4:** Number of studies reporting on factors associated with osteoporosis, fractures and falls in AS

	Osteoporosis (29)	Fractures (32)	Falls (1)
Demographics			
Age	16 (55.2)	17 (53.1)	1 (100.0)
Gender	14 (48.3)	13 (40.6)	0 (0.0)
BMI	7 (24.1)	8 (25.0)	0 (0.0)
Ethnicity	2 (6.9)	1 (3.1)	0 (0.0)
Menopausal status	4 (13.8)	3 (9.4)	0 (0.0)
Smoking	4 (13.8)	5 (15.6)	0 (0.0)
Alcohol	1 (3.4)	2 (6.3)	0 (0.0)
Physical activity	3 (10.3)	2 (6.3)	0 (0.0)
Fracture history	2 (6.9)	3 (9.4)	0 (0.0)
Falls history	1 (3.4)	1 (3.1)	0 (0.0)
Marital status	1 (3.4)	1 (3.1)	0 (0.0)
Education attainment	1 (3.4)	1 (3.1)	0 (0.0)
Socioeconomic status	2 (6.9)	1 (3.1)	0 (0.0)
Medications			
Disease specific			
Glucocorticoids	4 (13.8)	5 (15.6)	0 (0.0)
csDMARDs	2 (6.9)	4 (12.5)	0 (0.0)
bDMARDs	8 (27.6)	6 (18.8)	0 (0.0)
DMARDs unspecified	0 (0.0)	2 (6.3)	0 (0.0)
NSAIDs	3 (10.3)	6 (18.8)	0 (0.0)
Osteoporosis-related			
Antiresorptive+/-anabolics	2 (6.9)	4 (12.5)	0 (0.0)
Calcium supplements	1 (3.4)	2 (6.3)	0 (0.0)
Vitamin D supplements	1 (3.4)	2 (6.3)	0 (0.0)
HRT	1 (3.4)	0 (0.0)	0 (0.0)
Psychotropics	0 (0.0)	1 (3.1)	0 (0.0)
Disease-related			
Disease duration	12 (41.4)	14 (43.8)	1 (100.0)
Disease activity	18 (62.1)	14 (43.8)	1 (100.0)
Inflammatory markers	19 (65.5)	14 (43.8)	1 (100.0)
HLA-B27 positivity	5 (17.2)	5 (15.6)	0 (0.0)
Radiographic damage	11 (37.9)	12 (37.5)	0 (0.0)
Spinal mobility	7 (24.1)	6 (18.8)	0 (0.0)
Disease manifestations	6 (20.7)	6 (18.8)	0 (0.0)
Other			
Vitamin D level	5 (17.2)	5 (15.6)	0 (0.0)
Bone turnover markers	7 (24.1)	4 (12.5)	0 (0.0)
Comorbidities	1 (3.4)	1 (3.1)	0 (0.0)

AS, ankylosing spondylitis; bDMARDs, biologic DMARDs; BMI, body mass index; csDMARDs, conventional synthetic DMARDs; DMARDs, disease-modifying antirheumatic drugs; HRT, hormone replacement therapy; NSAIDs, non-steroidal anti-inflammatory drugs.

### Systemic lupus erythematosus

Most papers were inclusive of SLE patients meeting ACR or the Systemic Lupus International Collaborating Clinics criteria (online supplemental appendix 4). Retrospective studies using data from existing datasets frequently used ICD codes. Four studies included SLE patients based on a clinician diagnosis only.^49^,^52^ Of the 60 studies in SLE, 38 papers reported on osteoporosis and 39 on fractures. There were no studies reporting on falls in SLE. Like other CIRDs, commonly reported associations were demographic risk factors and disease-related parameters and medications (table 5). References to relevant studies are provided in online supplemental appendix 5. Associations explored in relation to osteoporosis include sun exposure and sunscreen use, serum levels of albumin, calcium, phosphate and PTH and oestradiol index.^53^,^57^ Associations between receptor activator of nuclear factor kappa B ligand, OPG polymorphisms were explored in relation to fractures.^58^

**Table 5:** Number of studies reporting factors associated with osteoporosis, fractures and falls in SLE

	Osteoporosis (38)	Fractures (39)
Demographics		
Age	26 (68.4)	28 (71.8)
Gender	9 (23.7)	6 (15.4)
BMI	16 (42.1)	17 (43.6)
Ethnicity	10 (26.3)	8 (20.5)
Menopausal status	15 (39.5)	14 (35.9)
Smoking	15 (39.5)	15 (38.5)
Alcohol	6 (15.8)	8 (20.5)
Physical activity	8 (21.1)	4 (10.3)
Fracture history	3 (7.9)	5 (12.8)
Family history of OP	3 (7.9)	3 (7.7)
Falls history	1 (2.6)	0 (0.0)
Medications		
Disease specific		
Glucocorticoids	29 (76.3)	30 (76.9)
Antimalarials	11 (28.9)	13 (33.3)
Immunosuppressants	12 (31.6)	10 (25.6)
Osteoporosis-related		
Antiresorptive+/-anabolics	10 (26.3)	11 (28.2)
Calcium supplements	10 (26.3)	10 (25.6)
Vitamin D supplements	11 (28.9)	10 (25.6)
HRT	5 (13.2)	6 (15.4)
Other		
Diuretics	2 (5.3)	2 (5.1)
PPIs	1 (2.6)	1 (2.6)
Antidepressants	1 (2.6)	1 (2.6)
Psychotropics	1 (2.6)	1 (2.6)
Anticoagulants	2 (5.3)	3 (7.7)
Disease-related		
Disease duration	17 (44.7)	15 (38.5)
Disease activity	19 (50.0)	14 (35.9)
Damage accrual	16 (42.1)	13 (33.3)
Inflammatory markers	7 (18.4)	4 (10.3)
Autoantibodies	2 (5.3)	3 (7.7)
Disease manifestations	3 (7.9)	6 (15.4)
Other		
Vitamin D level	8 (21.1)	3 (7.7)
Bone turnover markers	5 (13.2)	1 (2.6)
Comorbidities	6 (15.8)	7 (17.9)

BMI, body mass index; HRT, hormone replacement therapy; OP, osteoporosis; PPIs, proton pump inhibitors; SLE, systemic lupus erythematosus.

## Discussion

This scoping review maps the available literature on the epidemiology and factors associated with osteoporosis, related fractures and falls in CIRD. RA was the most studied CIRD, with comparatively limited literature in spondyloarthropathies, PsA and AS. Studies on falls in CIRD were limited to small cross-sectional studies in RA, with only two papers in PsA and one in AS. There is discrepancy in the geographic distribution of studies, with most studies published across Western Europe, East Asia and North America, with a paucity of research from Oceania, Middle East, Sub-Saharan Africa and South America. There is marked methodological heterogeneity, with differences in definitions for the outcome measures of osteoporosis, falls and osteoporotic fractures. Due to the heterogeneity of study methodologies and case definitions, a comparison of the frequency of osteoporosis, osteoporotic fractures and falls in CIRDs is challenging and should be considered in the future design of observational studies and systematic reviews.

The greatest availability of literature regarding the epidemiology of osteoporosis, falls and fractures is in patients with RA. This is likely related to its high prevalence in comparison to other CIRDs, particularly affecting post-menopausal women and strong understanding of its pathophysiological influence on bone biology.^59^ This is in comparison to SLE, PsA and AS, in which the disease onset is younger, with spondyloarthropathies having a greater male predominance.^60^,^62^ Other possible contributors include the presence of disease registries and long-term observational studies for RA and SLE, in comparison to AS and PsA.^63^ This highlights the need for large longitudinal cohorts of patients with spondyloarthropathies evaluating osteoporosis, osteoporotic fractures and falls as outcomes, to better understand their epidemiology and potential risk factors. In addition, the heterogeneity of definitions of osteoporosis, fractures and falls, or the absence of a definition, leads to wide variability in reported frequencies and relevance of associated factors, highlighting the need to adopt a consensus definition for future observational studies to enable synthesis of findings.

Falls are a major risk factor for fractures, with 10–15% of falls in adults over 60 resulting in fractures, with half of minimal trauma fractures occurring in those who do not meet DEXA criteria for osteoporosis.^64^ Individuals with poorly controlled CIRD are theoretically at high falls risk, due to the impact of disease on pain and functional status, further compounded by increased use of centrally acting analgesics, antidepressants and psychotropics.^65^ Despite recognition of potential risk, we found limited studies reporting on falls and its associated factors. While recommendations exist for osteoporosis screening in CIRD, falls assessment is not included formally due to the absence of a universal standardised screening tool.^8 66^ This partly reflects the existing challenges with falls research and implementation, including case definitions, recall bias, methods of prospective reporting, and lack of dedicated falls databases alongside systemic and organisational barriers. Within the current available literature in RA, falls are more prevalent than in the general population, with markers of disease severity further compounding risk. In PsA, AS and SLE, the obstacles to falls research are compounded by the younger patient population and differing research priorities.^67 68^ Including falls data, using internationally recognised definitions, such as the ProFaNE definition for falls, robust methods of data collection for falls, in CIRD cohorts, may enable design of widely accepted predictive models, enabling identification of those at risk who would benefit from intervention.^17^

Factors influencing the disparity in geography in research availability may include different healthcare priorities and access to administrative health databases and resources. Administrative health databases have greater availability in countries which use a public healthcare system. Health research is inevitably influenced by financial resources, with the economic strength of Western European countries, the USA, Japan and China enabling high research output.^69^ Despite Australia and New Zealand having strong public health systems, a mismatch between the burden of musculoskeletal disease and research funding, in comparison to other priority health areas, may explain the lack of research output in this area.^70^ However, individuals with CIRD from developing countries have a higher likelihood of having a severe disease course, influenced by ethnic and genetic factors and external factors including low educational attainment, accessibility to medical care and treatment adherence.^71 72^ Compounded with additional unique factors, including the higher prevalence of multiparity, differences in dietary intake and food fortification, low- and middle-income countries carry significant worldwide burden of osteoporosis, which is only expected to increase with the global ageing population.^73 74^ Similar challenges may exist in immigrant populations in developed countries, a population frequently under-represented in health research. Our review found social determinants of health, including ethnicity, socioeconomic status and education, were infrequently included as factors potentially associated with osteoporosis, falls and fractures, despite being recognised as critical driving factors of health inequity and disease burden.^75^ There is a need for increased research from developing countries and inclusivity of diverse populations, particularly prospective studies, to enable individualised, comprehensive risk factor assessment inclusive of socioeconomic factors and management for preventive care.

Over the last 20 years, the therapeutic landscape of CIRD has rapidly evolved to include the use of bDMARDs and tsDMARDs, with significant improvements in disease outcomes. The expectation to improve comorbidity management has been reflected in practice guidelines and recommendations across several CIRDs, including the 2016 European League Against Rheumatism (EULAR) initiative.^8^ Despite the rapid uptake of advanced therapeutics in clinical practice, we found limited studies reported prospective data analysing the impact of advanced therapeutics on osteoporotic fractures and falls, translating to limited specific recommendations of osteoporosis management in CIRD.^76^ Similar challenges exist with cardiovascular disease in CIRD, another comorbidity which contributes to premature mortality in CIRDs.^77^ Currently, advanced therapeutics have comparable efficacy for disease control, thus, understanding the impact of DMARDs on bone health and other comorbidities may influence clinical decision making.^78^ Collection of prospective data on the use of therapeutics and osteoporotic fracture/falls outcomes, in addition to inclusion of fracture data in randomised controlled trial data, may provide further information to assist clinicians in therapeutic decision making. Increasing availability of large datasets may additionally assist in the development of artificial intelligence-enhanced predictive models for osteoporotic fractures and falls.

There are limitations to this scoping review. The search strategy employed was broad, deliberately to understand the breadth of literature available in this field. However, the review was limited to four inflammatory rheumatic diseases, with other inflammatory conditions such as non-SLE connective tissue disease and vasculitides carrying the burden of systemic inflammation and glucocorticoid use. Non-inflammatory rheumatic conditions, including osteoarthritis, have also been reported as associated with falls and fall-related injuries, highlighting the need for vigilance in managing patients with a range of rheumatic disease.^79^ Studies from low-middle-income countries were under-represented, with the inclusion of studies only in the English language, which may have failed to identify studies from these countries.

## Conclusion

Observational data available on the epidemiology and factors associated with osteoporosis and fractures are mostly from cross-sectional studies, based on populations from Western European countries, East Asia and the USA. Most available literature is in RA and SLE. Falls research is limited in CIRD, largely to cross-sectional studies in RA, despite falls carrying the significant burden of healthcare cost, individual morbidity and mortality. There is marked heterogeneity in definitions of osteoporosis, osteoporotic fractures and falls in currently available observational studies, raising challenges with comparison of reported frequencies in included studies. Future research should use large, prospective cohorts of CIRD patients, inclusive of patients from low-middle-income countries, inclusion of falls as an outcome with consistent definitions used for osteoporosis, osteoporotic fractures and falls. This may enable better understanding of the complex interaction between risk factors for osteoporosis, fractures and falls and enable development of disease-specific risk calculators which could guide preventive interventions.

## Supplementary material

10.1136/bmjopen-2024-096226 online supplemental file 1

10.1136/bmjopen-2024-096226 online supplemental file 2

10.1136/bmjopen-2024-096226 online supplemental file 3

10.1136/bmjopen-2024-096226 online supplemental file 4

10.1136/bmjopen-2024-096226 online supplemental file 5

## Data Availability

Data are available upon reasonable request.
