# Supplementary material for: Epidemiology and factors associated with osteoporosis, falls and fractures in patients with chronic inflammatory rheumatic disease: a scoping review
Source: BMJ Open. 2025 Jul 28;15(7):e096226. doi: 10.1136/bmjopen-2024-096226 (PMC12306236; doi:10.1136/bmjopen-2024-096226)
Supplement: online supplemental file 1 [file bmjopen-15-7-s001.pdf]

Appendix 1. Search Strategy

1.1 Medline via OVID

Ovid MEDLINE(R) ALL <1946 to December 14, 2023>

#	Search terms	Results
**1**	exp Osteoporosis/	63608
**2**	exp Accidental Falls/	28220
**3**	exp Fractures, Bone/	211050
**4**	(osteoporo* or osteopeni* or osteopaeni* or low bone mineral densit* or low bone densit* or fracture* or fall*).tw.	604756
**5**	exp Arthritis, Rheumatoid/	126978
**6**	arthritis, psoriatic/ or exp axial spondyloarthritis/ or exp spondylitis, ankylosing/	24267
**7**	exp Lupus Erythematosus, Systemic/	67761
**8**	rheumatoid arthritis or psoriatic arthritis or ankylosing spondylitis* or systemic lupus erythematosus* or rheumatoid or psoriatic or spondyloarthropathy or spondyloarthritis).tw.	218136
**9**	exp incidence/ or exp prevalence/	622819
**10**	exp Risk/	1394140
**11**	exp Epidemiology/ or exp Epidemiologic Studies/	3230083
**12**	(prevalence or incidence or risk* or epidemiol*).tw.	4300734
**13**	1 or 2 or 3 or 4	664742
**14**	5 or 6 or 7 or 8	281052
**15**	9 or 10 or 11 or 12	6636587
**16**	13 and 14 and 15	3922
**17**	limit 16 to (english language and humans and "all adult (19 plus years)" and last 23 years)	1748

1.2 Embase via OVID

Embase Classic+Embase <1947 to December 14, 2023>

#	Search terms	Results
**1**	exp Osteoporosis/	161921
**2**	exp Accidental Falls/	50923
**3**	exp Fractures, Bone/	399910
**4**	(osteoporo* or osteopeni* or osteopaeni* or low bone densit* or bone loss* or fracture* or fall*).tw.	855513
**5**	exp Arthritis, Rheumatoid/	264420
**6**	arthritis, psoriatic/ or exp axial spondyloarthritis/ or exp spondylitis, ankylosing/	54300
**7**	exp Lupus Erythematosus, Systemic/	121613
**8**	exp incidence/ or exp prevalence/	1624649
**9**	exp Risk/	3173021

**10**	exp Epidemiology/	4675758
**11**	(prevalence or incidence or risk or epidemiol*).tw.	6003402
**12**	1 or 2 or 3 or 4	1003414
**13**	5 or 6 or 7	400238
**14**	8 or 9 or 10 or 11	8897115
**15**	12 and 13 and 14	9921
**16**	limit 15 to (human and english language and (adult <18 to 64 years> or aged <65+ years>) and last 23 years)	1277

1.3 CINAHL via EBSCOHOST

https://search-ebscohost-com.ezproxy.library.sydney.edu.au/login.aspx?direct=true&AuthType=ip,shib&db=ccm&bquery=TX+(+osteoporo*+or+osteopeni*+or+osteopaeni*+or+%26quot%3bbone+loss%26quot%3b+or+%26quot%3blow+bone+density*%26quot%3b+or+fall*+or+fracture*+)+AND+TX+(+%26quot%3brheumatoid+arthritis%26quot%3b+or+%26quot%3bpsoriatic+arthritis%26quot%3b+or+%26quot%3bankylosing+spondylitis%26quot%3b+or+%26quot%3bsystemic+lupus+erythematosus%26quot%3b+or+rheumatoid+or+psoriatic+or+spondylitis+or+spondyloarthropathy+)+AND+TX+(+prevalence+or+incidence+or+epidemiolog*+or+risk*+)&cli0=DT1&clv0=200001-000001&cli1=LA1&clv1=Y&cli2=MX1&clv2=Y&cli3=CT2&clv3=Y&cli4=LA99&clv4=eng&cli5=AG3&clv5=All+Adult&type=1&searchMode=Standard&site=ehost-live&custid=s3382554

**Search terms**		**Results**
TX (osteoporo* or osteopeni* or osteopaeni* or "bone loss" or "low bone density*" or fall* or fracture*) AND TX ("rheumatoid arthritis" or "psoriatic arthritis" or "ankylosing spondylitis" or "systemic lupus erythematosus" or rheumatoid or psoriatic or spondylitis or spondyloarthropathy) AND TX (prevalence or incidence or epidemiolog* or risk*)	**Limiters** – Publication Date: 20000101-; English LanguageExclude MEDLINE records; Human; Language: English; Age Groups: All Adult<br>**Expanders -** Apply equivalent subjects<br>**Search modes -** Boolean/Phrase	1265

Total 4290 papers

1.4 Google Scholar

https://scholar.google.com/scholar?hl=en&as_sdt=0%2C5&as_ylo=2000&as_yhi=2023&q=%28osteoporosis+OR+fractures+OR+falls%29+%2B+%28rheumatoid+arthritis+or+psoriatic+arthritis+or+ankylosing+spondylitis+or+systemic+lupus+erythematosus%29+%2B+%28africa+OR+south+america+OR+australia+OR+new+zealand+OR+middle+east%29&btnG=

**Search terms**		**Results**
(osteoporosis OR fracture OR falls) AND (rheumatoid arthritis OR psoriatic arthritis OR systemic lupus erythematosus OR	where my words occur: anywhere in the article<br>Return articles dated between 2000-2023	440

ankylosing spondylitis) AND (Africa OR south America OR middle east OR Australia OR new zealand)		
