# Supplementary material for: Epidemiology and factors associated with osteoporosis, falls and fractures in patients with chronic inflammatory rheumatic disease: a scoping review
Source: BMJ Open. 2025 Jul 28;15(7):e096226. doi: 10.1136/bmjopen-2024-096226 (PMC12306236; doi:10.1136/bmjopen-2024-096226)
Supplement: online supplemental file 2 [file bmjopen-15-7-s002.pdf]

Appendix 2. Data extraction form

**Study details**	Publication year:<br>Country:<br>Study design:<br>If applicable:<br>Retrospective /<br>Prospective<br>Recruitment:				
**Population**	CIRD: RA / PsA / AS / SLE<br>Other inclusion criteria:<br>Gender: Female / Male / Both<br>Exclusion criteria:<br>Number:	Definition:			
**Outcomes**	Osteoporosis / Fractures / Falls	Definition:	Method of data collection:		
**Associated factors (Y/N)**	Demographics	Age<br>Gender<br>BMI<br>Menopausal status	Ethnicity<br>Marital status<br>Educational attainment<br>Socioeconomic status	Smoking<br>Alcohol<br>Physical activity	Fracture history<br>Falls history
	Disease-related	Disease activity<br>Disease duration<br>Damage accrual<br>Radiographic damage<br>Spinal mobility	Inflammatory markers<br>Autoantibodies<br>HLA-B27 status	Disease phenotype	
	Medications	Glucocorticoids<br>csDMARDs<br>bDMARDs<br>tsDMARDs<br>DMARDs unspecified<br>Anti-malarials<br>Other immunosuppressants	Acetaminophen<br>NSAIDs<br>Opioids	Anti-resorptives +/- anabolic therapy<br>Vitamin D supplementation<br>Calcium supplementation<br>HRT	Antihypertensives<br>PPIs<br>Diuretics<br>Psychotropics<br>Anti-depressants<br>Anticoagulants

	Other	Mobility/balance tests	Vitamin D level<br>BTM	Polypharmacy<br>Co-morbidities	Other
