# Supplementary material for: Epidemiology and factors associated with osteoporosis, falls and fractures in patients with chronic inflammatory rheumatic disease: a scoping review
Source: BMJ Open. 2025 Jul 28;15(7):e096226. doi: 10.1136/bmjopen-2024-096226 (PMC12306236; doi:10.1136/bmjopen-2024-096226)
Supplement: online supplemental file 4 [file bmjopen-15-7-s004.pdf]

Appendix 4. Definitions of osteoporosis, falls and osteoporotic fractures in included studies

**Reference**	**Definitions**						
	Osteoporosis	Falls	Fractures	RA	PsA	AS	SLE
**[1]**			ICD-10 codes including hip, clinically symptomatic vertebral, humerus, forearm, pelvis and rib				
**[2]**			ICD-10 codes including hip, clinically symptomatic vertebral, humerus, forearm, pelvis and rib	Diagnostic READ codes and DMARD prescriptions			
**[3]**			Non-vertebral fragility fractures including vertebral, pelvic, hip, femur, humerus, elbow, forearm, wrist, knee, tibia, fibula, foot or ankle using ICD codes	ICD-9 or ICD-10 diagnostic codes			

**[4]**	WHO definition			Clinical diagnosis			
**[5]**	NS			1987 ACR criteria			
**[6]**	WHO definition		Vertebral (based on imaging)				1987 ACR criteria
**[7]**			Vertebral (based on imaging)				1987 ACR criteria
**[8]**	WHO definition			2010 ACR/EULAR criteria			
**[9]**			All non-pathologic minimal trauma fractures	1987 ACR criteria			
**[10]**	WHO definition			2010 ACR/EULAR and 1987 ACR			
**[11]**			Vertebral (based on imaging)	1987 ACR criteria			
**[12]**			Hip	1987 ACR criteria			
**[13]**			Hip	1987 ACR criteria			
**[14]**	WHO definition			1987 ACR criteria			
**[15]**	WHO definition		Vertebral (based on imaging)			Modified New York criteria	
**[16]**		NS – exclusion of falls from road accidents and violence		NS			
**[17]**	NS			1987 ACR criteria			
**[18]**	WHO definition				CASPAR criteria		
**[19]**	WHO definition		All major osteoporotic including hip, wrist, pelvis and sacrum, ribs and sternum, humerus or femur, vertebral (imaging)	1987 ACR criteria			
**[20]**			Fragility, including closed hip, distal	ICD-10 diagnostic codes			

			radius/ulna, pelvis, humerus, femur, clinical vertebral as identified by ICD-9 codes				
**[21]**	WHO definition						1987 ACR criteria
**[22]**			Vertebral (imaging), non-vertebral (not defined)	1987 ACR criteria			
**[23]**	WHO definition						1987 ACR criteria
**[24]**	WHO definition		Vertebral (imaging)			Modified New York criteria	
**[25]**			Non-vertebral (excluding skull, fingers, toes)			ICD-10 diagnostic codes	
**[26]**	WHO definition		Fracture due to minor trauma (vertebral, forearm, pelvic, femur, humerus)				1987 ACR criteria
**[27]**	WHO definition						1987 ACR criteria
**[28]**		NS		1987 ACR criteria			
**[29]**			Vertebral (imaging)				1982 ACR criteria
**[30]**	WHO definition			1987 ACR criteria			
**[31]**			Vertebral (imaging)				1982 ACR criteria
**[32]**	WHO definition				Inflammatory arthritis with ≥1 joints associated with psoriasis, RF negative, spondylotic involvement excluded		
**[33]**	WHO definition		Vertebral (imaging)			Modified New York criteria	
**[34]**	WHO definition						1982 ACR criteria

**[35]**	WHO definition		Vertebral (imaging)	1987 ACR criteria			
**[36]**	NS						ICD-10 with relevant clinic visits
**[37]**			ICD-9 codes from original radiology reports	Rheumatologist diagnosed			
**[38]**		ProFaNE guidelines		2010 ACR/EULAR criteria			
**[39]**		ProFaNE guidelines		2010 ACR/EULAR criteria			
**[40]**		NS		1987 ACR criteria			
**[41]**			READ codes as osteoporotic (spine, hip, forearm, humerus)				NS
**[42]**	WHO definition		Vertebral (imaging)				1982 ACR criteria
**[43]**	WHO definition				CASPAR criteria		
**[44]**		NS			NS		
**[45]**	NS			ICD-10 codes and ACR/EULAR 1987 criteria			
**[46]**	WHO definition						1982 ACR criteria
**[47]**	WHO definition		NS	1987 ACR criteria			
**[48]**	WHO definition		Vertebral (imaging)	1987 ACR or 2010 ACR/EULAR criteria			
**[49]**	ICD-9 codes		ICD-9 codes (not further specified)				ICD-9 codes
**[50]**	WHO definition			1987 ACR			
**[51]**	WHO definition						1982 ACR criteria
**[52]**			Non-vertebral (humerus, wrist, pelvis, hip) using ICD-9 codes	ICD-9 codes and DMARD dispensing			
**[53]**	WHO definition						ACR or SLICC criteria

**[54]**			Hip, spine, wrist, ribs, pelvis, other (self-report)	Diagnosed by rheumatologist			
**[55]**	WHO definition		Symptomatic vertebral and peripheral fragility fractures (self-report)				Enrolled in university lupus clinic
**[56]**	ICD-9 codes			1987 ACR criteria or physician diagnosis with DMARD use			
**[57]**	WHO definition			1987 ACR criteria			
**[58]**			Vertebral (imaging), femoral neck or other non-vertebral from self-report		NS		
**[59]**	WHO definition		Vertebral (imaging)			Modified New York criteria	
**[60]**			Vertebral (symptomatic)	1987 ACR criteria			
**[61]**			NS				Confirmed "clinical diagnosis."
**[62]**			Vertebral (ICD-19 codes)			ICD-9 codes	
**[63]**	WHO definition			1987 ACR criteria			
**[64]**			Vertebral (imaging)	1987 ACR criteria			
**[65]**		NS				Modified New York criteria	
**[66]**			Symptomatic (including osteoporotic wrist, hip, rib, vertebral)_				1982 ACR criteria
**[67]**	WHO definition					Modified New York criteria	
**[68]**				1987 ACR criteria			

**[69]**	WHO definition		Vertebral (imaging)	1987 ACR criteria			
**[70]**		NS		2010 ACR/EULAR criteria			
**[71]**			Femur/hip, humerus, pelvis, radius/ulna, vertebrae (ICD-10)	ICD-10 codes			
**[72]**	WHO definition			2010 ACR/EULAR criteria			
**[73]**	WHO definition		Vertebral (imaging)	2010 ACR/EULAR criteria		Modified New York criteria	
**[74]**			Vertebral (self-report)			Self-reported, then confirmed by a doctor	
**[75]**	WHO and ISCD definition			1987 ACR criteria			
**[76]**	WHO definition		NS				1997 ACR criteria
**[77]**	NS				Weight and Moll criteria, peripheral arthritis with no axial involvement, seronegative for RF		
**[78]**			Vertebral (imaging)				1997 ACR criteria
**[79]**	WHO definition						1997 ACR criteria
**[80]**			Self-reported hip confirmed through review of medical records or radiographs	1987 ACR criteria			
**[81]**			Self-reported incident with mechanism eludicated through interview	1987 ACR criteria			
**[82]**			Self-reported vertebral and non-	1987 ACR criteria			

			vertebral (hip, wrist, humerus, pelvis, ribs) through review of medical records or radiographs				
**[83]**		NS		1987 ACR criteria			
**[84]**	WHO definition			2010 ACR/EULAR criteria			
**[85]**	NS			2010 ACR/EULAR criteria and/or 1987 ACR criteria			
**[86]**			Vertebral (imaging)	1987 ACR criteria			
**[87]**			Vertebral (imaging)				1997 ACR criteria
**[88]**	WHO definition		Fragility fractures, site not specified				Revised ACR criteria
**[89]**			Vertebral (imaging)				Diagnosed by a rheumatologist according to “national and international guidelines.”
**[90]**	WHO definition		Vertebral (imaging)	1987 ACR criteria			
**[91]**	WHO definition		Vertebral (imaging)			Modified New York criteria	
**[92]**	WHO definition		Self reported osteoporotic fractures (not further specified)	RA from registry (not otherwise specified)			1982 ACR criteria
**[93]**			Osteoporotic based on review of clinical records	2010 ACR/EULAR criteria			
**[94]**	WHO definition				Confirmed by two rheumatologists		
**[95]**	WHO definition					Modified New York criteria	

**[96]**			Vertebral (imaging)	2010 ACR/EULAR criteria			
**[97]**	WHO definition			1987 ACR criteria			
**[98]**	NS				PsA diagnosed by rheumatologist and/or hospital discharge record		
**[99]**	WHO definition			2010 ACR/EULAR criteria			
**[100]**	WHO definition				CASPAR criteria		
**[101]**	WHO definition			2010 ACR/EULAR criteria			
**[102]**	WHO definition			1987 ACR criteria			
**[103]**	WHO definition			1987 ACR criteria			
**[104]**	WHO definition			NS			
**[105]**		Unintentionally coming down on the floor to a lower level (PROFane)		"Definitive diagnosis"			
**[106]**			NS				1997 ACR criteria
**[107]**	WHO definition					Modified New York criteria	
**[108]**			Vertebral (imaging)	ICD-98-CM codes + identified cases using national database with diagnosis verified by two rheumatologists			
**[109]**	WHO definition		All patient reported fragility fractures	2010 ACR/EULAR criteria			
**[110]**	ICD-9-CM codes					ICD-9-CM codes + diagnosis received by a rheumatologist (using Taiwanese modified New York criteria)	
**[111]**	WHO definition			1987 ACR criteria			

**[112]**			Hip (hospital records)	1987 ACR criteria			
**[113]**	WHO definition			NS		NS	NS
**[114]**	WHO definition			2010 ACR/EULAR criteria			
**[115]**	WHO definition			Recorded diagnosis			
**[116]**	WHO definition			1987 ACR criteria			
**[117]**	NS			1987 ACR criteria			
**[118]**			Vertebral (medical records and imaging)	1987 ACR criteria			
**[119]**	WHO definition						1987 ACR criteria
**[120]**			Vertebral	2010 ACR/EULAR criteria			
**[121]**		NS	Osteoporotic (fractures resulting from minor injuries)	2010 ACR/EULAR criteria			
**[122]**			Vertebral (imaging)			Modified New York criteria	
**[123]**	NS		NS		ICD-9-CM diagnoses		
**[124]**			Vertebral (imaging)			Modified New York criteria	
**[125]**	WHO definition					Modified New York criteria	
**[126]**			ICD-10 codes for vertebral, femoral, pelvic, humeral	ICD-9 codes and at least one prescription of csDMARD or bDMARD			
**[127]**	NS		NS		ICD-9-CM codes		
**[128]**			Hip, humerus, radius/ulna, pelvic and vertebral excluding major trauma and pathologic	ICD-9 codes	ICD-9 codes	ICD-9 codes	
**[129]**	WHO definition	NS		1987 ACR criteria			

**[130]**	WHO definition			Clinical diagnosis of new onset RA meeting at least 6/10 2010 ACR criteria			
**[131]**			Vertebral (imaging)			NS	
**[132]**			Osteoporotic, as based on ICD-19-CM codes				ICD-10-CM codes
**[133]**			Osteoporotic, with major including vertebral or hip	1987 ACR criteria			
**[134]**			Vertebral (imaging)	NS			
**[135]**			ICD-10 codes for vertebral, hip, clavicle, humerus, wrist, ankle	RA diagnostic codes in insurance database			
**[136]**	NS		Hip, wrist, humerus, pelvis	ICD-9-CM codes			
**[137]**			Hip, wrist, humerus, pelvis	ICD-9-CM codes			
**[138]**	WHO definition		Vertebral (imaging)			Modified New York criteria	
**[139]**	WHO definition					Modified New York criteria	
**[140]**	WHO definition			2010 ACR/EULAR criteria			
**[141]**	WHO definition					Modified New York criteria	
**[142]**	WHO definition		Non-vertebral	1987 ACR criteria			
**[143]**	WHO definition			2010 ACR/EULAR criteria			
**[144]**			Vertebral (imaging)				2010 SLICC criteria
**[145]**			Vertebral, femoral, hip, distal radius, proximal humerus,	2010 ACR/EULAR criteria			

			distal radius, ribs, tibia-fibula, patella, pelvis				
**[146]**	WHO definition						1982 ACR
**[147]**	WHO definition			1987 ACR criteria			
**[148]**	BMD using QCT, OP defined as TBS<80mg/mL					Modified New York criteria	
**[149]**	BMD using QCT, OP defined as TBS<80mg/mL		Vertebral (imaging)			Modified New York criteria	
**[150]**	NS		ICD-9 codes for upper extremity, hip, lower extremity, vertebrae, stress				ICD-9 codes
**[151]**			Self-reported ankle, spine, rib, wrist, foot, pelvis, heel				1982 ACR criteria
**[152]**	NS			2010 ACR/EULAR criteria			
**[153]**	WHO definition			1987 ACR criteria			
**[154]**	WHO definition						1982 ACR criteria
**[155]**	WHO definition			1987 ACR criteria			
**[156]**	WHO definition		Vertebral (imaging)				1982 ACR criteria
**[157]**			Hip, based on ICD-9 or ICD-10 codes				ICD-9 or ICD-10 codes
**[158]**	Diagnosed by rheumatologist			Diagnosed by a rheumatologist			
**[159]**			Fragility fracture, of forearm, hip, pelvis or morphometric (vertebral	1987 ACR criteria or 2010 ACR/EULAR criteria			

			identified on imaging)				
**[160]**			Hip based on ICD-9-CM excluding pathologic, infection, mechanical complications	1987 ACR criteria			
**[161]**			Spinal and non-spinal (not further specified)			ICD-10 codes, confirmed using Modified New York criteria	
**[162]**	WHO definition		Vertebral (imaging)	1987 ACR criteria			
**[163]**		Self-report		1987 ACR criteria or 2010 ACR/EULAR criteria			
**[164]**		Unintentional displacement of the body to a level below the starting position, with the inability of correction in a timely manner, determined by multifactorial circumstances and affecting stability		1987 ACR criteria or 2010 ACR/EULAR criteria			
**[165]**			Spinal			ICD-9 codes	
**[166]**	ICD-10 codes		ICD-10 codes				
**[167]**			Vertebral (imaging)			Modified New York criteria	
**[168]**			Vertebral (imaging)			Modified New York criteria	
**[169]**	WHO definition		Hip and vertebral, osteoporotic as defined as fracture with low bone	2010 ACR/EULAR criteria			

			mass and non-violent mechanism				
**[170]**	WHO definition					Modified New York criteria	
**[171]**		NS, based on self-report		1987 ACR/EULAR criteria			
**[172]**			Vertebral (imaging)	2010 ACR/EULAR criteria			
**[173]**		Unintentionally coming to rest on the ground or at a lower level		1987 ACR criteria			
**[174]**	WHO definition			1987 ACR criteria			
**[175]**		NS		1987 ACR criteria			
**[176]**	WHO definition			1987 ACR criteria			
**[177]**			Incident clinical fragility fractures of vertebrae, femur, pelvis, ribs, distal radius and humerus	NS			
**[178]**	WHO definition		Vertebral (imaging)				1997 ACR or 2012 SLICC criteria
**[179]**	WHO definition		Vertebral (imaging)				1982 ACR criteria
**[180]**	ISCD consensus						1997 ACR or 2012 SLICC criteria
**[181]**	WHO definition		Vertebral (imaging)				1982 ACR criteria
**[182]**	WHO definition			1987 ACR criteria			
**[183]**	Lateral lumbar BMD value of 0.650g/cm2 taken as threshold, considered to correspond to T score of -2.5		Vertebral (imaging)			Modified New York criteria	

**[184]**		Unintentional fall to the ground, surface or a lower level relative to standing point.		1987 ACR criteria			
**[185]**	WHO definition			1987 ACR criteria			
**[186]**			Vertebral (imaging)			Modified New York criteria	
**[187]**	WHO definition		Vertebral (imaging)	1987 ACR criteria			
**[188]**	WHO definition		Vertebral (imaging)				1982 ACR criteria
**[189]**			Vertebral (imaging)				1997 ACR criteria
**[190]**	WHO definition						1997 ACR criteria
**[191]**	WHO definition			1987 ACR criteria			
**[192]**			Vertebral (imaging)			Modified New York criteria	
**[193]**			Clinical vertebral or non-vertebral (any site except skull, fingers and toes) using ICD-10 codes			ICD-10 codes	
**[194]**	WHO definition					Modified New York criteria	
**[195]**	WHO definition				Rheumatologist diagnosis		
**[196]**	WHO definition			1987 ACR criteria			
**[197]**			Vertebral and non-vertebral osteoporotic, including proximal humerus, distal radius, proximal femur, pelvic	1987 ACR criteria			
**[198]**			All insufficiency fractures, as	1987 ACR criteria			

			defined by fracture occurring in abnormal bone already weakened by decreased mineralisation and insufficient elastic resistance				
**[199]**	WHO definition			1987 ACR criteria			
**[200]**		NS		2010 ACR/EULAR criteria			
**[201]**			ICD-10 codes for osteoporotic fractures of forearm, upper arm and hip.	1987 ACR criteria			
**[202]**			Clinical vertebral and non-vertebral (ankle, arm, clavicle, elbow, foot, hand, knee, hip, leg, pelvis, rib, shoulder)	1987 ACR criteria			
**[203]**			Distal radius	1987 ACR criteria			
**[204]**			Non-vertebral including hip, distal radius, proximal humerus	1987 ACR criteria			
**[205]**	WHO definition			1987 ACR criteria			
**[206]**			All, hip, vertebral		READ codes from database		
**[207]**			Vertebral (imaging)	1987 ACR criteria			
**[208]**			Vertebral (imaging)	1987 ACR criteria			
**[209]**	WHO definition		Vertebral (imaging)	1987 ACR criteria			

**[210]**	WHO definition		Vertebral (imaging)	1987 ACR criteria			
**[211]**			Non-vertebral (wrist, humerus, ankle, tibia, hip) excluding those from trauma	1987 ACR criteria			
**[212]**			Vertebral (imaging)	1987 ACR criteria			
**[213]**	ICD-10-TM codes			ICD-10-TM diagnostic codes, under the care of rheumatologist and prescribed a DMARD			
**[214]**			Fracture – vertebral, hip, forearm	Diagnosis made by rheumatologist			
**[215]**	WHO definition			Diagnosis made by a physician			
**[216]**	WHO definition			1987 ACR criteria			
**[217]**	BMD<-2.5SD of young adult or radiologically evident osteoporotic fracture			1987 ACR criteria			
**[218]**		NS	Vertebral (imaging)		CASPAR criteria		
**[219]**	WHO definition			1987 ACR criteria			
**[220]**			Hip, using ICD-9 codes	ICD-9 codes			
**[221]**			Osteoporotic – any resulting from minimal trauma	1987 ACR or 2010 ACR/EULAR criteria			
**[222]**	WHO definition						Tertiary hospital lupus clinic patient

**[223]**			Non-traumatic vertebral and non-vertebral			ICD-8 and ICD-10 codes	
**[224]**	WHO definition						1982 ACR criteria
**[225]**	READ codes						READ codes for SLE
**[226]**			Vertebral (imaging)	2010 ACR/EULAR criteria			1982 ACR criteria
**[227]**	WHO definition			2010 ACR/EULAR criteria			
**[228]**			Symptomatic (hip, spine, upper arm, lower arm, hand, leg, fingers, foot, heel, pelvis, ribs, shoulder, wrist, ankles)				1982 ACR or 1997 ACR criteria
**[229]**	WHO definition		Fragility – vertebral or peripheral occurring spontaneously or caused by low impact trauma		CASPAR criteria		
**[230]**			Vertebral (imaging)			Modified New York criteria	
**[231]**	WHO definition	NS	Clinical and/or vertebral (including on imaging)	NS			
**[232]**	NS				ICD-9 and ICD-10 codes with visits to a rheumatologist		
**[233]**			Verified in medical record (site not specified)	2010 ACR/EULAR criteria			
**[234]**			Incident osteoporotic fractures using a		ICD-9 codes		

			RAMQ-validated algorithm				
**[235]**	NS			1987 ACR criteria			
**[236]**	WHO definition		Symptomatic fragility fractures				1997 ACR criteria
**[237]**	WHO definition			1987 ACR criteria			
**[238]**	WHO definition			2010 ACR/EULAR criteria			
**[239]**	WHO definition			1987 ACR criteria			
**[240]**	NS			Diagnosed by rheumatologist	Diagnosed by rheumatologist		
**[241]**		Any slip or trip in which balance is lost and landing on the floor, ground or lower level (following PROFANE recommendations)		Under the care of a rheumatologist			
**[242]**	WHO definition						1997 ACR criteria
**[243]**		An unexpected event in which participants come to rest on the ground, floor or other level (PROFANE recommendations)		2010 ACR/EULAR criteria			
**[244]**	WHO definition						1997 ACR criteria
**[245]**			Vertebral (imaging)	1987 ACR criteria			
**[246]**		Subject unintentionally coming to rest on the ground or at a lower level, including slipping from a bed or off a chair	Site not specified, based on self report	2010 ACR/EULAR criteria			

**[247]**			Pelvic, wrist, hip and humeral, based on diagnostic and procedure codes				ICD-9 codes
**[248]**	NS			NS			
**[249]**	WHO definition		Vertebral (imaging)	1987 ACR or 2010 ACR/EULAR criteria			
**[250]**	WHO definition		Vertebral (imaging)	1987 ACR or 2010 ACR/EULAR criteria			
**[251]**	WHO definition		Vertebral (imaging)			Modified New York criteria	
**[252]**			Hip (based on ICD-9 codes)			ICD-9 codes	
**[253]**	WHO definition						1982 ACR criteria
**[254]**	WHO definition		Vertebral (imaging)			Modified New York criteria	
**[255]**			Vertebral (imaging)	1987 ACR criteria			
**[256]**	WHO definition		Vertebral (imaging)			Modified New York criteria	
**[257]**			ICD-9 – clinical osteoporotic as defined as radius/ulna, humerus, rib, femur/hip, pelvis or vertebrae; or vertebral (imaging)	NS			
**[258]**	NS		Clinical, or radiographic vertebral	1987 ACR criteria			
**[259]**	WHO definition					Modified New York criteria	
**[260]**			ICD-8, site not further specified	Physician diagnosed, based on international criteria			

**[261]**	WHO definition		Non-vertebral low energy fractures and vertebral (imaging)	1987 ACR criteria			
**[262]**			Vertebral			Modified New York criteria	
**[263]**			ICD-9 codes for clinical fractures of radius/ulna, hip, vertebral			Modified New York criteria	
**[264]**	WHO definition			2010 ACR/EULAR criteria			
**[265]**	ICD-9 codes		ICD-9 codes for vertebral			ICD-9 codes	
**[266]**	WHO definition					Modified New York criteria	
**[267]**	WHO definition					Modified New York criteria	
**[268]**	WHO definition					Modified New York criteria	
**[269]**			Hip, based on ICD-9-CM codes				ICD-9-CM
**[270]**	WHO definition		Low trauma symptomatic fractures		CASPAR criteria		
**[271]**			Hip, vertebral based on ICD-9 and ICD-10 codes	ICD-9 and ICD-10 codes		ICD-9 and ICD-10 codes	ICD-9 and ICD-10 codes
**[272]**		Unexpected event in which participants come to rest on the ground, floor or lower level (ProFaNE definition)		2010 ACR/EULAR criteria			
**[273]**		Unexpected event in which participants come to rest on the ground, floor or		2010 ACR/EULAR criteria			

		lower level (ProFaNE definition)					
**[274]**	READ code for incident osteoporosis		READ code for bone fracture	READ codes			
**[275]**			All fractures; spine and hip verified on imaging	Self-reported RA + on DMARD			
**[276]**		NS		1987 ACR criteria			
**[277]**			Hip and radius, by ICD-10 codes	ICD-10 codes, verified by rheumatologist or internal medicine			
**[278]**	WHO definition						1982 ACR criteria
**[279]**	WHO definition		All fractures unrelated to trauma				1982 ACR criteria
**[280]**			Hip, excluding those related to major trauma	1987 ACR criteria			
**[281]**			Fragility fracture – not further specified	2010 ACR/EULAR criteria			
**[282]**	WHO definition		Fragility fracture	1987 ACR criteria			
**[283]**	WHO definition			Rheumatologist diagnosed			
**[284]**	WHO definition			2010 ACR/EULAR criteria			
**[285]**	WHO definition						1997 ACR criteria
**[286]**			Clinical vertebral or non-vertebral osteoporotic verified through medical records and/or radiographs				1997 ACR criteria
**[287]**	NS						NS

**[288]**		Unexpected event resulting in one to come to rest on the ground, floor or other lower level (ProFANE definition)		1987 ACR criteria			

*NS = not specified

Included studies

1. Abtahi S, Driessen JHM, Burden AM, Souverein PC, van den Bergh JP, van Staa TP, et al. Concomitant use of oral glucocorticoids and proton pump inhibitors and risk of osteoporotic fractures among patients with rheumatoid arthritis: a population-based cohort study. Annals of the Rheumatic Diseases. 2021;80(4):423-31.
2. Abtahi S, Driessen JHM, Burden AM, Souverein PC, Bergh JPvd, Staa TPv, et al. Low-dose oral glucocorticoid therapy and risk of osteoporotic fractures in patients with rheumatoid arthritis: a cohort study using the Clinical Practice Research Datalink. Rheumatology. 2022;61(4):1448-58.
3. Acurcio FA, Moura CS, Bernatsky S, Bessette L, Rahme E. Opioid Use and Risk of Nonvertebral Fractures in Adults with Rheumatoid Arthritis: A Nested Case-Control Study Using Administrative Databases. Arthritis and Rheumatology. 2016;68(1):83-91.
4. Aghaei M, Sedighi S, Behnampour N, Hezarkhani S, Shirashiani M, Mohebi EG, et al. Change in bone mineral density in post menopausal women with rheumatoid arthritis. Bangladesh Journal of Medical Science. 2013;12(2):158-63.
5. Al-Bishri J, Attar SM, Bassuni N, Al-Nofaiey Y, Qutbuddeen H, Al-Harthi S, et al. Comorbidity Profile Among Patients with Rheumatoid Arthritis and the Impact on Prescriptions Trend. Clinical Medicine Insights: Arthritis & Musculoskeletal Disorders. 2013(6):11-8.
6. Almehed K, Forsblad d'Elia H, Kvist G, Ohlsson C, Carlsten H. Prevalence and risk factors of osteoporosis in female SLE patients-extended report. Rheumatology. 2007;46(7):1185-90.
7. Almehed K, Hetenyi S, Ohlsson C, Carlsten H, Forsblad-d'Elia H. Prevalence and risk factors of vertebral compression fractures in female SLE patients. Arthritis Research and Therapy. 2010;12(4) (no pagination).
8. Alvarez-Cienfuegos A, Cantero-Nieto L, Garcia-Gomez JA, Callejas-Rubio JL, Gonzalez-Gay MA, Ortego-Centeno N. Association Between Homocysteine Serum Level and Bone Mineral Density in Patients With Rheumatoid Arthritis. Journal of Clinical Densitometry. 2020;23(4):561-7.
9. Amin S, Gabriel SE, Achenbach SJ, Atkinson EJ, Melton LJ, 3rd. Are young women and men with rheumatoid arthritis at risk for fragility fractures? A population-based study. Journal of Rheumatology. 2013;40(10):1669-76.
10. Amkreutz J, de Moel EC, Theander L, Willim M, Heimans L, Nilsson JA, et al. Association Between Bone Mineral Density and Autoantibodies in Patients With Rheumatoid Arthritis. Arthritis & Rheumatology. 2021;73(6):921-30.
11. Arai K, Hanyu T, Sugitani H, Murai T, Fujisawa J, Nakazono K, et al. Risk factors for vertebral fracture in menopausal or postmenopausal Japanese women with rheumatoid arthritis: A cross-sectional and longitudinal study. Journal of Bone and Mineral Metabolism. 2006;24(2):118-24.
12. Arai K, Hoshino M, Murai T, Fujisawa J, Kondo N, Netsu T, et al. Proximal femoral fracture in patients with rheumatoid arthritis. Modern Rheumatology. 2007;17(4):317-21.
13. Arai K, Suzuki N, Murayama T, Kondo N, Otsuka H, Koizumi M, et al. Age at the time of hip fracture in patients with rheumatoid arthritis is 4 years greater than it was 10 years before, but is still younger than that of the general population. Modern Rheumatology. 2020;30(1):64-9.

14. Arain SR, Riaz A, Nazir L, Umer TP, Rasool T. Low Bone Mineral Density among Patients with Newly Diagnosed Rheumatoid Arthritis. J Ayub Med Coll Abbottabad. 2016;28(1):175-8.
15. Arends S, Spoorenberg A, Bruyn GA, Houtman PM, Leijsma MK, Kallenberg CG, et al. The relation between bone mineral density, bone turnover markers, and vitamin D status in ankylosing spondylitis patients with active disease: a cross-sectional analysis. Osteoporosis International. 2011;22(5):1431-9.
16. Armstrong C, Swarbrick CM, Pye SR, O'Neill TW. Occurrence and risk factors for falls in rheumatoid arthritis. Annals of the Rheumatic Diseases. 2005;64(11):1602-4.
17. Aurrecoechea E, Llorca Diaz J, Diez Lizuain ML, McGwin G, Jr., Calvo-Alen J. Gender-associated comorbidities in rheumatoid arthritis and their impact on outcome: data from GENIRA. Rheumatology International. 2017;37(4):479-85.
18. Attia EA, Khafagy A, Abdel-Raheem S, Fathi S, Saad AA. Assessment of osteoporosis in psoriasis with and without arthritis: correlation with disease severity. Int J Dermatol. 2011;50(1):30-5.
19. Avouac J, Koumakis E, Toth E, Meunier M, Maury E, Kahan A, et al. Increased risk of osteoporosis and fracture in women with systemic sclerosis: a comparative study with rheumatoid arthritis. Arthritis care & research. 2012;64(12):1871-8.
20. Balasubramanian A, Wade S, Adler R, Lin C, Maricic M, O'Malley C, et al. Glucocorticoid exposure and fracture risk in patients with new-onset rheumatoid arthritis. Osteoporosis International. 2016;27(11):3239-49.
21. Banno S, Matsumoto Y, Naniwa T, Hayami Y, Sugiura Y, Yoshinouchi T, et al. Reduced bone mineral density in Japanese premenopausal women with systemic lupus erythematosus treated with glucocorticoids. Modern Rheumatology. 2002;12(4):323-8.
22. Baskan BM, Sivas F, Alemdaroglu E, Duran S, Ozoran K. Association of bone mineral density and vertebral deformity in patients with rheumatoid arthritis. Rheumatology International. 2007;27(6):579-84.
23. Becker A, Fischer R, Scherbaum WA, Schneider M. Osteoporosis screening in systemic lupus erythematosus: impact of disease duration and organ damage. Lupus. 2001;10(11):809-14.
24. Beek KJ, Rusman T, van der Weijden MAC, Lems WF, van Denderen JC, Konsta M, et al. Long-Term Treatment With TNF-Alpha Inhibitors Improves Bone Mineral Density But Not Vertebral Fracture Progression in Ankylosing Spondylitis. J Bone Miner Res. 2019;34(6):1041-8.
25. Bengtsson K, Askling J, Lorentzon M, Rosengren B, Deminger A, Klingberg E, et al. Occurrence and relative risks for non-vertebral fractures in patients with ankylosing spondylitis compared with the general population: a register-based study from Sweden. RMD Open. 2023;9(1):02.
26. Bhattoa HP, Bettembuk P, Balogh A, Szegedi G, Kiss E. Bone mineral density in women with systemic lupus erythematosus. Clinical Rheumatology. 2002;21(2):135-41.
27. Bhattoa HP, Kiss E, Bettembuk P, Balogh A. Bone mineral density, biochemical markers of bone turnover, and hormonal status in men with systemic lupus erythematosus. Rheumatology International. 2001;21(3):97-102.
28. Bohler C, Radner H, Ernst M, Binder A, Stamm T, Aletaha D, et al. Rheumatoid arthritis and falls: the influence of disease activity. Rheumatology. 2012;51(11):2051-7.
29. Bonfa AC, Seguro LP, Caparbo V, Bonfa E, Pereira RM. RANKL and OPG gene polymorphisms: associations with vertebral fractures and bone mineral density in premenopausal systemic lupus erythematosus. Osteoporosis International. 2015;26(5):1563-71.
30. Book C, Karlsson M, Akesson K, Jacobsson L. Disease activity and disability but probably not glucocorticoid treatment predicts loss in bone mineral density in women with early rheumatoid arthritis. Scandinavian Journal of Rheumatology. 2008;37(4):248-54.
31. Borba VZ, Matos PG, da Silva Viana PR, Fernandes A, Sato EI, Lazaretti-Castro M. High prevalence of vertebral deformity in premenopausal systemic lupus erythematosus patients. Lupus. 2005;14(7):529-33.
32. Borman P, Babaoglu S, Gur G, Bingol S, Bodur H. Bone mineral density and bone turnover in patients with psoriatic arthritis. Clinical Rheumatology. 2008;27(4):443-7.

33. Borman P, Bodur H, Bingol N, Bingol S, Bostan EE. Bone mineral density and bone turnover markers in a group of male ankylosing spondylitis patients: Relationship to disease activity. Journal of Clinical Rheumatology. 2001;7(5):315-21.
34. Boyanov M, Robeva R, Popivanov P. Bone mineral density changes in women with systemic lupus erythematosus. Clinical Rheumatology. 2003;22(4-5):318-23.
35. Breban S, Briot K, Kolta S, Paternotte S, Ghazi M, Fechtenbaum J, et al. Identification of rheumatoid arthritis patients with vertebral fractures using bone mineral density and trabecular bone score. Journal of Clinical Densitometry. 2012;15(3):260-6.
36. Bremander A, Petersson IF, Bergman S, Englund M. Population-based estimates of common comorbidities and cardiovascular disease in ankylosing spondylitis. Arthritis care & research. 2011;63(4):550-6.
37. Brennan SL, Toomey L, Kotowicz MA, Henry MJ, Griffiths H, Pasco JA. Rheumatoid arthritis and incident fracture in women: A case-control study. BMC Musculoskeletal Disorders. 2014;15(1) (no pagination).
38. Brenton-Rule A, Dalbeth N, Menz HB, Bassett S, Rome K. Foot and ankle characteristics associated with falls in adults with established rheumatoid arthritis: a cross-sectional study. BMC Musculoskeletal Disorders. 2016;17:22.
39. Brenton-Rule A, Dalbeth N, Menz HB, Bassett S, Rome K. Are Foot and Ankle Characteristics Associated With Falls in People With Rheumatoid Arthritis? A Prospective Study. Arthritis care & research. 2017;69(8):1150-5.
40. Bugdayci D, Paker N, Rezvani A, Kesiktas N, Yilmaz O, Sahin M, et al. Frequency and predictors for falls in the ambulatory patients with rheumatoid arthritis: A longitudinal prospective study. Rheumatology International. 2013;33(10):2523-7.
41. Bultink IE, Harvey NC, Lalmohamed A, Cooper C, Lems WF, van Staa TP, et al. Elevated risk of clinical fractures and associated risk factors in patients with systemic lupus erythematosus versus matched controls: a population-based study in the United Kingdom. Osteoporosis International. 2014;25(4):1275-83.
42. Bultink IE, Lems WF, Kostense PJ, Dijkmans BA, Voskuyl AE. Prevalence of and risk factors for low bone mineral density and vertebral fractures in patients with systemic lupus erythematosus. Arthritis & Rheumatism. 2005;52(7):2044-50.
43. Busquets N, Vaquero CG, Moreno JR, Vilaseca DR, Narvaez J, Carmona L, et al. Bone mineral density status and frequency of osteoporosis and clinical fractures in 155 patients with psoriatic arthritis followed in a university hospital. Reumatologia Clinica. 2014;10(2):89-93.
44. Carter K, Walmsley S, Oliffe M, Hassett G, Turner DE. Increased falls risk in people with psoriatic arthritis-related foot problems: a novel finding. Rheumatology. 2021;60(2):976-7.
45. Castillo-Canon JC, Trujillo-Caceres SJ, Bautista-Molano W, Valbuena-Garcia AM, Fernandez-Avila DG, Acuna-Merchan L. Rheumatoid arthritis in Colombia: a clinical profile and prevalence from a national registry. Clinical Rheumatology. 2021;40(9):3565-73.
46. Cervera R, Khamashta MA, Font J, Sebastiani GD, Gil A, Lavilla P, et al. Morbidity and mortality in systemic lupus erythematosus during a 10-year period: A comparison of early and late manifestations in a cohort of 1,000 patients. Medicine. 2003;82(5):299-308.
47. Cheng TT, Lai HM, Yu SF, Chiu WC, Hsu CY, Chen JF, et al. The impact of low-dose glucocorticoids on disease activity, bone mineral density, fragility fractures, and 10-year probability of fractures in patients with rheumatoid arthritis. Journal of Investigative Medicine. 2018;66(6):1004-7.
48. Cheng TT, Yu SF, Su FM, Chen YC, Su BYJ, Chiu WC, et al. Anti-CCP-positive patients with RA have a higher 10-year probability of fracture evaluated by FRAX: A registry study of RA with osteoporosis/fracture. Arthritis Research and Therapy. 2018;20(1) (no pagination).
49. Chen HL, Shen LJ, Hsu PN, Shen CY, Hall SA, Hsiao FY. Cumulative Burden of Glucocorticoid-related Adverse Events in Patients with Systemic Lupus Erythematosus: Findings from a 12-year Longitudinal Study. Journal of Rheumatology. 2018;45(1):83-9.
50. Chen J, Liu W, Lin Q, Chen L, Yin J, Huang H. Vitamin D deficiency and low bone mineral density in native Chinese rheumatoid arthritis patients. International Journal of Rheumatic Diseases. 2014;17(1):66-70.
51. Chong HC, Chee SS, Goh EM, Chow SK, Yeap SS. Dietary calcium and bone mineral density in premenopausal women with systemic lupus erythematosus. Clinical Rheumatology. 2007;26(2):182-5.

52. Cho SK, Lee JH, Han M, Lee S, Kim JY, Ryu JA, et al. The influence of vertebral fracture on the functional disability of patients with rheumatoid arthritis. Journal of Korean Medical Science. 2014;29(6):859-63.
53. Correa-Rodríguez M, Pocovi-Gerardino G, Callejas-Rubio J-L, Ríos-Fernández R, Rueda-Medina B, Ortego-Centeno N. Disease Damage Accrual and Low Bone Mineral Density in Female Patients with Systemic Lupus Erythematosus. Biological Research for Nursing. 2021;23(4):575-83.
54. Coulson KA, Reed G, Gilliam BE, Kremer JM, Pepmueller PH. Factors influencing fracture risk, T score, and management of osteoporosis in patients with rheumatoid arthritis in the Consortium of Rheumatology Researchers of North America (CORRONA) registry. JCR: Journal of Clinical Rheumatology. 2009;15(4):155-60.
55. Cramarossa G, Urowitz MB, Su J, Gladman D, Touma Z. Prevalence and associated factors of low bone mass in adults with systemic lupus erythematosus. Lupus. 2017;26(4):365-72.
56. Crowson CS, Gunderson TM, Dykhoff HJ, Myasoedova E, Atkinson EJ, Kronzer VL, et al. Comprehensive assessment of multimorbidity burden in a population-based cohort of patients with rheumatoid arthritis. RMD Open. 2022;8(1):01.
57. Dao HH, Do QT, Sakamoto J. Bone mineral density and frequency of osteoporosis among Vietnamese women with early rheumatoid arthritis. Clinical Rheumatology. 2011;30(10):1353-61.
58. Del Puente A, Esposito A, Costa L, Benigno C, Del Puente A, Foglia F, et al. Fragility Fractures in Patients with Psoriatic Arthritis. Journal of Rheumatology - Supplement. 2015;93:36-9.
59. Deminger A, Klingberg E, Lorentzon M, Geijer M, Gothlin J, Hedberg M, et al. Which measuring site in ankylosing spondylitis is best to detect bone loss and what predicts the decline: results from a 5-year prospective study. Arthritis research & therapy. 2017;19(1):273.
60. de Nijs RN, Jacobs JW, Bijlsma JW, Lems WF, Laan RF, Houben HH, et al. Prevalence of vertebral deformities and symptomatic vertebral fractures in corticosteroid treated patients with rheumatoid arthritis. Rheumatology. 2001;40(12):1375-83.
61. Dey M, Bukhari M. Predictors of fracture risk in patients with systemic lupus erythematosus. Lupus. 2018;27(9):1547-51.
62. Dhital R, Oke I, Donato A, Paudel A, Poudel DR, Paudel P, et al. Trends in hospitalizations for vertebral compression fracture in ankylosing spondylitis: data from the National Inpatient Sample 2000-2014. Clinical Rheumatology. 2021;40(12):4927-32.
63. di Munno O, Mazzantini M, Sinigaglia L, Bianchi G, Minisola G, Muratore M, et al. Effect of low dose methotrexate on bone density in women with rheumatoid arthritis: results from a multicenter cross-sectional study. Journal of Rheumatology. 2004;31(7):1305-9.
64. Dirven L, Van Den Broek M, Van Groenendael JHLM, De Beus WM, Kerstens PJSM, Huizinga TWJ, et al. Prevalence of vertebral fractures in a disease activity steered cohort of patients with early active rheumatoid arthritis. BMC Musculoskeletal Disorders. 2012;13 (no pagination).
65. Dursun N, Sarikaya S, Ozdolap S, Dursun E, Zateri C, Altan L, et al. Risk of falls in patients with ankylosing spondylitis. Journal of Clinical Rheumatology. 2015;21(2):76-80.
66. Ekblom-Kullberg S, Kautiainen H, Alha P, Leirisalo-Repo M, Julkunen H. Frequency of and risk factors for symptomatic bone fractures in patients with systemic lupus erythematosus. Scandinavian Journal of Rheumatology. 2013;42(5):390-3.
67. El Maghraoui A, Ebo'o FB, Sadni S, Majjad A, Hamza T, Mounach A. Is there a relation between pre-sarcopenia, sarcopenia, cachexia and osteoporosis in patients with ankylosing spondylitis? BMC Musculoskeletal Disorders. 2016;17:268.
68. El Maghraoui A, Rezqi A, Mounach A, Achemlal L, Bezza A, Ghozlani I. Prevalence and risk factors of vertebral fractures in women with rheumatoid arthritis using vertebral fracture assessment. Rheumatology. 2010;49(7):1303-10.
69. El Maghraoui A, Sadni S, Rezqi A, Bezza A, Achemlal L, Mounach A. Does Rheumatoid Cachexia Predispose Patients with Rheumatoid Arthritis to Osteoporosis and Vertebral Fractures? Journal of Rheumatology. 2015;42(9):1556-62.
70. Ergül EE, Melikoğlu MA. A quantitative assessment of the risk of falls in rheumatoid arthritis patients and determination of the risk factors. Turkish Journal of Physical Medicine & Rehabilitation (2587-1250). 2022;68(2):271-7.

71. Erwin J, Enki DG, Woolf AD. Younger people with rheumatoid arthritis are at increased risk of fracture even before age 50 years: a population-based cohort study. Osteoporosis International. 2021;32(8):1651-9.
72. Fadda S, Hamdy A, Abulkhair E, Elsify HM, Mostafa A. Serum levels of osteoprotegerin and RANKL in patients with rheumatoid arthritis and their relation to bone mineral density and disease activity. The Egyptian Rheumatologist. 2015;37(1):1-6.
73. Fauny M, Albuisson E, Bauer E, Perrier-Cornet J, Chary-Valckenaere I, Loeuille D. Study of vertebral fracture and Scanographic Bone Attenuation Coefficient in rheumatoid arthritis and ankylosing spondylitis vs. controls. Sci. 2019;9(1):13323.
74. Feldtkeller E, Vosse D, Geusens P, van der Linden S. Prevalence and annual incidence of vertebral fractures in patients with ankylosing spondylitis. Rheumatology International. 2006;26(3):234-9.
75. Filho JC, Pinheiro MM, de Moura Castro CH, Szejnfeld VL. Prevalence and risk factors associated with low-impact fractures in men with rheumatoid arthritis. Clinical Rheumatology. 2014;33(10):1389-95.
76. Franco CJV, Henao MPZ, Pineda FL, Montoya SP, Vallejo EO, Padilla LMR, et al. Low bone mass and osteoporosis in patients with systemic lupus erythematosus. Revista Colombiana de Reumatología (English Edition). 2017;24(1):4-10.
77. Frediani B, Allegri A, Falsetti P, Storri L, Bisogno S, Baldi F, et al. Bone mineral density in patients with psoriatic arthritis. Journal of Rheumatology. 2001;28(1):138-43.
78. Furukawa M, Kiyohara C, Horiuchi T, Tsukamoto H, Mitoma H, Kimoto Y, et al. Prevalence and risk factors of vertebral fracture in female Japanese patients with systemic lupus erythematosus. Modern Rheumatology. 2013;23(4):765-73.
79. Furukawa M, Kiyohara C, Tsukamoto H, Mitoma H, Kimoto Y, Uchino A, et al. Prevalence of and risk factors for low bone mineral density in Japanese female patients with systemic lupus erythematosus. Rheumatology International. 2011;31(3):365-76.
80. Furuya T, Inoue E, Hosoi T, Taniguchi A, Momohara S, Yamanaka H. Risk factors associated with the occurrence of hip fracture in Japanese patients with rheumatoid arthritis: a prospective observational cohort study. Osteoporosis International. 2013;24(4):1257-65.
81. Furuya T, Kotake S, Inoue E, Nanke Y, Yago T, Hara M, et al. Risk factors associated with incident fractures in Japanese men with rheumatoid arthritis: A prospective observational cohort study. Journal of Bone and Mineral Metabolism. 2008;26(5):499-505.
82. Furuya T, Kotake S, Inoue E, Nanke Y, Yago T, Kobashigawa T, et al. Risk factors associated with incident clinical vertebral and nonvertebral fractures in Japanese women with rheumatoid arthritis: a prospective 54-month observational study. Journal of Rheumatology. 2007;34(2):303-10.
83. Furuya T, Yamagiwa K, Ikai T, Inoue E, Taniguchi A, Momohara S, et al. Associated factors for falls and fear of falling in Japanese patients with rheumatoid arthritis. Clinical Rheumatology. 2009;28(11):1325-30.
84. Gabdulina G, Kasher M, Beissebayeva A, Mussabaeva D, Tokarev A, Mominova G, et al. An epidemiological analysis of osteoporotic characteristics in patients affected with rheumatoid arthritis in Kazakhstan. Archives of Osteoporosis. 2018;13(1):99.
85. Galarza-Delgado DA, Azpiri-Lopez JR, Colunga-Pedraza IJ, Cardenas-de la Garza JA, Vera-Pineda R, Wah-Suarez M, et al. Prevalence of comorbidities in Mexican mestizo patients with rheumatoid arthritis. Rheumatology International. 2017;37(9):1507-11.
86. Gamez-Nava JI, Ramirez-Villafana M, Cons-Molina F, Gomez-Ramirez EE, Esparza-Guerrero Y, Saldana-Cruz AM, et al. Serum irisin concentrations and osteoporotic vertebral fractures in women with rheumatoid arthritis: A cross-sectional study. Medicine. 2022;101(6):e28799.
87. Garcia-Carrasco M, Mendoza-Pinto C, Leon-Vazquez ML, Mendez-Martinez S, Etchegaray-Morales I, Montiel-Jarquin A, et al. Incidence of Vertebral Fractures in Women with Systemic Lupus Erythematosus After 8 Years of Follow-Up. Calcified Tissue International. 2017;101(3):291-9.
88. Garelick D, Pinto SM, Farinha F, Pires T, Khan E, Isenberg D. Fracture risk in systemic lupus erythematosus patients over 28 years. Rheumatology. 2021;60(6):2765-72.
89. Geusens P, De Winter L, Quaden D, Vanhoof J, Vosse D, van den Bergh J, et al. The prevalence of vertebral fractures in spondyloarthritis: relation to disease characteristics, bone mineral density, syndesmophytes and history of back pain and trauma. Arthritis research & therapy. 2015;17:294.

90. Ghazi M, Kolta S, Briot K, Fechtenbaum J, Paternotte S, Roux C. Prevalence of vertebral fractures in patients with rheumatoid arthritis: revisiting the role of glucocorticoids. Osteoporosis International. 2012;23(2):581-7.
91. Ghozlani I, Ghazi M, Nouijai A, Mounach A, Rezqi A, Achemlal L, et al. Prevalence and risk factors of osteoporosis and vertebral fractures in patients with ankylosing spondylitis. Bone. 2009;44(5):772-6.
92. Gilboe IM, Kvien TK, Haugeberg G, Husby G. Bone mineral density in systemic lupus erythematosus: comparison with rheumatoid arthritis and healthy controls. Annals of the Rheumatic Diseases. 2000;59(2):110-5.
93. Gomez-Vaquero C, Hernandez JL, Olmos JM, Cerda D, Calleja CH, Lopez JAM, et al. High incidence of clinical fragility fractures in postmenopausal women with rheumatoid arthritis. A case-control study. Bone. 2023;168:116654.
94. Grazio S, Cvijetic S, Vlak T, Grubisic F, Matijevic V, Nemcic T, et al. Osteoporosis in psoriatic arthritis: is there any? Wien Klin Wochenschr. 2011;123(23-24):743-50.
95. Grazio S, Kusic Z, Cvijetic S, Grubisic F, Balenovic A, Nemcic T, et al. Relationship of bone mineral density with disease activity and functional ability in patients with ankylosing spondylitis: a cross-sectional study. Rheumatology International. 2012;32(9):2801-8.
96. Guanabens N, Olmos JM, Hernandez JL, Cerda D, Hidalgo Calleja C, Martinez Lopez JA, et al. Vertebral fractures are increased in rheumatoid arthritis despite recent therapeutic advances: a case-control study. Osteoporosis International. 2021;32(7):1333-42.
97. Guler-Yuksel M, Bijsterbosch J, Goekoop-Ruiterman YP, de Vries-Bouwstra JK, Ronday HK, Peeters AJ, et al. Bone mineral density in patients with recently diagnosed, active rheumatoid arthritis. Annals of the Rheumatic Diseases. 2007;66(11):1508-12.
98. Haddad A, Ashkenazi RI, Bitterman H, Feldhamer I, Greenberg-Dotan S, Lavi I, et al. Endocrine Comorbidities in Patients with Psoriatic Arthritis: A Population-based Case-controlled Study. Journal of Rheumatology. 2017;44(6):786-90.
99. Hafez EA, Mansour HE, Hamza SH, Moftah SG, Younes TB, Ismail MA. Bone Mineral Density Changes in Patients with Recent-Onset Rheumatoid Arthritis. Clinical Medicine Insights: Arthritis & Musculoskeletal Disorders. 2011(4):87-94.
100. Halasi A, Szegedi A, Torocsik D, Varga J, Farmasi N, Szucs G, et al. Psoriatic arthritis and its special features predispose not only for osteoporosis but also for fractures and falls. Journal of Dermatology. 2023;50(5):608-14.
101. Hassine HB, Zemni R, Nacef IB, Boumiza A, Slama F, Baccouche K, et al. A TRAF6 genetic variant is associated with low bone mineral density in rheumatoid arthritis. Clinical Rheumatology. 2019;38(4):1067-74.
102. Haugeberg G, Uhlig T, Falch JA, Halse JI, Kvien TK. Bone mineral density and frequency of osteoporosis in female patients with rheumatoid arthritis: results from 394 patients in the Oslo County Rheumatoid Arthritis register. Arthritis & Rheumatism. 2000;43(3):522-30.
103. Haugen IK, Slatkowsky-Christensen B, Orstavik R, Kvien TK. Bone mineral density in patients with hand osteoarthritis compared to population controls and patients with rheumatoid arthritis. Annals of the Rheumatic Diseases. 2007;66(12):1594-8.
104. Hauser B, Riches PL, Wilson JF, Horne AE, Ralston SH. Prevalence and clinical prediction of osteoporosis in a contemporary cohort of patients with rheumatoid arthritis. Rheumatology. 2014;53(10):1759-66.
105. Hayashibara M, Hagino H, Katagiri H, Okano T, Okada J, Teshima R. Incidence and risk factors of falling in ambulatory patients with rheumatoid arthritis: a prospective 1-year study. Osteoporosis International. 2010;21(11):1825-33.
106. Heshin-Bekenstein M, Trupin L, Yelin E, von Scheven E, Yazdany J, Lawson EF. Longitudinal disease- and steroid-related damage among adults with childhood-onset systemic lupus erythematosus. Seminars in Arthritis and Rheumatism. 2019;49(2):267-72.
107. Hmamouchi I, Allali F, El Handaoui B, Amine H, Rostom S, Abouqal R, et al. The relation between disease activity, vitamin D levels and bone mineral density in men patients with ankylosing spondylitis. Rheumatology Reports. 2013;5(1):e3-e.
108. Hong WJ, Chen W, Yeo KJ, Huang PH, Chen DY, Lan JL. Increased risk of osteoporotic vertebral fracture in rheumatoid arthritis patients with new-onset cardiovascular diseases: a retrospective nationwide cohort study in Taiwan. Osteoporosis International. 2019;30(8):1617-25.

109. Huang H, Wang Y, Xie W, Geng Y, Gao D, Zhang Z. Impact of Treat-to-Target Therapy on Bone Mineral Density Loss in Patients With Rheumatoid Arthritis: A Prospective Cohort Study. Frontiers in Endocrinology. 2022;13:867610.
110. Hu LY, Lu T, Chen PM, Shen CC, Hung YM, Hsu CL. Should clinicians pay more attention to the potential underdiagnosis of osteoporosis in patients with ankylosing spondylitis? A national population-based study in Taiwan. PLoS ONE [Electronic Resource]. 2019;14(2):e0211835.
111. Hussien YM, Shehata A, Karam RA, Alzahrani SS, Magdy H, El-Shafey AM. Polymorphism in vitamin D receptor and osteoprotegerin genes in Egyptian rheumatoid arthritis patients with and without osteoporosis. Molecular Biology Reports. 2013;40(5):3675-80.
112. Huusko TM, Korpela M, Karppi P, Avikainen V, Kautiainen H, Sulkava R. Threefold increased risk of hip fractures with rheumatoid arthritis in Central Finland. Annals of the Rheumatic Diseases. 2001;60(5):521-2.
113. Hu Z, Xu S, Lin H, Ni W, Yang Q, Qi J, et al. Prevalence and risk factors for bone loss in Southern Chinese with rheumatic diseases. BMC Musculoskeletal Disorders. 2020;21(1) (no pagination).
114. Hu Z, Zhang L, Lin Z, Zhao C, Xu S, Lin H, et al. Prevalence and risk factors for bone loss in rheumatoid arthritis patients from South China: modeled by three methods. BMC Musculoskeletal Disorders. 2021;22(1) (no pagination).
115. Hwang J, Lee EK, Ahn J, Cha HS, Koh EM, Lee J. Bone-density testing interval and transition to osteoporosis in patients with rheumatoid arthritis. Osteoporosis International. 2017;28(1):231-7.
116. Ileana MC, Nelutu MA. Relationship Between Physical Disability, Disease Activity and Osteoporosis in Patients With Rheumatoid Arthritis. Archives of Rheumatology. 2014;29(4):273-9.
117. Innala L, Sjoberg C, Moller B, Ljung L, Smedby T, Sodergren A, et al. Co-morbidity in patients with early rheumatoid arthritis - inflammation matters. Arthritis research & therapy. 2016;18:33.
118. Ishida O, Furuya T, Inoue E, Ochi K, Ikari K, Taniguchi A, et al. Risk factors for established vertebral fractures in Japanese patients with rheumatoid arthritis: Results from a large prospective observational cohort study. Modern Rheumatology. 2015;25(3):373-8.
119. Jacobs J, Korswagen LA, Schilder AM, van Tuyl LH, Dijkmans BA, Lems WF, et al. Six-year follow-up study of bone mineral density in patients with systemic lupus erythematosus. Osteoporosis International. 2013;24(6):1827-33.
120. Jin S, Li M, Fang Y, Li Q, Liu J, Duan X, et al. Chinese Registry of rheumatoid arthritis (CREDIT): II. prevalence and risk factors of major comorbidities in Chinese patients with rheumatoid arthritis. Arthritis research & therapy. 2017;19(1):251.
121. Jin S, Li M, Wang Q, Zeng X, Xia W, Yu W, et al. Bone mineral density and microarchitecture among Chinese patients with rheumatoid arthritis: a cross-sectional study with HRpQCT. Arthritis Research and Therapy. 2021;23(1) (no pagination).
122. Jun JB, Joo KB, Her MY, Kim TH, Bae SC, Yoo DH, et al. Femoral bone mineral density is associated with vertebral fractures in patients with ankylosing spondylitis: a cross-sectional study. Journal of Rheumatology. 2006;33(8):1637-41.
123. Kaine J, Song X, Kim G, Hur P, Palmer JB. Higher Incidence Rates of Comorbidities in Patients with Psoriatic Arthritis Compared with the General Population Using U.S. Administrative Claims Data. J Manag Care Spec Pharm. 2019;25(1):122-32.
124. Kang KY, Kim IJ, Jung SM, Kwok SK, Ju JH, Park KS, et al. Incidence and predictors of morphometric vertebral fractures in patients with ankylosing spondylitis. Arthritis research & therapy. 2014;16(3):R124.
125. Karberg K, Zochling J, Sieper J, Felsenberg D, Braun J. Bone loss is detected more frequently in patients with ankylosing spondylitis with syndesmophytes. Journal of Rheumatology. 2005;32(7):1290-8.
126. Kasai S, Sakai R, Koike R, Kohsaka H, Miyasaka N, Harigai M. Higher risk of hospitalized infection, cardiovascular disease, and fracture in patients with rheumatoid arthritis determined using the Japanese health insurance database. Modern Rheumatology. 2019;29(5):788-94.
127. Kathuria P, Gordon KB, Silverberg JI. Association of psoriasis and psoriatic arthritis with osteoporosis and pathological fractures. J Am Acad Dermatol. 2017;76(6):1045-53.e3.

128. Kawai VK, Grijalva CG, Arbogast PG, Curtis JR, Solomon DH, Delzell E, et al. Initiation of tumor necrosis factor alpha antagonists and risk of fractures in patients with selected rheumatic and autoimmune diseases. Arthritis care & research. 2013;65(7):1085-94.
129. Kaz Kaz H, Johnson D, Kerry S, Chinappen U, Tweed K, Patel S. Fall-related risk factors and osteoporosis in women with rheumatoid arthritis. Rheumatology. 2004;43(10):1267-71.
130. Ketabforoush AHME, Aleahmad M, Qorbani M, Mehrpoor G, Afrashteh S, Mardi S, et al. Bone mineral density status in patients with recent-onset rheumatoid arthritis. Journal of Diabetes & Metabolic Disorders. 2023;22(1):775-85.
131. Killinger Z, Kuzma M, Tomkova S, Brazdilova K, Jackuliak P, Payer J. Prediction of Vertebral Fractures by Trabecular Bone Score in Patients With Ankylosing Spondylitis. Physiological research. 2021;70(Supplement 1):S53-S60.
132. Kim CS, Han KD, Jung JH, Choi HS, Bae EH, Ma SK, et al. Incidence and risk factors for osteoporotic fractures in patients with systemic lupus erythematosus versus matched controls. Korean Journal of Internal Medicine. 2021;36(1):154-63.
133. Kim D, Cho SK, Choi CB, Jun JB, Kim TH, Lee HS, et al. Incidence and risk factors of fractures in patients with rheumatoid arthritis: an Asian prospective cohort study. Rheumatology International. 2016;36(9):1205-14.
134. Kim D, Cho SK, Kim JY, Choi YY, Sung YK. Association between trabecular bone score and risk factors for fractures in Korean female patients with rheumatoid arthritis. Modern Rheumatology. 2016;26(4):540-5.
135. Kim D, Cho SK, Park B, Jang EJ, Bae SC, Sung YK. Glucocorticoids Are Associated with an Increased Risk for Vertebral Fracture in Patients with Rheumatoid Arthritis. Journal of Rheumatology. 2018;45(5):612-20.
136. Kim SY, Schneeweiss S, Liu J, Daniel GW, Chang CL, Garneau K, et al. Risk of osteoporotic fracture in a large population-based cohort of patients with rheumatoid arthritis. Arthritis research & therapy. 2010;12(4):R154.
137. Kim SY, Schneeweiss S, Liu J, Solomon DH. Effects of disease-modifying antirheumatic drugs on nonvertebral fracture risk in rheumatoid arthritis: a population-based cohort study. J Bone Miner Res. 2012;27(4):789-96.
138. Klingberg E, Geijer M, Gothlin J, Mellstrom D, Lorentzon M, Hilme E, et al. Vertebral fractures in ankylosing spondylitis are associated with lower bone mineral density in both central and peripheral skeleton. Journal of Rheumatology. 2012;39(10):1987-95.
139. Klingberg E, Lorentzon M, Mellstrom D, Geijer M, Gothlin J, Hilme E, et al. Osteoporosis in ankylosing spondylitis - prevalence, risk factors and methods of assessment. Arthritis research & therapy. 2012;14(3):R108.
140. Klodzinski L, Wislowska M. Comorbidities in rheumatic arthritis. Reumatologia. 2018;56(4):228-33.
141. Korczowska I, Przepiera-Bedzak H, Brzosko M, Lacki JK, Trefler J, Hrycaj P. Bone tissue metabolism in men with ankylosing spondylitis. Advances in Medical Sciences. 2011;56(2):264-9.
142. Kvien TK, Haugeberg G, Uhlig T, Falch JA, Halse JI, Lems WF, et al. Data driven attempt to create a clinical algorithm for identification of women with rheumatoid arthritis at high risk of osteoporosis. Annals of the Rheumatic Diseases. 2000;59(10):805-11.
143. Kweon SM, Sohn DH, Park JH, Koh JH, Park EK, Lee HN, et al. Male patients with rheumatoid arthritis have an increased risk of osteoporosis: Frequency and risk factors. Medicine (United States). 2018;97(24) (no pagination).
144. Lai EL, Huang WN, Chen HH, Chen JP, Chen DY, Hsieh TY, et al. Degraded microarchitecture by low trabecular bone score is associated with prevalent vertebral fractures in patients with systemic lupus erythematosus. Archives of Osteoporosis. 2020;15(1):54.
145. Lai EL, Huang WN, Chen HH, Hsu CY, Chen DY, Hsieh TY, et al. Ten-year fracture risk by FRAX and osteoporotic fractures in patients with systemic autoimmune diseases. Lupus. 2019;28(8):945-53.
146. Lakshminarayanan S, Walsh S, Mohanraj M, Rothfield N. Factors associated with low bone mineral density in female patients with systemic lupus erythematosus. Journal of Rheumatology. 2001;28(1):102-8.
147. Lala V, Tikly M, Musenge E, Govind N. Comorbidities in Black South Africans with established rheumatoid arthritis. International Journal of Rheumatic Diseases. 2022;25(6):699-704.

148. Lange U, Kluge A, Strunk J, Teichmann J, Bachmann G. Ankylosing spondylitis and bone mineral density--what is the ideal tool for measurement? Rheumatology International. 2005;26(2):115-20.
149. Lange U, Teichmann J, Strunk J, Muller-Ladner U, Schmidt KL. Association of 1.25 vitamin D3 deficiency, disease activity and low bone mass in ankylosing spondylitis. Osteoporosis International. 2005;16(12):1999-2004.
150. Le B, Waller JL, Radhakrishnan R, Oh SJ, Kheda MF, Nahman NS, Jr., et al. Osteoporotic fractures in patients with systemic lupus erythematosus and end stage renal disease. Lupus. 2018;27(1):17-24.
151. Lee C, Almagor O, Dunlop DD, Manzi S, Spies S, Ramsey-Goldman R. Self-reported fractures and associated factors in women with systemic lupus erythematosus. Journal of Rheumatology. 2007;34(10):2018-23.
152. Lee JH, Suh YS, Koh JH, Jung SM, Lee JJ, Kwok SK, et al. The risk of osteoporotic fractures according to the FRAX model in Korean patients with rheumatoid arthritis. Journal of Korean Medical Science. 2014;29(8):1082-9.
153. Lee JH, Sung YK, Choi CB, Cho SK, Bang SY, Choe JY, et al. The frequency of and risk factors for osteoporosis in Korean patients with rheumatoid arthritis. BMC Musculoskeletal Disorders. 2016;17(1) (no pagination).
154. Lee JJ, Aghdassi E, Cheung AM, Morrison S, Cymet A, Peeva V, et al. Ten-year absolute fracture risk and hip bone strength in Canadian women with systemic lupus erythematosus. Journal of Rheumatology. 2012;39(7):1378-84.
155. Lee SG, Park YE, Park SH, Kim TK, Choi HJ, Lee SJ, et al. Increased frequency of osteoporosis and BMD below the expected range for age among South Korean women with rheumatoid arthritis. International Journal of Rheumatic Diseases. 2012;15(3):289-96.
156. Li EK, Tam LS, Griffith JF, Zhu TY, Li TK, Li M, et al. High prevalence of asymptomatic vertebral fractures in Chinese women with systemic lupus erythematosus. Journal of Rheumatology. 2009;36(8):1646-52.
157. Li L, Xie H, Lu N, Esdaile JM, Avina-Zubieta JA. Impact of Systemic Lupus Erythematosus on the Risk of Newly Diagnosed Hip Fracture: A General Population-Based Study. Arthritis care & research. 2021;73(2):259-65.
158. Lindner L, Callhoff J, Alten R, Krause A, Ochs W, Zink A, et al. Osteoporosis in patients with rheumatoid arthritis: trends in the German National Database 2007-2017. Rheumatology International. 2020;40(12):2005-12.
159. Lin PH, Yu SF, Chen JF, Chen YC, Lai HM, Chiu WC, et al. Risk factor analysis of fragility fractures in rheumatoid arthritis: A 3-year longitudinal, real-world, observational, cohort study. PLoS ONE [Electronic Resource]. 2021;16(8):e0255542.
160. Lin YC, Li YH, Chang CH, Hu CC, Chen DW, Hsieh PH, et al. Rheumatoid arthritis patients with hip fracture: a nationwide study. Osteoporosis International. 2015;26(2):811-7.
161. Ljung L, Sundstrom B, Smeds J, Ketonen M, Forsblad-d'Elia H. Patterns of comorbidity and disease characteristics among patients with ankylosing spondylitis-a cross-sectional study. Clinical Rheumatology. 2018;37(3):647-53.
162. Lodder MC, Haugeberg G, Lems WF, Uhlig T, Orstavik RE, Kostense PJ, et al. Radiographic damage associated with low bone mineral density and vertebral deformities in rheumatoid arthritis: the Oslo-Truro-Amsterdam (OSTRA) collaborative study. Arthritis & Rheumatism. 2003;49(2):209-15.
163. Lourenco MA, Carli F, de Assis MR. Characterization of falls in adults with established rheumatoid arthritis and associated factors. Advances in Rheumatology. 2018;58(1):16.
164. Lourenco MDA, Roma I, Assis MRD. Falls and their association with physical tests, functional capacity, clinical and demographic factors in patients with rheumatoid arthritis. Revista Brasileira de Reumatologia. 2017;57(3):217-23.
165. Lukasiewicz AM, Bohl DD, Varthi AG, Basques BA, Webb ML, Samuel AM, et al. Spinal fracture in patients with ankylosing spondylitis. Spine. 2016;41(3):191-6.
166. Luque Ramos A, Redeker I, Hoffmann F, Callhoff J, Zink A, Albrecht K. Comorbidities in Patients with Rheumatoid Arthritis and Their Association with Patient-reported Outcomes: Results of Claims Data Linked to Questionnaire Survey. Journal of Rheumatology. 2019;46(6):564-71.

167. Maas F, Spoorenberg A, Brouwer E, Schilder AM, Chaudhry RN, Wink F, et al. Radiographic vertebral fractures develop in patients with ankylosing spondylitis during 4 years of TNF-alpha blocking therapy. Clin Exp Rheumatol. 2016;34(2):191-9.
168. Maas F, Spoorenberg A, van der Slik BPG, van der Veer E, Brouwer E, Bootsma H, et al. Clinical Risk Factors for the Presence and Development of Vertebral Fractures in Patients With Ankylosing Spondylitis. Arthritis care & research. 2017;69(5):694-702.
169. Ma CC, Xu SQ, Gong X, Wu Y, Qi S, Liu W, et al. Prevalence and risk factors associated with glucocorticoid-induced osteoporosis in Chinese patients with rheumatoid arthritis. Archives of Osteoporosis. 2017;12(1):33.
170. Magrey MN, Lewis S, Asim Khan M. Utility of DXA scanning and risk factors for osteoporosis in ankylosing spondylitis-A prospective study. Seminars in Arthritis and Rheumatism. 2016;46(1):88-94.
171. Mahdi MA, Erraoui M, Ngeuleu A, Tahiri L, Rkain H, Allali F. Falls, fear of falling and risk of falls in patients with rheumatoid arthritis: prevalence and associated factors. The Pan African Medical Journal. 2017;28(16).
172. Majjad A, Ghassem MA, Toufik H, Sadni S, Debbarh Z, Djossou JH, et al. Relationship between vertebral fracture prevalence and abdominal aortic calcification in women with rheumatoid arthritis. Bone. 2020;141:115599.
173. Mamoto K, Inui K, Okano T, Sugioka Y, Tada M, Koike T, et al. Incidence rate of falls and its risk factors in patients with rheumatoid arthritis compared to controls: Four years of the TOMORROW study. Modern Rheumatology. 2017;27(1):8-14.
174. Manrique F, Gamardo J, De Elguezabal K, Martinis R, Castro JS, Bellorin-Font E, et al. Abnormalities of bone mineral density and bone metabolism in Venezuelan patients with rheumatoid arthritis. Journal of Clinical Rheumatology. 2003;9(4):219-27.
175. Marques WV, Cruzb VA, Rego J, da Silva NA. The influence of physical function on the risk of falls among adults with rheumatoid arthritis. Revista Brasileira de Reumatologia. 2014;54(5):404-8.
176. Mazzantini M, Di Munno O, Sinigaglia L, Bianchi G, Rossini M, Mela Q, et al. Effect of cyclosporine A on bone density in female rheumatoid arthritis patients: results from a multicenter, cross-sectional study. Clin Exp Rheumatol. 2007;25(5):709-15.
177. Mazzantini M, Talarico R, Doveri M, Consensi A, Cazzato M, Bazzichi L, et al. Incident comorbidity among patients with rheumatoid arthritis treated or not with low-dose glucocorticoids: a retrospective study. Journal of Rheumatology. 2010;37(11):2232-6.
178. Mendoza-Pinto C, Etchegaray-Morales I, Munguia-Realpozo P, Mendez-Martinez S, Ayon-Aguilar J, Arellano-Avendano F, et al. SLICC-Frailty Index and Its Association with Low Bone Mineral Density and Vertebral Fractures in Women with Systemic Lupus Erythematosus. Calcified Tissue International. 2023;113(5):475-80.
179. Mendoza-Pinto C, Garcia-Carrasco M, Jimenez-Hernandez M, Sanchez-Perez R, Escarcega RO, Nava-Zavala A, et al. Carotid atherosclerosis is not associated with lower bone mineral density and vertebral fractures in patients with systemic lupus erythematosus. Lupus. 2015;24(1):25-31.
180. Mendoza-Pinto C, Garcia-Carrasco M, Juarez-Melchor D, Munguia-Realpozo P, Etchegaray-Morales I, Santiago-Martin N, et al. A Retrospective Analysis of Longitudinal Changes in Bone Mineral Density in Women with Systemic Lupus Erythematosus. Calcified Tissue International. 2021;109(4):363-71.
181. Mendoza-Pinto C, Garcia-Carrasco M, Sandoval-Cruz H, Munoz-Guarneros M, Escarcega RO, Jimenez-Hernandez M, et al. Risk factors of vertebral fractures in women with systemic lupus erythematosus. Clinical Rheumatology. 2009;28(5):579-85.
182. Meng J, Li Y, Yuan X, Lu Y. Evaluating osteoporotic fracture risk with the Fracture Risk Assessment Tool in Chinese patients with rheumatoid arthritis. Medicine (United States). 2017;96(18) (no pagination).
183. Mermerci Baskan B, Pekin Dogan Y, Sivas F, Bodur H, Ozoran K. The relation between osteoporosis and vitamin D levels and disease activity in ankylosing spondylitis. Rheumatology International. 2010;30(3):375-81.
184. Mikos M, Kucharska E, Lulek AM, Klosinski M, Batko B. Evaluation of Risk Factors for Falls in Patients with Rheumatoid Arthritis. Medical Science Monitor. 2020;26:e921862.
185. Mikuls TR, Saag KG, Curtis J, Bridges SL, Jr., Alarcon GS, Westfall AO, et al. Prevalence of osteoporosis and osteopenia among African Americans with early rheumatoid arthritis: the impact of ethnic-specific normative data. Journal of the National Medical Association. 2005;97(8):1155-60.

186. Mitra D, Elvins DM, Speden DJ, Collins AJ. The prevalence of vertebral fractures in mild ankylosing spondylitis and their relationship to bone mineral density. Rheumatology. 2000;39(1):85-9.
187. Mohammad A, Lohan D, Bergin D, Mooney S, Newell J, O'Donnell M, et al. The prevalence of vertebral fracture on vertebral fracture assessment imaging in a large cohort of patients with rheumatoid arthritis. Rheumatology. 2014;53(5):821-7.
188. Mok CC, Mak A, Ma KM. Bone mineral density in postmenopausal Chinese patients with systemic lupus erythematosus. Lupus. 2005;14(2):106-12.
189. Mok CC, Ying SKY, Chi HT, Kwok MM. Bone mineral density and body composition in men with systemic lupus erythematosus: A case control study. Bone. 2008;43(2):327-31.
190. Mok CC, Wong SN, Ma KM. Childhood-onset disease carries a higher risk of low bone mineral density in an adult population of systemic lupus erythematosus. Rheumatology. 2012;51(3):468-75.
191. Momohara S, Okamoto H, Yago T, Furuya T, Nanke Y, Kotake S, et al. The study of bone mineral density and bone turnover markers in postmenopausal women with active rheumatoid arthritis. Modern Rheumatology. 2005;15(6):410-4.
192. Montala N, Juanola X, Collantes E, Munoz-Gomariz E, Gonzalez C, Gratacos J, et al. Prevalence of vertebral fractures by semiautomated morphometry in patients with ankylosing spondylitis. Journal of Rheumatology. 2011;38(5):893-7.
193. Munoz-Ortego J, Vestergaard P, Rubio JB, Wordsworth P, Judge A, Javaid MK, et al. Ankylosing spondylitis is associated with an increased risk of vertebral and nonvertebral clinical fractures: a population-based cohort study. J Bone Miner Res. 2014;29(8):1770-6.
194. Muntean L, Rojas-Vargas M, Font P, Simon SP, Rednic S, Schiotis R, et al. Relative value of the lumbar spine and hip bone mineral density and bone turnover markers in men with ankylosing spondylitis. Clinical Rheumatology. 2011;30(5):691-5.
195. Muzevic MV, Biljan D, Sola M, Kuric I, Muzevic D, Mosler EL. Mineral Bone Density and Vitamin D Levels in Patients with Psoriatic Arthritis. Acta Clinica Croatica. 2022;61(1):70-8.
196. Nagayama Y, Ebina K, Tsuboi H, Hirao M, Hashimoto J, Yoshikawa H, et al. Low serum albumin concentration is associated with increased risk of osteoporosis in postmenopausal patients with rheumatoid arthritis. Journal of Orthopaedic Science. 2022;27(6):1283-90.
197. Nakayama M, Furuya T, Inoue E, Tanaka E, Ikari K, Yamanaka H, et al. Vitamin D deficiency is a risk factor for new fractures in Japanese postmenopausal women with rheumatoid arthritis: results from the IORRA cohort study. Archives of Osteoporosis. 2021;16(1):119.
198. Nampei A, Hashimoto J, Koyanagi J, Ono T, Hashimoto H, Tsumaki N, et al. Characteristics of fracture and related factors in patients with rheumatoid arthritis. Modern Rheumatology. 2008;18(2):170-6.
199. Nava-Valdivia CA, Ponce-Guarneros JM, Saldana-Cruz AM, Corona-Sanchez EG, Ramirez-Villafana M, Perez-Guerrero EE, et al. Assessment of Serum sRANKL, sRANKL/OPG Ratio, and Other Bone Turnover Markers with the Estimated 10-Year Risk of Major and Hip Osteoporotic Fractures in Rheumatoid Arthritis: A Cross-Sectional Study. BioMed Research International. 2021;2021:5567666.
200. Niksolat F, Zandieh Z, Roshani F, Larijani SS, Mirfakhraee H, Bahadori F, et al. Geriatric Syndromes among Patients with Rheumatoid Arthritis: A Comparison between Young and Elderly Patients. Ethiopian journal of health sciences. 2022;32(4):791-8.
201. Nyhall-Wahlin BM, Ajeganova S, Petersson IF, Andersson M. Increased risk of osteoporotic fractures in Swedish patients with rheumatoid arthritis despite early treatment with potent disease-modifying anti-rheumatic drugs: a prospective general population-matched cohort study. Scandinavian Journal of Rheumatology. 2019;48(6):431-8.
202. Ochi K, Furuya T, Ikari K, Taniguchi A, Yamanaka H, Momohara S. Sites, frequencies, and causes of self-reported fractures in 9,720 rheumatoid arthritis patients: a large prospective observational cohort study in Japan. Archives of Osteoporosis. 2013;8:130.
203. Ochi K, Go Y, Furuya T, Ikari K, Taniguchi A, Yamanaka H, et al. Risk factors associated with the occurrence of distal radius fractures in Japanese patients with rheumatoid arthritis: a prospective observational cohort study. Clinical Rheumatology. 2014;33(4):477-83.

204. Ochi K, Inoue E, Furuya T, Ikari K, Toyama Y, Taniguchi A, et al. Ten-year incidences of self-reported non-vertebral fractures in Japanese patients with rheumatoid arthritis: discrepancy between disease activity control and the incidence of non-vertebral fracture. Osteoporosis International. 2015;26(3):961-8.
205. Oelzner P, Schwabe A, Lehmann G, Eidner T, Franke S, Wolf G, et al. Significance of risk factors for osteoporosis is dependent on gender and menopause in rheumatoid arthritis. Rheumatology International. 2008;28(11):1143-50.
206. Ogdie A, Harter L, Shin D, Baker J, Takeshita J, Choi HK, et al. The risk of fracture among patients with psoriatic arthritis and psoriasis: a population-based study. Annals of the Rheumatic Diseases. 2017;76(5):882-5.
207. Okano T, Inui K, Tada M, Sugioka Y, Mamoto K, Wakitani S, et al. High frequency of vertebral fracture and low bone quality in patients with rheumatoid arthritis-Results from TOMORROW study. Modern Rheumatology. 2017;27(3):398-404.
208. Omata Y, Hagiwara F, Nishino J, Matsudaira K, Kadono Y, Juji T, et al. Vertebral fractures affect functional status in postmenopausal rheumatoid arthritis patients. Journal of Bone and Mineral Metabolism. 2014;32(6):725-31.
209. Orstavik RE, Haugeberg G, Mowinckel P, Hoiseth A, Uhlig T, Falch JA, et al. Vertebral deformities in rheumatoid arthritis: a comparison with population-based controls. Archives of Internal Medicine. 2004;164(4):420-5.
210. Orstavik RE, Haugeberg G, Uhlig T, Falch JA, Halse JI, Hoiseth A, et al. Vertebral deformities in 229 female patients with rheumatoid arthritis: associations with clinical variables and bone mineral density. Arthritis & Rheumatism. 2003;49(3):355-60.
211. Orstavik RE, Haugeberg G, Uhlig T, Mowinckel P, Falch JA, Halse JI, et al. Self reported non-vertebral fractures in rheumatoid arthritis and population based controls: incidence and relationship with bone mineral density and clinical variables. Annals of the Rheumatic Diseases. 2004;63(2):177-82.
212. Orstavik RE, Haugeberg G, Uhlig T, Mowinckel P, Falch JA, Halse JI, et al. Incidence of vertebral deformities in 255 female rheumatoid arthritis patients measured by morphometric X-ray absorptiometry. Osteoporosis International. 2005;16(1):35-42.
213. Osiri M, Sattayasomboon Y. Prevalence and out-patient medical costs of comorbid conditions in patients with rheumatoid arthritis. Joint Bone Spine. 2013;80(6):608-12.
214. Ozen G, Pedro S, Wolfe F, Michaud K. Medications associated with fracture risk in patients with rheumatoid arthritis. Annals of the Rheumatic Diseases. 2019;78(8):1041-7.
215. Palmowski A, Boyadzhieva Z, Nielsen SM, Muche B, Hermann S, Boers M, et al. Sex and age do not modify the association between glucocorticoids and bone mineral density in patients with rheumatoid arthritis: a cross-sectional study. Arthritis Research and Therapy. 2023;25(1) (no pagination).
216. Panopoulos S, Tektonidou M, Drosos AA, Liossis SN, Dimitroulas T, Garyfallos A, et al. Prevalence of comorbidities in systemic sclerosis versus rheumatoid arthritis: a comparative, multicenter, matched-cohort study. Arthritis research & therapy. 2018;20(1):267.
217. Panopoulos S, Thomas K, Georgiopoulos G, Boumpas D, Katsiari C, Bertsias G, et al. Comparable or higher prevalence of comorbidities in antiphospholipid syndrome vs rheumatoid arthritis: a multicenter, case-control study. Rheumatology. 2021;60(1):170-8.
218. Pedreira PG, Pinheiro MM, Szejnfeld VL. Bone mineral density and body composition in postmenopausal women with psoriasis and psoriatic arthritis. Arthritis research & therapy. 2011;13(1):R16.
219. Peng J, Gong Y, Zhang Y, Xiao Z, Zeng Q, Chen S. Bone Mineral Density in Patients With Rheumatoid Arthritis and 4-Year Follow-up Results. JCR: Journal of Clinical Rheumatology. 2016;22(2):71-4.
220. Peterkin-McCalman R, Waller JL, Le B, Oliver AM, Manning E, Elam RE, et al. Fractures in patients with rheumatoid arthritis and end-stage renal disease. Archives of Osteoporosis. 2020;15(1):146.
221. Phuan-Udom R, Lektrakul N, Katchamart W. The association between 10-year fracture risk by FRAX and osteoporotic fractures with disease activity in patients with rheumatoid arthritis. Clinical Rheumatology. 2018;37(10):2603-10.
222. Pineau CA, Urowitz MB, Fortin PJ, Ibanez D, Gladman DD. Osteoporosis in systemic lupus erythematosus: factors associated with referral for bone mineral density studies, prevalence of osteoporosis and factors associated with reduced bone density. Lupus. 2004;13(6):436-41.

223. Prieto-Alhambra D, Munoz-Ortego J, De Vries F, Vosse D, Arden NK, Bowness P, et al. Ankylosing spondylitis confers substantially increased risk of clinical spine fractures: a nationwide case-control study. Osteoporosis International. 2015;26(1):85-91.
224. Redlich K, Ziegler S, Kiener HP, Spitzauer S, Stohlawetz P, Bernecker P, et al. Bone mineral density and biochemical parameters of bone metabolism in female patients with systemic lupus erythematosus. Annals of the Rheumatic Diseases. 2000;59(4):308-10.
225. Rees F, Doherty M, Grainge M, Lanyon P, Davenport G, Zhang W. Burden of Comorbidity in Systemic Lupus Erythematosus in the UK, 1999-2012. Arthritis care & research. 2016;68(6):819-27.
226. Rentero ML, Amigo E, Chozas N, Fernandez Prada M, Silva-Fernandez L, Abad Hernandez MA, et al. Prevalence of fractures in women with rheumatoid arthritis and/or systemic lupus erythematosus on chronic glucocorticoid therapy. BMC Musculoskeletal Disorders. 2015;16:300.
227. Rexhepi S, Rexhepi M, Rexhepi B, Sahatciu-Meka V, Mahmutaj V. Bone Mineral Density in Patients With Early- Onset Rheumatoid Arthritis...2017 IOF-ESCEO World Congress on Osteoporosis. Medical Journal of Bakirkoy. 2020;16(1):44-8.
228. Rhew EY, Lee C, Eksarko P, Dyer AR, Tily H, Spies S, et al. Homocysteine, bone mineral density, and fracture risk over 2 years of followup in women with and without systemic lupus erythematosus. Journal of Rheumatology. 2008;35(2):230-6.
229. Riesco M, Manzano F, Font P, Garcia A, Nolla JM. Osteoporosis in psoriatic arthritis: an assessment of densitometry and fragility fractures. Clinical Rheumatology. 2013;32(12):1799-804.
230. Rossini M, Viapiana O, Idolazzi L, Ghellere F, Fracassi E, Troplini S, et al. Higher Level of Dickkopf-1 is Associated with Low Bone Mineral Density and Higher Prevalence of Vertebral Fractures in Patients with Ankylosing Spondylitis. Calcified Tissue International. 2016;98(5):438-45.
231. Rossini M, Viapiana O, Vitiello M, Malavolta N, La Montagna G, Maddali Bongi S, et al. Prevalence and incidence of osteoporotic fractures in patients on long-term glucocorticoid treatment for rheumatic diseases: The glucocorticoid induced OsTeoporosis TOol (GIOTTO) study. Reumatismo. 2017;69(1):30-9.
232. Ross Y, Jaleel S, Magrey M. Racial disparities in comorbidities of patients with psoriatic arthritis. Rheumatology International. 2023;43(8):1525-9.
233. Roubille C, Coffy A, Rincheval N, Dougados M, Flipo R-M, Daurès J-P, et al. Ten-year analysis of the risk of severe outcomes related to low-dose glucocorticoids in early rheumatoid arthritis. Rheumatology. 2021;60(8):3738-46.
234. Roussy JP, Bessette L, Bernatsky S, Rahme E, Lachaine J. Biologic disease-modifying anti-rheumatic drugs and the risk of non-vertebral osteoporotic fractures in patients with rheumatoid arthritis aged 50 years and over. Osteoporosis International. 2013;24(9):2483-92.
235. Sahatciu-Meka V, Rexhepi S, Manxhuka-Kerliu S, Rexhepi M. Extra-articular manifestations of seronegative and seropositive rheumatoid arthritis. Bosnian Journal of Basic Medical Sciences. 2010;10(1):26-31.
236. Salman-Monte TC, Torrente-Segarra V, Munoz-Ortego J, Mojal S, Carbonell-Abello J. Prevalence and predictors of low bone density and fragility fractures in women with systemic lupus erythematosus in a Mediterranean region. Rheumatology International. 2015;35(3):509-15.
237. Sarkis KS, Salvador MB, Pinheiro MM, Silva RG, Zerbini CA, Martini LA. Association between osteoporosis and rheumatoid arthritisin women: A cross-sectional study. Sao Paulo Medical Journal. 2009;127(4):216-22.
238. Senosi MR, Fathi HM, Baki NMA, Zaki O, Magdy AM, Gheita TA. Bone mineral density, vitamin D receptor (VDR) gene polymorphisms, fracture risk assessment (FRAX), and trabecular bone score (TBS) in rheumatoid arthritis patients: connecting pieces of the puzzle. Clinical Rheumatology. 2022;41(5):1333-42.
239. Shankar S, Handa R, Aneja R, Marwaha V, Ammini AC, Aprajita V. Bone mineral density in Indian women with rheumatoid arthritis. Rheumatology International. 2009;29(4):377-81.
240. Sinnathurai P, Buchbinder R, Hill C, Lassere M, March L. Comorbidity in psoriatic arthritis and rheumatoid arthritis. Intern Med J. 2018;48(11):1360-8.
241. Smith TO, Clarke C, Dainty JR, Watts L, Yates M, Pomeroy VM, et al. Clinical and biomechanical factors associated with falls and rheumatoid arthritis: baseline cohort with longitudinal nested case–control study. Rheumatology. 2022;61(2):679-87.
242. Souto MI, Coelho A, Guo C, Mendonca LM, Pinheiro MF, Papi JA, et al. The prevalence of low bone mineral density in Brazilian patients with systemic lupus erythematosus and its relationship with the disease damage index and other associated factors. Journal of Clinical Densitometry. 2012;15(3):320-7.

243. Stanmore EK, Oldham J, Skelton DA, O'Neill T, Pilling M, Campbell AJ, et al. Risk factors for falls in adults with rheumatoid arthritis: a prospective study. Arthritis care & research. 2013;65(8):1251-8.
244. Sun Y-N, Feng X-Y, He L, Zeng L-X, Hao Z-M, Lv X-H, et al. Prevalence and Possible Risk Factors of Low Bone Mineral Density in Untreated Female Patients with Systemic Lupus Erythematosus. BioMed Research International. 2015;2015:1-7.
245. Suzuki A, Tamai K, Takahashi S, Yamada K, Inui K, Tada M, et al. Do rheumatoid arthritis patients have low back pain or radiological lumbar lesions more frequently than the healthy population? - Cross-sectional analysis in a cohort study with age and sex-matched healthy volunteers. Spine Journal. 2020;20(12):1995-2002.
246. Tada M, Yamada Y, Mandai K, Hidaka N. Relationships of the stand-up time to falls and fractures in patients with rheumatoid arthritis: Results from the CHIKARA study. International Journal of Rheumatic Diseases. 2021;24(2):246-53.
247. Tedeschi SK, Kim SC, Guan H, Costenbader KH, Grossman JM. Comparative Fracture Risks Among United States Medicaid Enrollees With and Those Without Systemic Lupus Erythematosus. Arthritis & Rheumatology. 2019;71(7):1141-6.
248. Tiippana-Kinnunen T, Kautiainen H, Paimela L, Leirisalo-Repo M. Co-morbidities in Finnish patients with rheumatoid arthritis: 15-year follow-up. Scandinavian Journal of Rheumatology. 2013;42(6):451-6.
249. Tong JJ, Xu SQ, Wang JX, Zong HX, Chu YR, Chen KM, et al. Interactive effect of sarcopenia and falls on vertebral osteoporotic fracture in patients with rheumatoid arthritis. Archives of Osteoporosis. 2021;16(1):145.
250. Tong JJ, Xu SQ, Zong HX, Pan MJ, Teng YZ, Xu JH. Prevalence and risk factors associated with vertebral osteoporotic fractures in patients with rheumatoid arthritis. Clinical Rheumatology. 2020;39(2):357-64.
251. Toussirot E, Michel F, Wendling D. Bone density, ultrasound measurements and body composition in early ankylosing spondylitis. Rheumatology. 2001;40(8):882-8.
252. Tsur AM, David P, Watad A, Nissan D, Cohen AD, Amital H. Ankylosing Spondylitis and the Risk of Hip Fractures: a Matched Cohort Study. Journal of General Internal Medicine. 2022;37(13):3283-8.
253. Uaratanawong S, Deesomchoke U, Lertmaharit S, Uaratanawong S. Bone mineral density in premenopausal women with systemic lupus erythematosus. Journal of Rheumatology. 2003;30(11):2365-8.
254. Ulu MA, Batmaz I, Dilek B, Cevik R. Prevalence of osteoporosis and vertebral fractures and related factors in patients with ankylosing spondylitis. Chinese Medical Journal. 2014;127(15):2740-7.
255. Ursum J, Britsemmer K, van Schaardenburg D, Lips PT, Dijkmans BA, Lems W. High prevalence of vertebral deformities in elderly patients with early rheumatoid arthritis. Annals of the rheumatic diseases. 2009;68(9):1512-3.
256. van der Weijden MA, van Denderen JC, Lems WF, Nurmohamed MT, Dijkmans BA, van der Horst-Bruinsma IE. Etanercept Increases Bone Mineral Density in Ankylosing Spondylitis, but Does Not Prevent Vertebral Fractures: Results of a Prospective Observational Cohort Study. Journal of Rheumatology. 2016;43(4):758-64.
257. van Staa TP, Geusens P, Bijlsma JW, Leufkens HG, Cooper C. Clinical assessment of the long-term risk of fracture in patients with rheumatoid arthritis. Arthritis & Rheumatism. 2006;54(10):3104-12.
258. van Tuyl LH, Boers M, Lems WF, Landewe RB, Han H, van der Linden S, et al. Survival, comorbidities and joint damage 11 years after the COBRA combination therapy trial in early rheumatoid arthritis. Annals of the Rheumatic Diseases. 2010;69(5):807-12.
259. Vasdev V, Bhakuni D, Garg MK, Narayanan K, Jain R, Chadha D. Bone mineral density in young males with ankylosing spondylitis. International Journal of Rheumatic Diseases. 2011;14(1):68-73.
260. Vestergaard P, Rejnmark L, Mosekilde L. Fracture risk associated with use of nonsteroidal anti-inflammatory drugs, acetylsalicylic acid, and acetaminophen and the effects of rheumatoid arthritis and osteoarthritis. Calcified Tissue International. 2006;79(2):84-94.

261. Vis M, Haavardsholm EA, Boyesen P, Haugeberg G, Uhlig T, Hoff M, et al. High incidence of vertebral and non-vertebral fractures in the OSTRA cohort study: a 5-year follow-up study in postmenopausal women with rheumatoid arthritis. Osteoporosis International. 2011;22(9):2413-9.
262. Vosse D, Feldtkeller E, Erlendsson J, Geusens P, van der Linden S. Clinical vertebral fractures in patients with ankylosing spondylitis. Journal of Rheumatology. 2004;31(10):1981-5.
263. Vosse D, Landewe R, van der Heijde D, van der Linden S, van Staa TP, Geusens P. Ankylosing spondylitis and the risk of fracture: results from a large primary care-based nested case-control study. Annals of the Rheumatic Diseases. 2009;68(12):1839-42.
264. Wafa H, Raja A, Dhia K, Nada B, Imene Z, Montacer KM. Risk factors associated with bone loss and occurrence of fragility fractures in rheumatoid arthritis patients. The Egyptian Rheumatologist. 2019;41(1):1-5.
265. Walsh JA, Song X, Kim G, Park Y. Evaluation of the comorbidity burden in patients with ankylosing spondylitis using a large US administrative claims data set. Clinical Rheumatology. 2018;37(7):1869-78.
266. Wang D, Hou Z, Gong Y, Chen S, Lin L, Xiao Z. Bone edema on magnetic resonance imaging is highly associated with low bone mineral density in patients with ankylosing spondylitis. PLoS ONE [Electronic Resource]. 2017;12(12):e0189569.
267. Wang DM, Zeng QY, Chen SB, Gong Y, Hou ZD, Xiao ZY. Prevalence and risk factors of osteoporosis in patients with ankylosing spondylitis: a 5-year follow-up study of 504 cases. Clin Exp Rheumatol. 2015;33(4):465-70.
268. Wang L, Gao L, Jin D, Wang P, Yang B, Deng W, et al. The Relationship of Bone Mineral Density to Oxidant/Antioxidant Status and Inflammatory and Bone Turnover Markers in a Multicenter Cross-Sectional Study of Young Men with Ankylosing Spondylitis. Calcified Tissue International. 2015;97(1):12-22.
269. Wang SH, Chang YS, Liu CJ, Lai CC, Chen WS, Chen TJ, et al. Association of systemic lupus erythematosus with a higher risk of cervical but not trochanteric hip fracture: a nationwide population-based study. Arthritis care & research. 2013;65(10):1674-81.
270. Wang Y, Song ZB, Deng XR, Zhang XH, Zhang ZL. Risk factors associated with osteoporosis and fracture in psoriatic arthritis. Chinese Medical Journal. 2021;134(21):2564-72.
271. Weiss RJ, Wick MC, Ackermann PW, Montgomery SM. Increased fracture risk in patients with rheumatic disorders and other inflammatory diseases -- a case-control study with 53,108 patients with fracture. Journal of Rheumatology. 2010;37(11):2247-50.
272. Wiegmann S, Armbrecht G, Borucki D, Buehring B, Buttgereit F, Detzer C, et al. Association between sarcopenia, physical performance and falls in patients with rheumatoid arthritis: a 1-year prospective study. BMC Musculoskeletal Disorders. 2021;22(1):885.
273. Wiegmann S, Armbrecht G, Borucki D, Buehring B, Buttgereit F, Detzer C, et al. Balance and prospective falls in patients with rheumatoid arthritis. BMC Musculoskeletal Disorders. 2022;23(1):549.
274. Wilson JC, Sarsour K, Gale S, Petho-Schramm A, Jick SS, Meier CR. Incidence and Risk of Glucocorticoid-Associated Adverse Effects in Patients With Rheumatoid Arthritis. Arthritis care & research. 2019;71(4):498-511.
275. Wright NC, Lisse JR, Walitt BT, Eaton CB, Chen Z. Arthritis increases the risk for fractures--results from the Women's Health Initiative. Journal of Rheumatology. 2011;38(8):1680-8.
276. Yamagiwa K, Iijima S, Furuya T, Ikai T, Inoue E, Taniguchi A, et al. Incidence of falls and fear of falling in Japanese patients with rheumatoid arthritis. Modern Rheumatology. 2011;21(1):51-6.
277. Yamamoto Y, Turkiewicz A, Wingstrand H, Englund M. Fragility Fractures in Patients with Rheumatoid Arthritis and Osteoarthritis Compared with the General Population. Journal of Rheumatology. 2015;42(11):2055-8.
278. Yeap SS, Fauzi AR, Kong NC, Halim AG, Soehardy Z, Rahimah S, et al. Influences on bone mineral density in Malaysian premenopausal systemic lupus erythematosus patients on corticosteroids. Lupus. 2009;18(2):178-81.
279. Yee CS, Crabtree N, Skan J, Amft N, Bowman S, Situnayake D, et al. Prevalence and predictors of fragility fractures in systemic lupus erythematosus. Annals of the Rheumatic Diseases. 2005;64(1):111-3.

280. Yoshida S, Ikari K, Furuya T, Toyama Y, Taniguchi A, Yamanaka H, et al. An osteoprotegerin gene polymorphism is associated with an increased risk of hip fracture in Japanese patients with rheumatoid arthritis: results from the IORRA Observational Cohort Study. PLoS ONE [Electronic Resource]. 2014;9(8):e104587.
281. Yoshii I, Sawada N, Chijiwa T, Kokei S. Impact of sustaining SDAI remission for preventing incident of bone fragility fracture in patient with rheumatoid arthritis. Annals of the Rheumatic Diseases. 2022;81(2):296-9.
282. Yuen SY, Rochwerg B, Ouimet J, Pope JE. Patients with scleroderma may have increased risk of osteoporosis. A comparison to rheumatoid arthritis and noninflammatory musculoskeletal conditions. Journal of Rheumatology. 2008;35(6):1073-8.
283. Zahid PM, Samreen S, Khan ZA, Salim B, Ahmed SN, Gul H. Factors associated with low bone mineral density in postmenopausal women with rheumatoid arthritis. Journal of the Pakistan Medical Association. 2022;72(5):891-5.
284. Zeng T, Tan L, Yu J, Wu Y. High density lipoprotein in rheumatoid arthritis: emerging role in predicting inflammation level and osteoporosis occurrence. Scandinavian Journal of Clinical & Laboratory Investigation. 2020;80(5):375-80.
285. Zhu L, Zhang J, Wang J, Lv X, Pu D, Wang Y, et al. Uncoupled bone remodeling is characteristic of bone damage in premenopausal women with new-onset systemic lupus erythematosus. Lupus. 2021;30(7):1116-23.
286. Zhu TY, Griffith JF, Au SK, Tang XL, Kwok AW, Leung PC, et al. Incidence of and risk factors for non-vertebral and vertebral fracture in female Chinese patients with systemic lupus erythematosus: a five-year cohort study. Lupus. 2014;23(9):854-61.
287. Zonana-Nacach A, Barr SG, Magder LS, Petri M. Damage in systemic lupus erythematosus and its association with corticosteroids. Arthritis & Rheumatism. 2000;43(8):1801-8.
288. Zonzini Gaino J, Barros Bertolo M, Silva Nunes C, de Morais Barbosa C, Sachetto Z, Davitt M, et al. Disease-related outcomes influence prevalence of falls in people with rheumatoid arthritis. Annals of Physical & Rehabilitation Medicine. 2019;62(2):84-91.
