# Supplementary material for: Epidemiology and factors associated with osteoporosis, falls and fractures in patients with chronic inflammatory rheumatic disease: a scoping review
Source: BMJ Open. 2025 Jul 28;15(7):e096226. doi: 10.1136/bmjopen-2024-096226 (PMC12306236; doi:10.1136/bmjopen-2024-096226)
Supplement: online supplemental file 5 [file bmjopen-15-7-s005.pdf]

Appendix 5. References to studies exploring associated factors in CIRD

Table 1. References to studies exploring associated factors in RA

		Osteoporosis (84)	Fractures (90)	Falls (24)
**Demographics**	Age	51 (60.7) [1-49]	55 (61.1) [3, 6, 8, 9, 14, 20, 25, 27, 30, 35, 36, 41-44, 50-88]	18 (75.0) [69, 89-105]
	Gender	21 (25.0) [9-11, 13-15, 24, 27, 30, 33, 34, 38, 39, 42, 43, 46, 48, 106, 107]	26 (28.9) [9, 14, 27, 30, 42, 43, 56, 58, 60, 67-70, 72-75, 77, 78, 82, 83, 85-87, 108, 109]	11 (45.8) [69, 90, 93, 97-104]
	BMI	36 (42.9) [1, 3, 5, 6, 9, 10, 13-22, 25, 27-36, 38, 40-44, 46, 48, 49, 52]	36 (40.0) [3, 6, 9, 14, 20, 25, 27, 30, 35, 36, 41-44, 52-55, 57, 59-61, 63, 64, 67, 69-71, 74, 78, 80, 82-86]	8 (33.3) [69, 92-94, 97, 98, 102, 104]
	Ethnicity	0 (0.0)	3 (3.3) [82-84]	1 (4.2) [104]
	Menopausal status	23 (27.4) [3-5, 7-9, 12-14, 17, 18, 23, 25, 33-35, 38, 41, 43, 45, 47-49, 52]	19 (21.1) [3, 8, 9, 14, 25, 35, 41, 43, 52, 55, 63-65, 69, 71, 80, 81, 84, 88]	1 (4.2) [69]

	Smoking	14 (16.7) [2, 3, 9-11, 13-15, 18, 25, 30, 35, 36, 39, 41]	25 (27.8) [3, 9, 14, 25, 30, 35, 36, 41, 53-56, 60-63, 67, 69, 71, 74, 78, 81-83, 86]	2 (8.3) [69, 97]
	Alcohol	3 (3.6) [3, 13, 15]	7 (7.8) [3, 54, 62, 67, 74, 78, 83]	0 (0.0)
	Fracture history	9 (10.7)[8, 13, 14, 20, 22, 32, 35, 36, 44]	21 (23.3) [8, 14, 20, 35, 36, 44, 54, 60-62, 64, 65, 67, 71, 73, 74, 78, 81, 83, 86, 87]	2 (8.3) [93, 100]
	Falls history	2 (2.4) [30, 42]	3 (3.3) [30, 42, 86]	5 (20.8) [91, 97, 100, 101, 110]
	Physical activity	1 (1.2)[40]	3 (3.3) [54, 55, 63]	3 (12.5)[97, 99, 104]
	Marital status	0 (0.0)	1 (1.1) [55]	2 (8.3) [99, 104]
	Education attainment	0 (0.0)	2 (3.3) [55, 82]	1 (4.2) [104]
	Socioeconomic status	0 (0.0)	0 (0.0)	2 (8.3) [99, 104]
**Medications**	Disease-specific			
	Glucocorticoids	46 (54.8) [2-5, 8, 9, 11-15, 17, 18, 20-25, 27-30, 32-34, 36, 38, 40-45, 49, 52, 73, 108, 111-117]	59 (65.6) [3, 8, 9, 14, 20, 25, 27, 30, 36, 41-44, 50-57, 59-65, 67-74, 76, 78-80, 82-84, 87, 88, 109, 111, 115, 117-127]	9 (37.5) [69, 93, 97, 98, 100, 104, 105, 112, 115]

	csDMARDs*	12 (14.3) [3, 5, 18, 24, 28-30, 32, 33, 41, 108, 128]	21 (23.3) [3, 30, 41, 50, 51, 55, 60, 63-65, 67, 68, 71, 72, 78, 82, 122, 123, 127-129]	3 (12.5) [93, 97, 104]
	bDMARDs*	8 (9.5)[3, 13, 16, 18, 24, 30, 32, 108]	18 (20.0) [3, 30, 51, 55, 60, 63-65, 67, 68, 72, 74, 78, 82, 122, 123, 127, 129]	3 (12.5) [93, 97, 104]
	tsDMARDs*	0 (0.0)	2 (2.2) [64, 74]	0 (0.0)
	DMARDs, unspecified	6 (7.1)[2, 8, 11, 25, 27, 42]	7 (7.8) [8, 25, 27, 42, 53, 54, 86]	1 (4.2) [90]
Analgesics				
	Acetaminophen	0 (0.0)	1 (1.1)	0 (0.0)
	NSAIDs**	4 (4.8)[11, 24, 30, 108]	6 (6.7) [30, 67, 72, 82, 86, 130]	0 (0.0)
	Opioids	1 (1.2)[73]	5 (5.6) [73, 82, 127, 130, 131]	0 (0.0)
Osteoporosis-related				
	Anti-resorptive +/- anabolics	9 (10.7) [2, 3, 23, 25, 30, 35, 38, 44, 73]	21 (23.3) [3, 25, 30, 35, 44, 54, 55, 60, 62, 67, 71-73, 78, 80-82, 84, 85, 87, 127]	1 (4.2) [93]
	Calcium supplements	5 (6.0)[2, 3, 27, 38, 52]	6 (6.7) [3, 27, 52, 71, 80, 84]	0 (0.0)

	Vitamin D supplements	4 (4.8)[2, 3, 27, 38]	9 (10.0) [3, 27, 60, 62, 67, 71, 78, 80, 84]	1 (4.2) [93]
	HRT***	4 (4.8)[2, 3, 17, 25]	4 (4.4) [3, 25, 71, 127]	0 (0.0)
Other				
	Anti-hypertensives	0 (0.0)	0 (0.0)	2 (8.3) [94, 98]
	Diuretics	0 (0.0)	0 (0.0)	1 (4.2) [94]
	PPIs****	0 (0.0)	7 (7.8) [60, 67, 72, 78, 82, 118, 127]	0 (0.0)
	Anti-depressants	1 (1.2)[73]	4 (4.4) [72, 73, 82, 127]	2 (8.3) [98, 99]
	Psychotropics	0 (0.0)	2 (2.2) [82, 127]	2 (8.3) [100, 110]
	Anticoagulants	0 (0.0)	1 (1.1) [72]	1 (4.2) [110]
	Anti-convulsants	1 (1.2) [73]	3 (3.3) [73, 82, 127]	0 (0.0)
	Oral hypoglycemics	0 (0.0)	0 (0.0)	1 (4.2) [98]
	Thyroid hormone replacement	0 (0.0)	1 (1.1) [72]	0 (0.0)

**Disease-related**	Disease duration	39 (46.2) [1, 3, 4, 10-14, 16, 18-20, 22-25, 27-36, 38-45, 47, 49, 52, 112, 113, 116]	41 (45.6) [3, 14, 20, 25, 27, 30, 35, 36, 41-44, 50-54, 57, 59-65, 67-71, 74, 78, 80-82, 85-88, 127]	14 (58.3) [69, 89, 90, 92, 94-98, 101, 102, 104, 105, 112]
	Disease activity	34 (40.5)[1-4, 6, 8-11, 14, 17-21, 24, 25, 27, 28, 30, 32, 33, 35, 36, 38, 40, 41, 43-46, 48, 52, 112, 113]	37 (41.1) [3, 6, 8, 9, 14, 20, 25, 27, 30, 35, 36, 41, 43, 44, 52, 54, 56, 60-65, 67, 69-71, 74, 78, 80-82, 84, 85, 87, 88]	16 (66.7) [69, 89-92, 94-97, 100-102, 104, 105, 110, 112]
	Inflammatory markers	25 (29.8) [1, 4, 13, 14, 18, 20, 22, 23, 25, 27, 30, 32-34, 36, 38, 41, 43-46, 49, 112, 113, 116]	21 (23.3) [14, 20, 25, 27, 30, 36, 41, 43, 44, 54, 57, 60, 61, 64, 67, 73, 74, 78, 80, 87]	4 (16.7) [89, 94, 97, 112]
	Autoantibodies	35 (41.7) [1-4, 10, 12-14, 17-23, 25, 27, 30, 32-36, 41, 43-46, 48, 49, 107, 113, 132, 133]	27 (30.0) [3, 14, 20, 25, 27, 30, 35, 36, 41, 43, 44, 54, 57, 59, 60, 62, 64, 65, 67, 71, 74, 78, 80, 87, 88, 129, 133]	3 (12.5) [89, 97, 104]
	Radiographic damage	8 (9.5) [11, 13, 18, 21, 25, 30, 43, 116]	6 (6.7) [25, 30, 43, 57, 64, 134]	0 (0.0)
**Other**	Vitamin D level	7 (8.3) [1, 16, 34, 41, 48, 52, 135]	4 (4.4) [41, 52, 83, 136]	1 (4.2) [97]
	Bone turnover markers	3 (3.6) [32-34]	1 (1.1) [80]	0 (0.0)

	Mobility aids	1 (1.2) [30]	1 (1.1) [30]	3 (12.5) [91, 95, 110]
	Co-morbidities	1 (1.2) [13]	6 (6.7) [72, 82, 83, 86, 122, 127]	3 (12.5) [90, 99, 100]
	Mobility/balance tests	0 (0.0)	1 (1.1) [137]	11 (45.8)[89, 91, 95, 96, 99-102, 104, 110, 137]

*csDMARD = conventional synthetic DMARD, bDMARD = biologic DMARD, tsDMARD = targeted synthetic DMARD

**NSAID = non-steroidal anti-inflammatory drug

***HRT = hormone replacement therapy

****PPI = proton pump inhibitor

Table 2. References to studies exploring associated factors in PsA

		Osteoporosis (13)	Fractures (9)	Falls (2)
**Demographics**	Age	4 (30.8) [138-141]	5 (55.6) [138, 141-144]	1 (50.0) [144]
	Gender	3 (23.1) [141, 145, 146]	3 (33.3) [141, 142, 146]	0 (0.0)
	BMI	3 (23.1) [138, 139, 141]	3 (33.3) [138, 141, 144]	2 (100.0) [144, 147]
	Ethnicity	1 (7.7) [148]	0 (0.0)	0 (0.0)
	Menopausal status	4 (30.8) [138, 139, 141, 149]	3 (33.3) [138, 141, 144]	1 (50.0) [144]
	Smoking	1 (7.7) [141]	2 (22.2) [141, 142]	0 (0.0)
**Medications**	Disease specific			N/A
	Glucocorticoids	2 (15.4) [140, 141]	2 (15.4) [141, 142]	
	csDMARDs	1 (7.7)	1 (11.1) [142]	
	bDMARDs	1 (7.7)	1 (11.1) [142]	
	NSAIDs	1 (7.7)	1 (11.1) [142]	
**Disease-related**	Disease duration	5 (38.5) [139-141, 145, 150]	4 (44.4) [141-144]	1 (50.0) [144]
	Disease activity	4 (30.8) [138, 140, 141, 150]	4 (44.4) [138, 141, 143, 144]	1 (50.0) [144]
	Inflammatory markers	5 (38.5) [138-141, 150]	3 (33.3) [138, 141, 143]	0 (0.0)

	Radiographic damage	1 (7.7) [139]	1 (11.1) [143]	0 (0.0)
	Disease manifestations	2 (15.4) [140, 141]	3 (33.3) [141, 142, 151]	0 (0.0)
**Other**	Vitamin D level	1 (7.7) [145]	0 (0.0)	0 (0.0)
	Bone turnover markers	1 (7.7) [150]	0 (0.0)	0 (0.0)
	Co-morbidities	0 (0.0)	0 (0.0)	1 (50.0) [147]

Table 3. References to studies exploring associated factors in AS

		Osteoporosis (29)	Fractures (32)	Falls (1)
**Demographics**	Age	16 (55.2) [152-165]	17 (53.1) [143, 152, 155, 158, 160, 166-177]	1 (100.0) [178]
	Gender	14 (48.3) [152-159, 162, 163, 179-182]	13 (40.6) [152, 155, 158, 168, 170-172, 174, 176, 180-183]	0 (0.0)
	BMI	7 (24.1) [155, 158, 159, 162-164, 177]	8 (25.0) [155, 158, 167, 169, 171, 172, 175, 177]	0 (0.0)
	Ethnicity	1 (3.4) [157]	1 (3.1) [158]	0 (0.0)
	Menopausal status	4 (13.8) [155, 156, 158, 180]	3 (9.4) [155, 158, 180]	0 (0.0)
	Smoking	4 (13.8) [155, 157, 158, 181]	5 (15.6) [155, 158, 169, 172, 181]	0 (0.0)
	Alcohol	1 (3.4) [158]	2 (6.3) [158, 169]	0 (0.0)
	Physical activity	3 (10.3) [155, 156, 158]	2 (6.3) [155, 158]	0 (0.0)
	Fracture history	2 (6.9) [156, 158]	3 (9.4) [158, 168, 171]	0 (0.0)
	Falls history	1 (3.4) [158]	1 (3.1) [158]	0 (0.0)
	Marital status	1 (3.4) [158]	1 (3.1) [158]	0 (0.0)
	Education attainment	1 (3.4) [158]	1 (3.1) [158]	0 (0.0)

	Socioeconomic status	2 (6.9) [153, 158]	1 (3.1) [158]	0 (0.0)
**Medications**	Disease specific			
	Glucocorticoids	4 (13.8) [155, 156, 163, 165]	5 (15.6) [155, 170-172, 176]	0 (0.0)
	csDMARDs	2 (6.9) [155, 156]	4 (12.5) [155, 168, 170, 176]	0 (0.0)
	bDMARDs	8 (27.6) [155-157, 159, 160, 180, 184, 185]	6 (18.8) [155, 160, 168, 170, 180, 184]	0 (0.0)
	DMARDs unspecified	0 (0.0)	2 (6.3) [171, 172]	0 (0.0)
	NSAIDs	3 (10.3) [155, 156, 159]	6 (18.8) [155, 168, 170, 176, 186, 187]	0 (0.0)
	Osteoporosis-related			
	Anti-resorptive +/- anabolics	2 (6.9) [156, 180]	4 (12.5) [168, 171, 172, 180]	0 (0.0)
	Calcium supplements	1 (3.4) [156]	2 (6.3) [171, 172]	0 (0.0)
	Vitamin D supplements	1 (3.4) [156]	2 (6.3) [171, 172]	0 (0.0)
	HRT	1 (3.4) [156]	0 (0.0) [176]	0 (0.0)

	Psychotropics	0 (0.0)	1 (3.1)	0 (0.0)
**Disease-related**	Disease duration	12 (41.4) [154-157, 159, 161-163, 165, 188]	14 (43.8) [143, 155, 166-168, 170-175, 177, 187, 188]	1 (100.0) [178]
	Disease activity	18 (62.1) [152, 154-162, 164, 165, 180, 188-191]	14 (43.8) [143, 152, 155, 158, 160, 169, 171, 172, 174, 175, 177, 180, 188, 189]	1 (100.0) [178]
	Inflammatory markers	19 (65.5) [152, 154-164, 180, 188, 190-192]	14 (43.8) [143, 152, 155, 158, 160, 167, 168, 170, 172, 175, 177, 180, 188, 192]	1 (100.0) [178]
	HLA-B27 positivity	5 (17.2) [155-157, 159, 164]	5 (15.6) [155, 166, 168, 171, 172]	0 (0.0)
	Radiographic damage	11 (37.9) [154, 156, 159-163, 180, 189]	12 (37.5) [143, 155, 160, 167, 168, 171, 172, 174, 175, 180, 189]	0 (0.0)
	Spinal mobility	7 (24.1) [155, 156, 158, 159, 164, 189, 191]	6 (18.8) [155, 158, 171, 172, 174, 189]	0 (0.0)
	Disease manifestations	6 (20.7) [155, 156, 159, 163, 180, 191]	6 (18.8) [155, 166, 168, 170, 172, 180]	0 (0.0)

**Other**	Vitamin D level	5 (17.2) [152, 154, 157, 158, 192]	5 (15.6) [152, 158, 171, 172, 192]	0 (0.0)
	Bone turnover markers	7 (24.1) [154, 157, 158, 164, 189, 191, 193]	4 (12.5) [158, 169, 189, 192]	0 (0.0)
	Co-morbidities	1 (3.4) [153]	1 (3.1) [176]	0 (0.0)

Table 4. References to studies exploring associated factors in SLE

		Osteoporosis (38)	Fractures (39)
**Demographics**	Age	26 (68.4) [9, 15, 194-217]	28 (71.8) [9, 84, 194, 197, 199, 201, 203, 205-207, 209, 213, 217-232]
	Gender	9 (23.7) [9, 15, 177, 201, 203, 204, 210, 212, 214]	6 (15.4)[9, 201, 203, 205, 220, 225]
	BMI	16 (42.1) [9, 15, 194, 195, 197-199, 202, 203, 205, 206, 208, 209, 214, 215, 233]	17 (43.6) [9, 84, 194, 197, 199, 203, 205, 206, 209, 213, 218, 221, 222, 224, 227-229, 233]
	Ethnicity	10 (26.3) [199, 201, 203-205, 213, 214, 216, 217, 234]	8 (20.5) [84, 201, 203, 205, 213, 217, 218, 220]
	Menopausal status	15 (39.5)[9, 197, 199, 200, 202, 204, 208-211, 214, 217, 233-235]	14 (35.9) [9, 84, 197, 199, 209, 217, 218, 223, 224, 227, 229, 232, 233, 235]
	Smoking	15 (39.5) [9, 15, 200-203, 205, 206, 209-211, 213, 216, 234, 236]	15 (38.5) [9, 28, 201, 203, 205, 206, 218, 221-223, 227-229, 236]
	Alcohol	6 (15.8) [15, 200, 202, 205, 236, 237]	8 (20.5)[9, 203, 205, 221-223, 227, 236]
	Physical activity	8 (21.1) [200, 202, 204, 209, 214, 216, 234, 237]	4 (10.3) [209, 218, 222, 223]
	Fracture history	3 (7.9) [199, 200, 209]	5 (12.8) [219, 223, 226, 229, 232]
	Family history of OP	3 (7.9) [214, 237, 238]	3 (7.7)[218, 223, 226]
	Falls history	1 (2.6) [202]	0 (0.0)

**Medications**	Disease specific			
		Glucocorticoids	29 (76.3)[9, 15, 194-206, 208-211, 213, 214, 216, 233, 234, 236, 237, 239-241]	30 (76.9) [9, 60, 84, 124, 194, 197, 199, 201, 203, 205, 206, 209, 213, 218-222, 224, 226, 228, 229, 231-233, 236, 240, 242-244]
		Anti-malarials	11 (28.9) [194, 201, 204, 206, 208-211, 213, 234, 245]	13 (33.3) [194, 201, 206, 209, 213, 218, 222, 223, 228, 231, 232, 242, 245]
		Immunosuppressants	12 (31.6) [194, 195, 200-202, 204, 206, 208-211, 213, 245]	10 (25.6) [194, 201, 206, 209, 213, 223, 231, 232, 242, 245]
	Osteoporosis-related			
		Anti-resorptive +/- anabolics	10 (26.3) [194, 201-204, 208-211, 233]	11 (28.2) [84, 194, 201, 203, 209, 223, 224, 227, 229, 233, 242]
		Calcium supplements	10 (26.3) [194, 200, 201, 203, 204, 210, 211, 214, 233, 241]	10 (25.6) [84, 194, 201, 203, 227-229, 232, 233, 242]
		Vitamin D supplements	11 (28.9)[194, 195, 200, 201, 203, 204, 210, 211, 213, 214, 233]	10 (25.6) [84, 194, 201, 203, 213, 227, 228, 232, 233, 242]
		HRT	5 (13.2) [202, 206, 209, 211, 245]	6 (15.4) [206, 209, 223, 227, 242, 245]
	Other			
		Diuretics	2 (5.3) [205, 214]	2 (5.1) [205, 227]
		PPIs	1 (2.6)_[205]	1 (2.6) [205]
		Anti-depressants	1 (2.6)_[205]	1 (2.6) [205]

	Psychotropics	1 (2.6)_[202]	1 (2.6) [223]
	Anticoagulants	2 (5.3)[199, 202]	3 (7.7) [199, 223, 231]
**Disease-related**	Disease duration	17 (44.7) [194, 196, 198-200, 203, 206, 209, 211, 213, 215-217, 233, 234, 237, 241]	15 (38.5) [23, 194, 199, 203, 206, 209, 213, 217-220, 222, 223, 227, 232]
	Disease activity	19 (50.0) [9, 195, 196, 198-202, 204, 206, 209, 211, 213, 215, 216, 233, 234, 237, 241]	14 (35.9) [9, 84, 199, 201, 206, 209, 213, 218, 222, 223, 226, 227, 232, 236]
	Damage accrual	16 (42.1) [9, 195, 196, 198-201, 204, 206, 208-211, 213, 217, 233]	13 (33.3) [9, 199, 201, 206, 209, 213, 217, 218, 222, 227, 228, 232, 233]
	Inflammatory markers	7 (18.4) [194, 196, 200, 202, 206, 213, 215]	4 (10.3) [194, 206, 213, 226]
	Autoantibodies	2 (5.3) [236, 245]	3 (7.7) [227, 236, 245]
	Disease manifestations	3 (7.9) [201, 236, 245]	6 (15.4) [201, 227, 230, 232, 236, 245]
**Other**	Vitamin D level	8 (21.1) [197, 199, 200, 202, 204, 208, 213, 215]	3 (7.7) [199, 213, 218]
	Bone turnover markers	5 (13.2) [195, 198, 202, 206, 215]	1 (2.6) [206]
	Co-morbidities	6 (15.8) [202, 203, 205, 210, 211, 214]	7 (17.9) [203, 205, 220, 222, 223, 225, 231]

References

1. Arain SR, Riaz A, Nazir L, Umer TP, Rasool T. Low Bone Mineral Density among Patients with Newly Diagnosed Rheumatoid Arthritis. J Ayub Med Coll Abbottabad. 2016;28(1):175-8.
2. Book C, Karlsson M, Akesson K, Jacobsson L. Disease activity and disability but probably not glucocorticoid treatment predicts loss in bone mineral density in women with early rheumatoid arthritis. Scandinavian Journal of Rheumatology. 2008;37(4):248-54.
3. Breban S, Briot K, Kolta S, Paternotte S, Ghazi M, Fechtenbaum J, et al. Identification of rheumatoid arthritis patients with vertebral fractures using bone mineral density and trabecular bone score. Journal of Clinical Densitometry. 2012;15(3):260-6.
4. Dao HH, Do QT, Sakamoto J. Bone mineral density and frequency of osteoporosis among Vietnamese women with early rheumatoid arthritis. Clinical Rheumatology. 2011;30(10):1353-61.
5. di Munno O, Mazzantini M, Sinigaglia L, Bianchi G, Minisola G, Muratore M, et al. Effect of low dose methotrexate on bone density in women with rheumatoid arthritis: results from a multicenter cross-sectional study. Journal of Rheumatology. 2004;31(7):1305-9.
6. El Maghraoui A, Sadni S, Rezqi A, Bezza A, Achemlal L, Mounach A. Does Rheumatoid Cachexia Predispose Patients with Rheumatoid Arthritis to Osteoporosis and Vertebral Fractures? Journal of Rheumatology. 2015;42(9):1556-62.
7. Gabdulina G, Kasher M, Beissebayeva A, Mussabaeva D, Tokarev A, Mominova G, et al. An epidemiological analysis of osteoporotic characteristics in patients affected with rheumatoid arthritis in Kazakhstan. Archives of Osteoporosis. 2018;13(1):99.
8. Ghazi M, Kolta S, Briot K, Fechtenbaum J, Paternotte S, Roux C. Prevalence of vertebral fractures in patients with rheumatoid arthritis: revisiting the role of glucocorticoids. Osteoporosis International. 2012;23(2):581-7.
9. Gilboe IM, Kvien TK, Haugeberg G, Husby G. Bone mineral density in systemic lupus erythematosus: comparison with rheumatoid arthritis and healthy controls. Annals of the Rheumatic Diseases. 2000;59(2):110-5.
10. Guler-Yuksel M, Bijsterbosch J, Goekoop-Ruiterman YP, de Vries-Bouwstra JK, Ronday HK, Peeters AJ, et al. Bone mineral density in patients with recently diagnosed, active rheumatoid arthritis. Annals of the Rheumatic Diseases. 2007;66(11):1508-12.
11. Hafez EA, Mansour HE, Hamza SH, Moftah SG, Younes TB, Ismail MA. Bone Mineral Density Changes in Patients with Recent-Onset Rheumatoid Arthritis. Clinical Medicine Insights: Arthritis & Musculoskeletal Disorders. 2011(4):87-94.
12. Haugeberg G, Uhlig T, Falch JA, Halse JI, Kvien TK. Bone mineral density and frequency of osteoporosis in female patients with rheumatoid arthritis: results from 394 patients in the Oslo County Rheumatoid Arthritis register. Arthritis & Rheumatism. 2000;43(3):522-30.
13. Hauser B, Riches PL, Wilson JF, Horne AE, Ralston SH. Prevalence and clinical prediction of osteoporosis in a contemporary cohort of patients with rheumatoid arthritis. Rheumatology. 2014;53(10):1759-66.
14. Huang H, Wang Y, Xie W, Geng Y, Gao D, Zhang Z. Impact of Treat-to-Target Therapy on Bone Mineral Density Loss in Patients With Rheumatoid Arthritis: A Prospective Cohort Study. Frontiers in Endocrinology. 2022;13:867610.
15. Hu Z, Xu S, Lin H, Ni W, Yang Q, Qi J, et al. Prevalence and risk factors for bone loss in Southern Chinese with rheumatic diseases. BMC Musculoskeletal Disorders. 2020;21(1) (no pagination).
16. Hu Z, Zhang L, Lin Z, Zhao C, Xu S, Lin H, et al. Prevalence and risk factors for bone loss in rheumatoid arthritis patients from South China: modeled by three methods. BMC Musculoskeletal Disorders. 2021;22(1) (no pagination).

17. Hwang J, Lee EK, Ahn J, Cha HS, Koh EM, Lee J. Bone-density testing interval and transition to osteoporosis in patients with rheumatoid arthritis. Osteoporosis International. 2017;28(1):231-7.
18. Ileana MC, Nelutu MA. Relationship Between Physical Disability, Disease Activity and Osteoporosis in Patients With Rheumatoid Arthritis. Archives of Rheumatology. 2014;29(4):273-9.
19. Klodzinski L, Wislowska M. Comorbidities in rheumatic arthritis. Reumatologia. 2018;56(4):228-33.
20. Kvien TK, Haugeberg G, Uhlig T, Falch JA, Halse JI, Lems WF, et al. Data driven attempt to create a clinical algorithm for identification of women with rheumatoid arthritis at high risk of osteoporosis. Annals of the Rheumatic Diseases. 2000;59(10):805-11.
21. Kweon SM, Sohn DH, Park JH, Koh JH, Park EK, Lee HN, et al. Male patients with rheumatoid arthritis have an increased risk of osteoporosis: Frequency and risk factors. Medicine (United States). 2018;97(24) (no pagination).
22. Lee JH, Sung YK, Choi CB, Cho SK, Bang SY, Choe JY, et al. The frequency of and risk factors for osteoporosis in Korean patients with rheumatoid arthritis. BMC Musculoskeletal Disorders. 2016;17(1) (no pagination).
23. Lee SG, Park YE, Park SH, Kim TK, Choi HJ, Lee SJ, et al. Increased frequency of osteoporosis and BMD below the expected range for age among South Korean women with rheumatoid arthritis. International Journal of Rheumatic Diseases. 2012;15(3):289-96.
24. Lindner L, Callhoff J, Alten R, Krause A, Ochs W, Zink A, et al. Osteoporosis in patients with rheumatoid arthritis: trends in the German National Database 2007-2017. Rheumatology International. 2020;40(12):2005-12.
25. Lodder MC, Haugeberg G, Lems WF, Uhlig T, Orstavik RE, Kostense PJ, et al. Radiographic damage associated with low bone mineral density and vertebral deformities in rheumatoid arthritis: the Oslo-Truro-Amsterdam (OSTRA) collaborative study. Arthritis & Rheumatism. 2003;49(2):209-15.
26. Luque Ramos A, Redeker I, Hoffmann F, Callhoff J, Zink A, Albrecht K. Comorbidities in Patients with Rheumatoid Arthritis and Their Association with Patient-reported Outcomes: Results of Claims Data Linked to Questionnaire Survey. Journal of Rheumatology. 2019;46(6):564-71.
27. Ma CC, Xu SQ, Gong X, Wu Y, Qi S, Liu W, et al. Prevalence and risk factors associated with glucocorticoid-induced osteoporosis in Chinese patients with rheumatoid arthritis. Archives of Osteoporosis. 2017;12(1):33.
28. Manrique F, Gamardo J, De Elguezabal K, Martinis R, Castro JS, Bellorin-Font E, et al. Abnormalities of bone mineral density and bone metabolism in Venezuelan patients with rheumatoid arthritis. Journal of Clinical Rheumatology. 2003;9(4):219-27.
29. Mazzantini M, Di Munno O, Sinigaglia L, Bianchi G, Rossini M, Mela Q, et al. Effect of cyclosporine A on bone density in female rheumatoid arthritis patients: results from a multicenter, cross-sectional study. Clin Exp Rheumatol. 2007;25(5):709-15.
30. Mohammad A, Lohan D, Bergin D, Mooney S, Newell J, O'Donnell M, et al. The prevalence of vertebral fracture on vertebral fracture assessment imaging in a large cohort of patients with rheumatoid arthritis. Rheumatology. 2014;53(5):821-7.
31. Momohara S, Okamoto H, Yago T, Furuya T, Nanke Y, Kotake S, et al. The study of bone mineral density and bone turnover markers in postmenopausal women with active rheumatoid arthritis. Modern Rheumatology. 2005;15(6):410-4.
32. Nagayama Y, Ebina K, Tsuboi H, Hirao M, Hashimoto J, Yoshikawa H, et al. Low serum albumin concentration is associated with increased risk of osteoporosis in postmenopausal patients with rheumatoid arthritis. Journal of Orthopaedic Science. 2022;27(6):1283-90.

33. Nava-Valdivia CA, Ponce-Guarneros JM, Saldana-Cruz AM, Corona-Sanchez EG, Ramirez-Villafana M, Perez-Guerrero EE, et al. Assessment of Serum sRANKL, sRANKL/OPG Ratio, and Other Bone Turnover Markers with the Estimated 10-Year Risk of Major and Hip Osteoporotic Fractures in Rheumatoid Arthritis: A Cross-Sectional Study. BioMed Research International. 2021;2021:5567666.
34. Oelzner P, Schwabe A, Lehmann G, Eidner T, Franke S, Wolf G, et al. Significance of risk factors for osteoporosis is dependent on gender and menopause in rheumatoid arthritis. Rheumatology International. 2008;28(11):1143-50.
35. Orstavik RE, Haugeberg G, Uhlig T, Mowinckel P, Falch JA, Halse JI, et al. Self reported non-vertebral fractures in rheumatoid arthritis and population based controls: incidence and relationship with bone mineral density and clinical variables. Annals of the Rheumatic Diseases. 2004;63(2):177-82.
36. Orstavik RE, Haugeberg G, Uhlig T, Falch JA, Halse JI, Hoiseth A, et al. Vertebral deformities in 229 female patients with rheumatoid arthritis: associations with clinical variables and bone mineral density. Arthritis & Rheumatism. 2003;49(3):355-60.
37. Osiri M, Sattayasomboon Y. Prevalence and out-patient medical costs of comorbid conditions in patients with rheumatoid arthritis. Joint Bone Spine. 2013;80(6):608-12.
38. Peng J, Gong Y, Zhang Y, Xiao Z, Zeng Q, Chen S. Bone Mineral Density in Patients With Rheumatoid Arthritis and 4-Year Follow-up Results. JCR: Journal of Clinical Rheumatology. 2016;22(2):71-4.
39. Rexhepi S, Rexhepi M, Rexhepi B, Sahatciu-Meka V, Mahmutaj V. Bone Mineral Density in Patients With Early- Onset Rheumatoid Arthritis...2017 IOF-ESCEO World Congress on Osteoporosis. Medical Journal of Bakirkoy. 2020;16(1):44-8.
40. Sarkis KS, Salvador MB, Pinheiro MM, Silva RG, Zerbini CA, Martini LA. Association between osteoporosis and rheumatoid arthritisin women: A cross-sectional study. Sao Paulo Medical Journal. 2009;127(4):216-22.
41. Senosi MR, Fathi HM, Baki NMA, Zaki O, Magdy AM, Gheita TA. Bone mineral density, vitamin D receptor (VDR) gene polymorphisms, fracture risk assessment (FRAX), and trabecular bone score (TBS) in rheumatoid arthritis patients: connecting pieces of the puzzle. Clinical Rheumatology. 2022;41(5):1333-42.
42. Tong JJ, Xu SQ, Wang JX, Zong HX, Chu YR, Chen KM, et al. Interactive effect of sarcopenia and falls on vertebral osteoporotic fracture in patients with rheumatoid arthritis. Archives of Osteoporosis. 2021;16(1):145.
43. Tong JJ, Xu SQ, Zong HX, Pan MJ, Teng YZ, Xu JH. Prevalence and risk factors associated with vertebral osteoporotic fractures in patients with rheumatoid arthritis. Clinical Rheumatology. 2020;39(2):357-64.
44. Vis M, Haavardsholm EA, Boyesen P, Haugeberg G, Uhlig T, Hoff M, et al. High incidence of vertebral and non-vertebral fractures in the OSTRA cohort study: a 5-year follow-up study in postmenopausal women with rheumatoid arthritis. Osteoporosis International. 2011;22(9):2413-9.
45. Zahid PM, Samreen S, Khan ZA, Salim B, Ahmed SN, Gul H. Factors associated with low bone mineral density in postmenopausal women with rheumatoid arthritis. Journal of the Pakistan Medical Association. 2022;72(5):891-5.
46. Zeng T, Tan L, Yu J, Wu Y. High density lipoprotein in rheumatoid arthritis: emerging role in predicting inflammation level and osteoporosis occurrence. Scandinavian Journal of Clinical & Laboratory Investigation. 2020;80(5):375-80.
47. Aghaei M, Sedighi S, Behnampour N, Hezarkhani S, Shirashiani M, Mohebi EG, et al. Change in bone mineral density in post menopausal women with rheumatoid arthritis. Bangladesh Journal of Medical Science. 2013;12(2):158-63.

48. Ketabforoush AHME, Aleahmad M, Qorbani M, Mehrpoor G, Afrashteh S, Mardi S, et al. Bone mineral density status in patients with recent-onset rheumatoid arthritis. Journal of Diabetes & Metabolic Disorders. 2023;22(1):775-85.
49. Wafa H, Raja A, Dhia K, Nada B, Imene Z, Montacer KM. Risk factors associated with bone loss and occurrence of fragility fractures in rheumatoid arthritis patients. The Egyptian Rheumatologist. 2019;41(1):1-5.
50. Arai K, Hanyu T, Sugitani H, Murai T, Fujisawa J, Nakazono K, et al. Risk factors for vertebral fracture in menopausal or postmenopausal Japanese women with rheumatoid arthritis: A cross-sectional and longitudinal study. Journal of Bone and Mineral Metabolism. 2006;24(2):118-24.
51. Arai K, Suzuki N, Murayama T, Kondo N, Otsuka H, Koizumi M, et al. Age at the time of hip fracture in patients with rheumatoid arthritis is 4 years greater than it was 10 years before, but is still younger than that of the general population. Modern Rheumatology. 2020;30(1):64-9.
52. Avouac J, Koumakis E, Toth E, Meunier M, Maury E, Kahan A, et al. Increased risk of osteoporosis and fracture in women with systemic sclerosis: a comparative study with rheumatoid arthritis. Arthritis care & research. 2012;64(12):1871-8.
53. Baskan BM, Sivas F, Alemdaroglu E, Duran S, Ozoran K. Association of bone mineral density and vertebral deformity in patients with rheumatoid arthritis. Rheumatology International. 2007;27(6):579-84.
54. Cho SK, Lee JH, Han M, Lee S, Kim JY, Ryu JA, et al. The influence of vertebral fracture on the functional disability of patients with rheumatoid arthritis. Journal of Korean Medical Science. 2014;29(6):859-63.
55. Coulson KA, Reed G, Gilliam BE, Kremer JM, Pepmueller PH. Factors influencing fracture risk, T score, and management of osteoporosis in patients with rheumatoid arthritis in the Consortium of Rheumatology Researchers of North America (CORRONA) registry. JCR: Journal of Clinical Rheumatology. 2009;15(4):155-60.
56. Dirven L, Van Den Broek M, Van Groenendael JHLM, De Beus WM, Kerstens PJSM, Huizinga TWJ, et al. Prevalence of vertebral fractures in a disease activity steered cohort of patients with early active rheumatoid arthritis. BMC Musculoskeletal Disorders. 2012;13 (no pagination).
57. El Maghraoui A, Rezqi A, Mounach A, Achemlal L, Bezza A, Ghozlani I. Prevalence and risk factors of vertebral fractures in women with rheumatoid arthritis using vertebral fracture assessment. Rheumatology. 2010;49(7):1303-10.
58. Erwin J, Enki DG, Woolf AD. Younger people with rheumatoid arthritis are at increased risk of fracture even before age 50 years: a population-based cohort study. Osteoporosis International. 2021;32(8):1651-9.
59. Filho JC, Pinheiro MM, de Moura Castro CH, Szejnfeld VL. Prevalence and risk factors associated with low-impact fractures in men with rheumatoid arthritis. Clinical Rheumatology. 2014;33(10):1389-95.
60. Furuya T, Inoue E, Hosoi T, Taniguchi A, Momohara S, Yamanaka H. Risk factors associated with the occurrence of hip fracture in Japanese patients with rheumatoid arthritis: a prospective observational cohort study. Osteoporosis International. 2013;24(4):1257-65.
61. Furuya T, Kotake S, Inoue E, Nanke Y, Yago T, Hara M, et al. Risk factors associated with incident fractures in Japanese men with rheumatoid arthritis: A prospective observational cohort study. Journal of Bone and Mineral Metabolism. 2008;26(5):499-505.
62. Furuya T, Kotake S, Inoue E, Nanke Y, Yago T, Kobashigawa T, et al. Risk factors associated with incident clinical vertebral and nonvertebral fractures in Japanese women with rheumatoid arthritis: a prospective 54-month observational study. Journal of Rheumatology. 2007;34(2):303-10.

63. Gamez-Nava JI, Ramirez-Villafana M, Cons-Molina F, Gomez-Ramirez EE, Esparza-Guerrero Y, Saldana-Cruz AM, et al. Serum irisin concentrations and osteoporotic vertebral fractures in women with rheumatoid arthritis: A cross-sectional study. Medicine. 2022;101(6):e28799.
64. Gomez-Vaquero C, Hernandez JL, Olmos JM, Cerda D, Calleja CH, Lopez JAM, et al. High incidence of clinical fragility fractures in postmenopausal women with rheumatoid arthritis. A case-control study. Bone. 2023;168:116654.
65. Guanabens N, Olmos JM, Hernandez JL, Cerda D, Hidalgo Calleja C, Martinez Lopez JA, et al. Vertebral fractures are increased in rheumatoid arthritis despite recent therapeutic advances: a case-control study. Osteoporosis International. 2021;32(7):1333-42.
66. Huusko TM, Korpela M, Karppi P, Avikainen V, Kautiainen H, Sulkava R. Threefold increased risk of hip fractures with rheumatoid arthritis in Central Finland. Annals of the Rheumatic Diseases. 2001;60(5):521-2.
67. Ishida O, Furuya T, Inoue E, Ochi K, Ikari K, Taniguchi A, et al. Risk factors for established vertebral fractures in Japanese patients with rheumatoid arthritis: Results from a large prospective observational cohort study. Modern Rheumatology. 2015;25(3):373-8.
68. Jin S, Li M, Fang Y, Li Q, Liu J, Duan X, et al. Chinese Registry of rheumatoid arthritis (CREDIT): II. prevalence and risk factors of major comorbidities in Chinese patients with rheumatoid arthritis. Arthritis research & therapy. 2017;19(1):251.
69. Jin S, Li M, Wang Q, Zeng X, Xia W, Yu W, et al. Bone mineral density and microarchitecture among Chinese patients with rheumatoid arthritis: a cross-sectional study with HRpQCT. Arthritis Research and Therapy. 2021;23(1) (no pagination).
70. Kim D, Cho SK, Choi CB, Jun JB, Kim TH, Lee HS, et al. Incidence and risk factors of fractures in patients with rheumatoid arthritis: an Asian prospective cohort study. Rheumatology International. 2016;36(9):1205-14.
71. Kim D, Cho SK, Kim JY, Choi YY, Sung YK. Association between trabecular bone score and risk factors for fractures in Korean female patients with rheumatoid arthritis. Modern Rheumatology. 2016;26(4):540-5.
72. Kim D, Cho SK, Park B, Jang EJ, Bae SC, Sung YK. Glucocorticoids Are Associated with an Increased Risk for Vertebral Fracture in Patients with Rheumatoid Arthritis. Journal of Rheumatology. 2018;45(5):612-20.
73. Kim SY, Schneeweiss S, Liu J, Daniel GW, Chang CL, Garneau K, et al. Risk of osteoporotic fracture in a large population-based cohort of patients with rheumatoid arthritis. Arthritis research & therapy. 2010;12(4):R154.
74. Lin PH, Yu SF, Chen JF, Chen YC, Lai HM, Chiu WC, et al. Risk factor analysis of fragility fractures in rheumatoid arthritis: A 3-year longitudinal, real-world, observational, cohort study. PLoS ONE [Electronic Resource]. 2021;16(8):e0255542.
75. Lin YC, Li YH, Chang CH, Hu CC, Chen DW, Hsieh PH, et al. Rheumatoid arthritis patients with hip fracture: a nationwide study. Osteoporosis International. 2015;26(2):811-7.
76. Nampei A, Hashimoto J, Koyanagi J, Ono T, Hashimoto H, Tsumaki N, et al. Characteristics of fracture and related factors in patients with rheumatoid arthritis. Modern Rheumatology. 2008;18(2):170-6.
77. Ochi K, Furuya T, Ikari K, Taniguchi A, Yamanaka H, Momohara S. Sites, frequencies, and causes of self-reported fractures in 9,720 rheumatoid arthritis patients: a large prospective observational cohort study in Japan. Archives of Osteoporosis. 2013;8:130.
78. Ochi K, Go Y, Furuya T, Ikari K, Taniguchi A, Yamanaka H, et al. Risk factors associated with the occurrence of distal radius fractures in Japanese patients with rheumatoid arthritis: a prospective observational cohort study. Clinical Rheumatology. 2014;33(4):477-83.
79. Okano T, Inui K, Tada M, Sugioka Y, Mamoto K, Wakitani S, et al. High frequency of vertebral fracture and low bone quality in patients with rheumatoid arthritis-Results from TOMORROW study. Modern Rheumatology. 2017;27(3):398-404.

80. Omata Y, Hagiwara F, Nishino J, Matsudaira K, Kadono Y, Juji T, et al. Vertebral fractures affect functional status in postmenopausal rheumatoid arthritis patients. Journal of Bone and Mineral Metabolism. 2014;32(6):725-31.
81. Orstavik RE, Haugeberg G, Uhlig T, Mowinckel P, Falch JA, Halse JI, et al. Incidence of vertebral deformities in 255 female rheumatoid arthritis patients measured by morphometric X-ray absorptiometry. Osteoporosis International. 2005;16(1):35-42.
82. Ozen G, Pedro S, Wolfe F, Michaud K. Medications associated with fracture risk in patients with rheumatoid arthritis. Annals of the Rheumatic Diseases. 2019;78(8):1041-7.
83. Peterkin-McCalman R, Waller JL, Le B, Oliver AM, Manning E, Elam RE, et al. Fractures in patients with rheumatoid arthritis and end-stage renal disease. Archives of Osteoporosis. 2020;15(1):146.
84. Rentero ML, Amigo E, Chozas N, Fernandez Prada M, Silva-Fernandez L, Abad Hernandez MA, et al. Prevalence of fractures in women with rheumatoid arthritis and/or systemic lupus erythematosus on chronic glucocorticoid therapy. BMC Musculoskeletal Disorders. 2015;16:300.
85. Suzuki A, Tamai K, Takahashi S, Yamada K, Inui K, Tada M, et al. Do rheumatoid arthritis patients have low back pain or radiological lumbar lesions more frequently than the healthy population? - Cross-sectional analysis in a cohort study with age and sex-matched healthy volunteers. Spine Journal. 2020;20(12):1995-2002.
86. van Staa TP, Geusens P, Bijlsma JW, Leufkens HG, Cooper C. Clinical assessment of the long-term risk of fracture in patients with rheumatoid arthritis. Arthritis & Rheumatism. 2006;54(10):3104-12.
87. Yoshii I, Sawada N, Chijiwa T, Kokei S. Impact of sustaining SDAI remission for preventing incident of bone fragility fracture in patient with rheumatoid arthritis. Annals of the Rheumatic Diseases. 2022;81(2):296-9.
88. Majjad A, Ghassem MA, Toufik H, Sadni S, Debbarh Z, Djossou JH, et al. Relationship between vertebral fracture prevalence and abdominal aortic calcification in women with rheumatoid arthritis. Bone. 2020;141:115599.
89. Bohler C, Radner H, Ernst M, Binder A, Stamm T, Aletaha D, et al. Rheumatoid arthritis and falls: the influence of disease activity. Rheumatology. 2012;51(11):2051-7.
90. Brenton-Rule A, Dalbeth N, Menz HB, Bassett S, Rome K. Foot and ankle characteristics associated with falls in adults with established rheumatoid arthritis: a cross-sectional study. BMC Musculoskeletal Disorders. 2016;17:22.
91. Bugdayci D, Paker N, Rezvani A, Kesiktas N, Yilmaz O, Sahin M, et al. Frequency and predictors for falls in the ambulatory patients with rheumatoid arthritis: A longitudinal prospective study. Rheumatology International. 2013;33(10):2523-7.
92. Ergül EE, Melikoğlu MA. A quantitative assessment of the risk of falls in rheumatoid arthritis patients and determination of the risk factors. Turkish Journal of Physical Medicine & Rehabilitation (2587-1250). 2022;68(2):271-7.
93. Furuya T, Yamagiwa K, Ikai T, Inoue E, Taniguchi A, Momohara S, et al. Associated factors for falls and fear of falling in Japanese patients with rheumatoid arthritis. Clinical Rheumatology. 2009;28(11):1325-30.
94. Hayashibara M, Hagino H, Katagiri H, Okano T, Okada J, Teshima R. Incidence and risk factors of falling in ambulatory patients with rheumatoid arthritis: a prospective 1-year study. Osteoporosis International. 2010;21(11):1825-33.
95. Lourenco MA, Carli F, de Assis MR. Characterization of falls in adults with established rheumatoid arthritis and associated factors. Advances in Rheumatology. 2018;58(1):16.

96. Lourenco MDA, Roma I, Assis MRD. Falls and their association with physical tests, functional capacity, clinical and demographic factors in patients with rheumatoid arthritis. Revista Brasileira de Reumatologia. 2017;57(3):217-23.
97. Mamoto K, Inui K, Okano T, Sugioka Y, Tada M, Koike T, et al. Incidence rate of falls and its risk factors in patients with rheumatoid arthritis compared to controls: Four years of the TOMORROW study. Modern Rheumatology. 2017;27(1):8-14.
98. Mikos M, Kucharska E, Lulek AM, Klosinski M, Batko B. Evaluation of Risk Factors for Falls in Patients with Rheumatoid Arthritis. Medical Science Monitor. 2020;26:e921862.
99. Smith TO, Clarke C, Dainty JR, Watts L, Yates M, Pomeroy VM, et al. Clinical and biomechanical factors associated with falls and rheumatoid arthritis: baseline cohort with longitudinal nested case–control study. Rheumatology. 2022;61(2):679-87.
100. Stanmore EK, Oldham J, Skelton DA, O'Neill T, Pilling M, Campbell AJ, et al. Risk factors for falls in adults with rheumatoid arthritis: a prospective study. Arthritis care & research. 2013;65(8):1251-8.
101. Wiegmann S, Armbrecht G, Borucki D, Buehring B, Buttgereit F, Detzer C, et al. Association between sarcopenia, physical performance and falls in patients with rheumatoid arthritis: a 1-year prospective study. BMC Musculoskeletal Disorders. 2021;22(1):885.
102. Wiegmann S, Armbrecht G, Borucki D, Buehring B, Buttgereit F, Detzer C, et al. Balance and prospective falls in patients with rheumatoid arthritis. BMC Musculoskeletal Disorders. 2022;23(1):549.
103. Yamagiwa K, Iijima S, Furuya T, Ikai T, Inoue E, Taniguchi A, et al. Incidence of falls and fear of falling in Japanese patients with rheumatoid arthritis. Modern Rheumatology. 2011;21(1):51-6.
104. Zonzini Gaino J, Barros Bertolo M, Silva Nunes C, de Morais Barbosa C, Sachetto Z, Davitt M, et al. Disease-related outcomes influence prevalence of falls in people with rheumatoid arthritis. Annals of Physical & Rehabilitation Medicine. 2019;62(2):84-91.
105. Mahdi MA, Erraoui M, Ngeuleu A, Tahiri L, Rkain H, Allali F. Falls, fear of falling and risk of falls in patients with rheumatoid arthritis: prevalence and associated factors. The Pan African Medical Journal. 2017;28(16).
106. Aurrecoechea E, Llorca Diaz J, Diez Lizuain ML, McGwin G, Jr., Calvo-Alen J. Gender-associated comorbidities in rheumatoid arthritis and their impact on outcome: data from GENIRA. Rheumatology International. 2017;37(4):479-85.
107. Sahatciu-Meka V, Rexhepi S, Manxhuka-Kerliu S, Rexhepi M. Extra-articular manifestations of seronegative and seropositive rheumatoid arthritis. Bosnian Journal of Basic Medical Sciences. 2010;10(1):26-31.
108. Al-Bishri J, Attar SM, Bassuni N, Al-Nofaiey Y, Qutbuddeen H, Al-Harthi S, et al. Comorbidity Profile Among Patients with Rheumatoid Arthritis and the Impact on Prescriptions Trend. Clinical Medicine Insights: Arthritis & Musculoskeletal Disorders. 2013(6):11-8.
109. Mazzantini M, Talarico R, Doveri M, Consensi A, Cazzato M, Bazzichi L, et al. Incident comorbidity among patients with rheumatoid arthritis treated or not with low-dose glucocorticoids: a retrospective study. Journal of Rheumatology. 2010;37(11):2232-6.
110. Brenton-Rule A, Dalbeth N, Menz HB, Bassett S, Rome K. Are Foot and Ankle Characteristics Associated With Falls in People With Rheumatoid Arthritis? A Prospective Study. Arthritis care & research. 2017;69(8):1150-5.
111. Cheng TT, Lai HM, Yu SF, Chiu WC, Hsu CY, Chen JF, et al. The impact of low-dose glucocorticoids on disease activity, bone mineral density, fragility fractures, and 10-year probability of fractures in patients with rheumatoid arthritis. Journal of Investigative Medicine. 2018;66(6):1004-7.
112. Kaz Kaz H, Johnson D, Kerry S, Chinappen U, Tweed K, Patel S. Fall-related risk factors and osteoporosis in women with rheumatoid arthritis. Rheumatology. 2004;43(10):1267-71.

113. Meng J, Li Y, Yuan X, Lu Y. Evaluating osteoporotic fracture risk with the Fracture Risk Assessment Tool in Chinese patients with rheumatoid arthritis. Medicine (United States). 2017;96(18) (no pagination).
114. Palmowski A, Boyadzhieva Z, Nielsen SM, Muche B, Hermann S, Boers M, et al. Sex and age do not modify the association between glucocorticoids and bone mineral density in patients with rheumatoid arthritis: a cross-sectional study. Arthritis Research and Therapy. 2023;25(1) (no pagination).
115. Rossini M, Viapiana O, Vitiello M, Malavolta N, La Montagna G, Maddali Bongi S, et al. Prevalence and incidence of osteoporotic fractures in patients on long-term glucocorticoid treatment for rheumatic diseases: The glucocorticoid induced OsTeoporosis TOol (GIOTTO) study. Reumatismo. 2017;69(1):30-9.
116. Shankar S, Handa R, Aneja R, Marwaha V, Ammini AC, Aprajita V. Bone mineral density in Indian women with rheumatoid arthritis. Rheumatology International. 2009;29(4):377-81.
117. Wilson JC, Sarsour K, Gale S, Petho-Schramm A, Jick SS, Meier CR. Incidence and Risk of Glucocorticoid-Associated Adverse Effects in Patients With Rheumatoid Arthritis. Arthritis care & research. 2019;71(4):498-511.
118. Abtahi S, Driessen JHM, Burden AM, Souverein PC, van den Bergh JP, van Staa TP, et al. Concomitant use of oral glucocorticoids and proton pump inhibitors and risk of osteoporotic fractures among patients with rheumatoid arthritis: a population-based cohort study. Annals of the Rheumatic Diseases. 2021;80(4):423-31.
119. Abtahi S, Driessen JHM, Burden AM, Souverein PC, Bergh JPvd, Staa TPv, et al. Low-dose oral glucocorticoid therapy and risk of osteoporotic fractures in patients with rheumatoid arthritis: a cohort study using the Clinical Practice Research Datalink. Rheumatology. 2022;61(4):1448-58.
120. Balasubramanian A, Wade S, Adler R, Lin C, Maricic M, O'Malley C, et al. Glucocorticoid exposure and fracture risk in patients with new-onset rheumatoid arthritis. Osteoporosis International. 2016;27(11):3239-49.
121. de Nijs RN, Jacobs JW, Bijlsma JW, Lems WF, Laan RF, Houben HH, et al. Prevalence of vertebral deformities and symptomatic vertebral fractures in corticosteroid treated patients with rheumatoid arthritis. Rheumatology. 2001;40(12):1375-83.
122. Hong WJ, Chen W, Yeo KJ, Huang PH, Chen DY, Lan JL. Increased risk of osteoporotic vertebral fracture in rheumatoid arthritis patients with new-onset cardiovascular diseases: a retrospective nationwide cohort study in Taiwan. Osteoporosis International. 2019;30(8):1617-25.
123. Kawai VK, Grijalva CG, Arbogast PG, Curtis JR, Solomon DH, Delzell E, et al. Initiation of tumor necrosis factor alpha antagonists and risk of fractures in patients with selected rheumatic and autoimmune diseases. Arthritis care & research. 2013;65(7):1085-94.
124. Lai EL, Huang WN, Chen HH, Hsu CY, Chen DY, Hsieh TY, et al. Ten-year fracture risk by FRAX and osteoporotic fractures in patients with systemic autoimmune diseases. Lupus. 2019;28(8):945-53.
125. Orstavik RE, Haugeberg G, Mowinckel P, Hoiseth A, Uhlig T, Falch JA, et al. Vertebral deformities in rheumatoid arthritis: a comparison with population-based controls. Archives of Internal Medicine. 2004;164(4):420-5.
126. Roubille C, Coffy A, Rincheval N, Dougados M, Flipo R-M, Daurès J-P, et al. Ten-year analysis of the risk of severe outcomes related to low-dose glucocorticoids in early rheumatoid arthritis. Rheumatology. 2021;60(8):3738-46.
127. Roussy JP, Bessette L, Bernatsky S, Rahme E, Lachaine J. Biologic disease-modifying anti-rheumatic drugs and the risk of non-vertebral osteoporotic fractures in patients with rheumatoid arthritis aged 50 years and over. Osteoporosis International. 2013;24(9):2483-92.

128. van Tuyl LH, Boers M, Lems WF, Landewe RB, Han H, van der Linden S, et al. Survival, comorbidities and joint damage 11 years after the COBRA combination therapy trial in early rheumatoid arthritis. Annals of the Rheumatic Diseases. 2010;69(5):807-12.
129. Kim SY, Schneeweiss S, Liu J, Solomon DH. Effects of disease-modifying antirheumatic drugs on nonvertebral fracture risk in rheumatoid arthritis: a population-based cohort study. J Bone Miner Res. 2012;27(4):789-96.
130. Vestergaard P, Rejnmark L, Mosekilde L. Fracture risk associated with use of nonsteroidal anti-inflammatory drugs, acetylsalicylic acid, and acetaminophen and the effects of rheumatoid arthritis and osteoarthritis. Calcified Tissue International. 2006;79(2):84-94.
131. Acurcio FA, Moura CS, Bernatsky S, Bessette L, Rahme E. Opioid Use and Risk of Nonvertebral Fractures in Adults with Rheumatoid Arthritis: A Nested Case-Control Study Using Administrative Databases. Arthritis and Rheumatology. 2016;68(1):83-91.
132. Amkreutz J, de Moel EC, Theander L, Willim M, Heimans L, Nilsson JA, et al. Association Between Bone Mineral Density and Autoantibodies in Patients With Rheumatoid Arthritis. Arthritis & Rheumatology. 2021;73(6):921-30.
133. Cheng TT, Yu SF, Su FM, Chen YC, Su BYJ, Chiu WC, et al. Anti-CCP-positive patients with RA have a higher 10-year probability of fracture evaluated by FRAX: A registry study of RA with osteoporosis/fracture. Arthritis Research and Therapy. 2018;20(1) (no pagination).
134. Ursum J, Britsemmer K, van Schaardenburg D, Lips PT, Dijkmans BA, Lems W. High prevalence of vertebral deformities in elderly patients with early rheumatoid arthritis. Annals of the rheumatic diseases. 2009;68(9):1512-3.
135. Chen J, Liu W, Lin Q, Chen L, Yin J, Huang H. Vitamin D deficiency and low bone mineral density in native Chinese rheumatoid arthritis patients. International Journal of Rheumatic Diseases. 2014;17(1):66-70.
136. Nakayama M, Furuya T, Inoue E, Tanaka E, Ikari K, Yamanaka H, et al. Vitamin D deficiency is a risk factor for new fractures in Japanese postmenopausal women with rheumatoid arthritis: results from the IORRA cohort study. Archives of Osteoporosis. 2021;16(1):119.
137. Tada M, Yamada Y, Mandai K, Hidaka N. Relationships of the stand-up time to falls and fractures in patients with rheumatoid arthritis: Results from the CHIKARA study. International Journal of Rheumatic Diseases. 2021;24(2):246-53.
138. Riesco M, Manzano F, Font P, Garcia A, Nolla JM. Osteoporosis in psoriatic arthritis: an assessment of densitometry and fragility fractures. Clinical Rheumatology. 2013;32(12):1799-804.
139. Frediani B, Allegri A, Falsetti P, Storri L, Bisogno S, Baldi F, et al. Bone mineral density in patients with psoriatic arthritis. Journal of Rheumatology. 2001;28(1):138-43.
140. Grazio S, Cvijetic S, Vlak T, Grubisic F, Matijevic V, Nemcic T, et al. Osteoporosis in psoriatic arthritis: is there any? Wien Klin Wochenschr. 2011;123(23-24):743-50.
141. Wang Y, Song ZB, Deng XR, Zhang XH, Zhang ZL. Risk factors associated with osteoporosis and fracture in psoriatic arthritis. Chinese Medical Journal. 2021;134(21):2564-72.
142. Del Puente A, Esposito A, Costa L, Benigno C, Del Puente A, Foglia F, et al. Fragility Fractures in Patients with Psoriatic Arthritis. Journal of Rheumatology - Supplement. 2015;93:36-9.
143. Geusens P, De Winter L, Quaden D, Vanhoof J, Vosse D, van den Bergh J, et al. The prevalence of vertebral fractures in spondyloarthritis: relation to disease characteristics, bone mineral density, syndesmophytes and history of back pain and trauma. Arthritis research & therapy. 2015;17:294.
144. Pedreira PG, Pinheiro MM, Szejnfeld VL. Bone mineral density and body composition in postmenopausal women with psoriasis and psoriatic arthritis. Arthritis research & therapy. 2011;13(1):R16.

145. Muzevic MV, Biljan D, Sola M, Kuric I, Muzevic D, Mosler EL. Mineral Bone Density and Vitamin D Levels in Patients with Psoriatic Arthritis. Acta Clinica Croatica. 2022;61(1):70-8.
146. Kathuria P, Gordon KB, Silverberg JI. Association of psoriasis and psoriatic arthritis with osteoporosis and pathological fractures. J Am Acad Dermatol. 2017;76(6):1045-53.e3.
147. Carter K, Walmsley S, Oliffe M, Hassett G, Turner DE. Increased falls risk in people with psoriatic arthritis-related foot problems: a novel finding. Rheumatology. 2021;60(2):976-7.
148. Ross Y, Jaleel S, Magrey M. Racial disparities in comorbidities of patients with psoriatic arthritis. Rheumatology International. 2023;43(8):1525-9.
149. Busquets N, Vaquero CG, Moreno JR, Vilaseca DR, Narvaez J, Carmona L, et al. Bone mineral density status and frequency of osteoporosis and clinical fractures in 155 patients with psoriatic arthritis followed in a university hospital. Reumatologia Clinica. 2014;10(2):89-93.
150. Borman P, Babaoglu S, Gur G, Bingol S, Bodur H. Bone mineral density and bone turnover in patients with psoriatic arthritis. Clinical Rheumatology. 2008;27(4):443-7.
151. Halasi A, Szegedi A, Torocsik D, Varga J, Farmasi N, Szucs G, et al. Psoriatic arthritis and its special features predispose not only for osteoporosis but also for fractures and falls. Journal of Dermatology. 2023;50(5):608-14.
152. Arends S, Spoorenberg A, Bruyn GA, Houtman PM, Leijsma MK, Kallenberg CG, et al. The relation between bone mineral density, bone turnover markers, and vitamin D status in ankylosing spondylitis patients with active disease: a cross-sectional analysis. Osteoporosis International. 2011;22(5):1431-9.
153. Hu LY, Lu T, Chen PM, Shen CC, Hung YM, Hsu CL. Should clinicians pay more attention to the potential underdiagnosis of osteoporosis in patients with ankylosing spondylitis? A national population-based study in Taiwan. PLoS ONE [Electronic Resource]. 2019;14(2):e0211835.
154. Karberg K, Zochling J, Sieper J, Felsenberg D, Braun J. Bone loss is detected more frequently in patients with ankylosing spondylitis with syndesmophytes. Journal of Rheumatology. 2005;32(7):1290-8.
155. Klingberg E, Geijer M, Gothlin J, Mellstrom D, Lorentzon M, Hilme E, et al. Vertebral fractures in ankylosing spondylitis are associated with lower bone mineral density in both central and peripheral skeleton. Journal of Rheumatology. 2012;39(10):1987-95.
156. Klingberg E, Lorentzon M, Mellstrom D, Geijer M, Gothlin J, Hilme E, et al. Osteoporosis in ankylosing spondylitis - prevalence, risk factors and methods of assessment. Arthritis research & therapy. 2012;14(3):R108.
157. Magrey MN, Lewis S, Asim Khan M. Utility of DXA scanning and risk factors for osteoporosis in ankylosing spondylitis-A prospective study. Seminars in Arthritis and Rheumatism. 2016;46(1):88-94.
158. Mermerci Baskan B, Pekin Dogan Y, Sivas F, Bodur H, Ozoran K. The relation between osteoporosis and vitamin D levels and disease activity in ankylosing spondylitis. Rheumatology International. 2010;30(3):375-81.
159. Ulu MA, Batmaz I, Dilek B, Cevik R. Prevalence of osteoporosis and vertebral fractures and related factors in patients with ankylosing spondylitis. Chinese Medical Journal. 2014;127(15):2740-7.

160. van der Weijden MA, van Denderen JC, Lems WF, Nurmohamed MT, Dijkmans BA, van der Horst-Bruinsma IE. Etanercept Increases Bone Mineral Density in Ankylosing Spondylitis, but Does Not Prevent Vertebral Fractures: Results of a Prospective Observational Cohort Study. Journal of Rheumatology. 2016;43(4):758-64.
161. Vasdev V, Bhakuni D, Garg MK, Narayanan K, Jain R, Chadha D. Bone mineral density in young males with ankylosing spondylitis. International Journal of Rheumatic Diseases. 2011;14(1):68-73.
162. Wang D, Hou Z, Gong Y, Chen S, Lin L, Xiao Z. Bone edema on magnetic resonance imaging is highly associated with low bone mineral density in patients with ankylosing spondylitis. PLoS ONE [Electronic Resource]. 2017;12(12):e0189569.
163. Wang DM, Zeng QY, Chen SB, Gong Y, Hou ZD, Xiao ZY. Prevalence and risk factors of osteoporosis in patients with ankylosing spondylitis: a 5-year follow-up study of 504 cases. Clin Exp Rheumatol. 2015;33(4):465-70.
164. Wang L, Gao L, Jin D, Wang P, Yang B, Deng W, et al. The Relationship of Bone Mineral Density to Oxidant/Antioxidant Status and Inflammatory and Bone Turnover Markers in a Multicenter Cross-Sectional Study of Young Men with Ankylosing Spondylitis. Calcified Tissue International. 2015;97(1):12-22.
165. Hmamouchi I, Allali F, El Handaoui B, Amine H, Rostom S, Abouqal R, et al. The relation between disease activity, vitamin D levels and bone mineral density in men patients with ankylosing spondylitis. Rheumatology Reports. 2013;5(1):e3-e.
166. Feldtkeller E, Vosse D, Geusens P, van der Linden S. Prevalence and annual incidence of vertebral fractures in patients with ankylosing spondylitis. Rheumatology International. 2006;26(3):234-9.
167. Jun JB, Joo KB, Her MY, Kim TH, Bae SC, Yoo DH, et al. Femoral bone mineral density is associated with vertebral fractures in patients with ankylosing spondylitis: a cross-sectional study. Journal of Rheumatology. 2006;33(8):1637-41.
168. Kang KY, Kim IJ, Jung SM, Kwok SK, Ju JH, Park KS, et al. Incidence and predictors of morphometric vertebral fractures in patients with ankylosing spondylitis. Arthritis research & therapy. 2014;16(3):R124.
169. Killinger Z, Kuzma M, Tomkova S, Brazdilova K, Jackuliak P, Payer J. Prediction of Vertebral Fractures by Trabecular Bone Score in Patients With Ankylosing Spondylitis. Physiological research. 2021;70(Supplement 1):S53-S60.
170. Ljung L, Sundstrom B, Smeds J, Ketonen M, Forsblad-d'Elia H. Patterns of comorbidity and disease characteristics among patients with ankylosing spondylitis-a cross-sectional study. Clinical Rheumatology. 2018;37(3):647-53.
171. Maas F, Spoorenberg A, Brouwer E, Schilder AM, Chaudhry RN, Wink F, et al. Radiographic vertebral fractures develop in patients with ankylosing spondylitis during 4 years of TNF-alpha blocking therapy. Clin Exp Rheumatol. 2016;34(2):191-9.
172. Maas F, Spoorenberg A, van der Slik BPG, van der Veer E, Brouwer E, Bootsma H, et al. Clinical Risk Factors for the Presence and Development of Vertebral Fractures in Patients With Ankylosing Spondylitis. Arthritis care & research. 2017;69(5):694-702.
173. Mitra D, Elvins DM, Speden DJ, Collins AJ. The prevalence of vertebral fractures in mild ankylosing spondylitis and their relationship to bone mineral density. Rheumatology. 2000;39(1):85-9.
174. Montala N, Juanola X, Collantes E, Munoz-Gomariz E, Gonzalez C, Gratacos J, et al. Prevalence of vertebral fractures by semiautomated morphometry in patients with ankylosing spondylitis. Journal of Rheumatology. 2011;38(5):893-7.
175. Rossini M, Viapiana O, Idolazzi L, Ghellere F, Fracassi E, Troplini S, et al. Higher Level of Dickkopf-1 is Associated with Low Bone Mineral Density and Higher Prevalence of Vertebral Fractures in Patients with Ankylosing Spondylitis. Calcified Tissue International. 2016;98(5):438-45.

176. Vosse D, Landewe R, van der Heijde D, van der Linden S, van Staa TP, Geusens P. Ankylosing spondylitis and the risk of fracture: results from a large primary care-based nested case-control study. Annals of the Rheumatic Diseases. 2009;68(12):1839-42.
177. Ghozlani I, Ghazi M, Nouijai A, Mounach A, Rezqi A, Achemlal L, et al. Prevalence and risk factors of osteoporosis and vertebral fractures in patients with ankylosing spondylitis. Bone. 2009;44(5):772-6.
178. Dursun N, Sarikaya S, Ozdolap S, Dursun E, Zateri C, Altan L, et al. Risk of falls in patients with ankylosing spondylitis. Journal of Clinical Rheumatology. 2015;21(2):76-80.
179. Bremander A, Petersson IF, Bergman S, Englund M. Population-based estimates of common comorbidities and cardiovascular disease in ankylosing spondylitis. Arthritis care & research. 2011;63(4):550-6.
180. Deminger A, Klingberg E, Lorentzon M, Geijer M, Gothlin J, Hedberg M, et al. Which measuring site in ankylosing spondylitis is best to detect bone loss and what predicts the decline: results from a 5-year prospective study. Arthritis research & therapy. 2017;19(1):273.
181. Tsur AM, David P, Watad A, Nissan D, Cohen AD, Amital H. Ankylosing Spondylitis and the Risk of Hip Fractures: a Matched Cohort Study. Journal of General Internal Medicine. 2022;37(13):3283-8.
182. Walsh JA, Song X, Kim G, Park Y. Evaluation of the comorbidity burden in patients with ankylosing spondylitis using a large US administrative claims data set. Clinical Rheumatology. 2018;37(7):1869-78.
183. Bengtsson K, Askling J, Lorentzon M, Rosengren B, Deminger A, Klingberg E, et al. Occurrence and relative risks for non-vertebral fractures in patients with ankylosing spondylitis compared with the general population: a register-based study from Sweden. RMD Open. 2023;9(1):02.
184. Beek KJ, Rusman T, van der Weijden MAC, Lems WF, van Denderen JC, Konsta M, et al. Long-Term Treatment With TNF-Alpha Inhibitors Improves Bone Mineral Density But Not Vertebral Fracture Progression in Ankylosing Spondylitis. J Bone Miner Res. 2019;34(6):1041-8.
185. El Maghraoui A, Ebo'o FB, Sadni S, Majjad A, Hamza T, Mounach A. Is there a relation between pre-sarcopenia, sarcopenia, cachexia and osteoporosis in patients with ankylosing spondylitis? BMC Musculoskeletal Disorders. 2016;17:268.
186. Munoz-Ortego J, Vestergaard P, Rubio JB, Wordsworth P, Judge A, Javaid MK, et al. Ankylosing spondylitis is associated with an increased risk of vertebral and nonvertebral clinical fractures: a population-based cohort study. J Bone Miner Res. 2014;29(8):1770-6.
187. Prieto-Alhambra D, Munoz-Ortego J, De Vries F, Vosse D, Arden NK, Bowness P, et al. Ankylosing spondylitis confers substantially increased risk of clinical spine fractures: a nationwide case-control study. Osteoporosis International. 2015;26(1):85-91.
188. Toussirot E, Michel F, Wendling D. Bone density, ultrasound measurements and body composition in early ankylosing spondylitis. Rheumatology. 2001;40(8):882-8.
189. Borman P, Bodur H, Bingol N, Bingol S, Bostan EE. Bone mineral density and bone turnover markers in a group of male ankylosing spondylitis patients: Relationship to disease activity. Journal of Clinical Rheumatology. 2001;7(5):315-21.
190. Grazio S, Kusic Z, Cvijetic S, Grubisic F, Balenovic A, Nemcic T, et al. Relationship of bone mineral density with disease activity and functional ability in patients with ankylosing spondylitis: a cross-sectional study. Rheumatology International. 2012;32(9):2801-8.
191. Muntean L, Rojas-Vargas M, Font P, Simon SP, Rednic S, Schiotis R, et al. Relative value of the lumbar spine and hip bone mineral density and bone turnover markers in men with ankylosing spondylitis. Clinical Rheumatology. 2011;30(5):691-5.

192. Lange U, Teichmann J, Strunk J, Muller-Ladner U, Schmidt KL. Association of 1.25 vitamin D3 deficiency, disease activity and low bone mass in ankylosing spondylitis. Osteoporosis International. 2005;16(12):1999-2004.
193. Korczowska I, Przepiera-Bedzak H, Brzosko M, Lacki JK, Trefler J, Hrycaj P. Bone tissue metabolism in men with ankylosing spondylitis. Advances in Medical Sciences. 2011;56(2):264-9.
194. Almehed K, Forsblad d'Elia H, Kvist G, Ohlsson C, Carlsten H. Prevalence and risk factors of osteoporosis in female SLE patients-extended report. Rheumatology. 2007;46(7):1185-90.
195. Banno S, Matsumoto Y, Naniwa T, Hayami Y, Sugiura Y, Yoshinouchi T, et al. Reduced bone mineral density in Japanese premenopausal women with systemic lupus erythematosus treated with glucocorticoids. Modern Rheumatology. 2002;12(4):323-8.
196. Becker A, Fischer R, Scherbaum WA, Schneider M. Osteoporosis screening in systemic lupus erythematosus: impact of disease duration and organ damage. Lupus. 2001;10(11):809-14.
197. Bhattoa HP, Bettembuk P, Balogh A, Szegedi G, Kiss E. Bone mineral density in women with systemic lupus erythematosus. Clinical Rheumatology. 2002;21(2):135-41.
198. Bhattoa HP, Kiss E, Bettembuk P, Balogh A. Bone mineral density, biochemical markers of bone turnover, and hormonal status in men with systemic lupus erythematosus. Rheumatology International. 2001;21(3):97-102.
199. Bultink IE, Lems WF, Kostense PJ, Dijkmans BA, Voskuyl AE. Prevalence of and risk factors for low bone mineral density and vertebral fractures in patients with systemic lupus erythematosus. Arthritis & Rheumatism. 2005;52(7):2044-50.
200. Correa-Rodríguez M, Pocovi-Gerardino G, Callejas-Rubio J-L, Ríos-Fernández R, Rueda-Medina B, Ortego-Centeno N. Disease Damage Accrual and Low Bone Mineral Density in Female Patients with Systemic Lupus Erythematosus. Biological Research for Nursing. 2021;23(4):575-83.
201. Cramarossa G, Urowitz MB, Su J, Gladman D, Touma Z. Prevalence and associated factors of low bone mass in adults with systemic lupus erythematosus. Lupus. 2017;26(4):365-72.
202. Furukawa M, Kiyohara C, Tsukamoto H, Mitoma H, Kimoto Y, Uchino A, et al. Prevalence of and risk factors for low bone mineral density in Japanese female patients with systemic lupus erythematosus. Rheumatology International. 2011;31(3):365-76.
203. Garelick D, Pinto SM, Farinha F, Pires T, Khan E, Isenberg D. Fracture risk in systemic lupus erythematosus patients over 28 years. Rheumatology. 2021;60(6):2765-72.
204. Jacobs J, Korswagen LA, Schilder AM, van Tuyl LH, Dijkmans BA, Lems WF, et al. Six-year follow-up study of bone mineral density in patients with systemic lupus erythematosus. Osteoporosis International. 2013;24(6):1827-33.
205. Le B, Waller JL, Radhakrishnan R, Oh SJ, Kheda MF, Nahman NS, Jr., et al. Osteoporotic fractures in patients with systemic lupus erythematosus and end stage renal disease. Lupus. 2018;27(1):17-24.
206. Li EK, Tam LS, Griffith JF, Zhu TY, Li TK, Li M, et al. High prevalence of asymptomatic vertebral fractures in Chinese women with systemic lupus erythematosus. Journal of Rheumatology. 2009;36(8):1646-52.
207. Mendoza-Pinto C, Etchegaray-Morales I, Munguia-Realpozo P, Mendez-Martinez S, Ayon-Aguilar J, Arellano-Avendano F, et al. SLICC-Frailty Index and Its Association with Low Bone Mineral Density and Vertebral Fractures in Women with Systemic Lupus Erythematosus. Calcified Tissue International. 2023;113(5):475-80.

208. Mendoza-Pinto C, Garcia-Carrasco M, Juarez-Melchor D, Munguia-Realpozo P, Etchegaray-Morales I, Santiago-Martin N, et al. A Retrospective Analysis of Longitudinal Changes in Bone Mineral Density in Women with Systemic Lupus Erythematosus. Calcified Tissue International. 2021;109(4):363-71.
209. Mendoza-Pinto C, Garcia-Carrasco M, Sandoval-Cruz H, Munoz-Guarneros M, Escarcega RO, Jimenez-Hernandez M, et al. Risk factors of vertebral fractures in women with systemic lupus erythematosus. Clinical Rheumatology. 2009;28(5):579-85.
210. Mok CC, Wong SN, Ma KM. Childhood-onset disease carries a higher risk of low bone mineral density in an adult population of systemic lupus erythematosus. Rheumatology. 2012;51(3):468-75.
211. Pineau CA, Urowitz MB, Fortin PJ, Ibanez D, Gladman DD. Osteoporosis in systemic lupus erythematosus: factors associated with referral for bone mineral density studies, prevalence of osteoporosis and factors associated with reduced bone density. Lupus. 2004;13(6):436-41.
212. Rees F, Doherty M, Grainge M, Lanyon P, Davenport G, Zhang W. Burden of Comorbidity in Systemic Lupus Erythematosus in the UK, 1999-2012. Arthritis care & research. 2016;68(6):819-27.
213. Salman-Monte TC, Torrente-Segarra V, Munoz-Ortego J, Mojal S, Carbonell-Abello J. Prevalence and predictors of low bone density and fragility fractures in women with systemic lupus erythematosus in a Mediterranean region. Rheumatology International. 2015;35(3):509-15.
214. Souto MI, Coelho A, Guo C, Mendonca LM, Pinheiro MF, Papi JA, et al. The prevalence of low bone mineral density in Brazilian patients with systemic lupus erythematosus and its relationship with the disease damage index and other associated factors. Journal of Clinical Densitometry. 2012;15(3):320-7.
215. Sun Y-N, Feng X-Y, He L, Zeng L-X, Hao Z-M, Lv X-H, et al. Prevalence and Possible Risk Factors of Low Bone Mineral Density in Untreated Female Patients with Systemic Lupus Erythematosus. BioMed Research International. 2015;2015:1-7.
216. Yeap SS, Fauzi AR, Kong NC, Halim AG, Soehardy Z, Rahimah S, et al. Influences on bone mineral density in Malaysian premenopausal systemic lupus erythematosus patients on corticosteroids. Lupus. 2009;18(2):178-81.
217. Yee CS, Crabtree N, Skan J, Amft N, Bowman S, Situnayake D, et al. Prevalence and predictors of fragility fractures in systemic lupus erythematosus. Annals of the Rheumatic Diseases. 2005;64(1):111-3.
218. Bonfa AC, Seguro LP, Caparbo V, Bonfa E, Pereira RM. RANKL and OPG gene polymorphisms: associations with vertebral fractures and bone mineral density in premenopausal systemic lupus erythematosus. Osteoporosis International. 2015;26(5):1563-71.
219. Borba VZ, Matos PG, da Silva Viana PR, Fernandes A, Sato EI, Lazaretti-Castro M. High prevalence of vertebral deformity in premenopausal systemic lupus erythematosus patients. Lupus. 2005;14(7):529-33.
220. Bultink IE, Harvey NC, Lalmohamed A, Cooper C, Lems WF, van Staa TP, et al. Elevated risk of clinical fractures and associated risk factors in patients with systemic lupus erythematosus versus matched controls: a population-based study in the United Kingdom. Osteoporosis International. 2014;25(4):1275-83.
221. Dey M, Bukhari M. Predictors of fracture risk in patients with systemic lupus erythematosus. Lupus. 2018;27(9):1547-51.
222. Ekblom-Kullberg S, Kautiainen H, Alha P, Leirisalo-Repo M, Julkunen H. Frequency of and risk factors for symptomatic bone fractures in patients with systemic lupus erythematosus. Scandinavian Journal of Rheumatology. 2013;42(5):390-3.
223. Furukawa M, Kiyohara C, Horiuchi T, Tsukamoto H, Mitoma H, Kimoto Y, et al. Prevalence and risk factors of vertebral fracture in female Japanese patients with systemic lupus erythematosus. Modern Rheumatology. 2013;23(4):765-73.

224. Garcia-Carrasco M, Mendoza-Pinto C, Leon-Vazquez ML, Mendez-Martinez S, Etchegaray-Morales I, Montiel-Jarquin A, et al. Incidence of Vertebral Fractures in Women with Systemic Lupus Erythematosus After 8 Years of Follow-Up. Calcified Tissue International. 2017;101(3):291-9.
225. Kim CS, Han KD, Jung JH, Choi HS, Bae EH, Ma SK, et al. Incidence and risk factors for osteoporotic fractures in patients with systemic lupus erythematosus versus matched controls. Korean Journal of Internal Medicine. 2021;36(1):154-63.
226. Lai EL, Huang WN, Chen HH, Chen JP, Chen DY, Hsieh TY, et al. Degraded microarchitecture by low trabecular bone score is associated with prevalent vertebral fractures in patients with systemic lupus erythematosus. Archives of Osteoporosis. 2020;15(1):54.
227. Lee C, Almagor O, Dunlop DD, Manzi S, Spies S, Ramsey-Goldman R. Self-reported fractures and associated factors in women with systemic lupus erythematosus. Journal of Rheumatology. 2007;34(10):2018-23.
228. Mok CC, Ying SKY, Chi HT, Kwok MM. Bone mineral density and body composition in men with systemic lupus erythematosus: A case control study. Bone. 2008;43(2):327-31.
229. Rhew EY, Lee C, Eksarko P, Dyer AR, Tily H, Spies S, et al. Homocysteine, bone mineral density, and fracture risk over 2 years of followup in women with and without systemic lupus erythematosus. Journal of Rheumatology. 2008;35(2):230-6.
230. Tedeschi SK, Kim SC, Guan H, Costenbader KH, Grossman JM. Comparative Fracture Risks Among United States Medicaid Enrollees With and Those Without Systemic Lupus Erythematosus. Arthritis & Rheumatology. 2019;71(7):1141-6.
231. Wang SH, Chang YS, Liu CJ, Lai CC, Chen WS, Chen TJ, et al. Association of systemic lupus erythematosus with a higher risk of cervical but not trochanteric hip fracture: a nationwide population-based study. Arthritis care & research. 2013;65(10):1674-81.
232. Zhu TY, Griffith JF, Au SK, Tang XL, Kwok AW, Leung PC, et al. Incidence of and risk factors for non-vertebral and vertebral fracture in female Chinese patients with systemic lupus erythematosus: a five-year cohort study. Lupus. 2014;23(9):854-61.
233. Lee JJ, Aghdassi E, Cheung AM, Morrison S, Cymet A, Peeva V, et al. Ten-year absolute fracture risk and hip bone strength in Canadian women with systemic lupus erythematosus. Journal of Rheumatology. 2012;39(7):1378-84.
234. Lakshminarayanan S, Walsh S, Mohanraj M, Rothfield N. Factors associated with low bone mineral density in female patients with systemic lupus erythematosus. Journal of Rheumatology. 2001;28(1):102-8.
235. Mendoza-Pinto C, Garcia-Carrasco M, Jimenez-Hernandez M, Sanchez-Perez R, Escarcega RO, Nava-Zavala A, et al. Carotid atherosclerosis is not associated with lower bone mineral density and vertebral fractures in patients with systemic lupus erythematosus. Lupus. 2015;24(1):25-31.
236. Franco CJV, Henao MPZ, Pineda FL, Montoya SP, Vallejo EO, Padilla LMR, et al. Low bone mass and osteoporosis in patients with systemic lupus erythematosus. Revista Colombiana de Reumatología (English Edition). 2017;24(1):4-10.
237. Chong HC, Chee SS, Goh EM, Chow SK, Yeap SS. Dietary calcium and bone mineral density in premenopausal women with systemic lupus erythematosus. Clinical Rheumatology. 2007;26(2):182-5.
238. Lukasiewicz AM, Bohl DD, Varthi AG, Basques BA, Webb ML, Samuel AM, et al. Spinal fracture in patients with ankylosing spondylitis. Spine. 2016;41(3):191-6.
239. Boyanov M, Robeva R, Popivanov P. Bone mineral density changes in women with systemic lupus erythematosus. Clinical Rheumatology. 2003;22(4-5):318-23.

240. Chen HL, Shen LJ, Hsu PN, Shen CY, Hall SA, Hsiao FY. Cumulative Burden of Glucocorticoid-related Adverse Events in Patients with Systemic Lupus Erythematosus: Findings from a 12-year Longitudinal Study. Journal of Rheumatology. 2018;45(1):83-9.
241. Uaratanawong S, Deesomchoke U, Lertmaharit S, Uaratanawong S. Bone mineral density in premenopausal women with systemic lupus erythematosus. Journal of Rheumatology. 2003;30(11):2365-8.
242. Almehed K, Hetenyi S, Ohlsson C, Carlsten H, Forsblad-d'Elia H. Prevalence and risk factors of vertebral compression fractures in female SLE patients. Arthritis Research and Therapy. 2010;12(4) (no pagination).
243. Heshin-Bekenstein M, Trupin L, Yelin E, von Scheven E, Yazdany J, Lawson EF. Longitudinal disease- and steroid-related damage among adults with childhood-onset systemic lupus erythematosus. Seminars in Arthritis and Rheumatism. 2019;49(2):267-72.
244. Zonana-Nacach A, Barr SG, Magder LS, Petri M. Damage in systemic lupus erythematosus and its association with corticosteroids. Arthritis & Rheumatism. 2000;43(8):1801-8.
245. Mok CC, Mak A, Ma KM. Bone mineral density in postmenopausal Chinese patients with systemic lupus erythematosus. Lupus. 2005;14(2):106-12.
