# Supplementary material for: Epidemiology and factors associated with osteoporosis, falls and fractures in patients with chronic inflammatory rheumatic disease: a scoping review
Source: BMJ Open. 2025 Jul 28;15(7):e096226. doi: 10.1136/bmjopen-2024-096226 (PMC12306236; doi:10.1136/bmjopen-2024-096226)
Supplement: Supplementary file 6 [file bmjopen-2024-096226.reviewer_comments.pdf]

# PEER REVIEW HISTORY

BMJ Open publishes all reviews undertaken for accepted manuscripts. Reviewers are asked to complete a checklist review form and are provided with free text boxes to elaborate on their assessment. These free text comments are reproduced below.

## ARTICLE DETAILS

### Title (Provisional)

The epidemiology and factors associated with osteoporosis, falls and fractures in patients with chronic inflammatory rheumatic disease: A scoping review

### Authors

Cho, Catherine; Bak, Grace; Richards, Bethan; Sherrington, Catherine

---

## VERSION 1 - REVIEW

---

**Reviewer** 1

**Name** Essouma, Mickael

**Affiliation** Faculty of Medicine and Biomedical Sciences, University of Yaoundé I, Yaoundé, Cameroon, Department of Internal Medicine and Specialties

**Date** 10-Jan-2025

**COI** None

---

Peer review report for manuscript bmjopen-2024-096226 titled "The epidemiology and risk factors for osteoporosis, falls and fractures in patients with chronic inflammatory rheumatic disease: A scoping review"

Overview and general comment

The authors have scoped the literature on the epidemiology of osteporosis and falls in people with SLE< RA and spondyloarthritis. This is review is commendable because there is a lack of adequate data synthesis on this topic. However, the review needs a thorough revision before being accepted for publication. M

Specific comments

They are appended in the attached pdf manuscript received.

Review report available on 10/01/2025

****** The reviewer provided a marked copy with additional comments. Please contact the publisher for full details.** ****

| | |
|---|---|
| **Reviewer** | **2** |
| **Name** | **Blank, Robert D** |
| **Affiliation** | **Medical College of Wisconsin** |
| **Date** | **05-Apr-2025** |
| **COI** | **I receive editorial stipends from Elsevier.** |

**I receive royalties from Wolters Kluwer.**
**I consult for GLG and Guidepoint.**
**I hold ownership interests in Abbott Laboratories, AbbVie, Amgen, AstraZeneca, Bio-Rad Labs, Chugai, Doctorpedias, Eli Lilly, Glaxo, Illumina, JangoBio, Johnson & Johnson, Merck, Novartis, Novo Nordisk, Nutrien, Procter & Gamble, ROMTech, Sandoz, Sanofi, Siemens, Teva, and UCB.**
**I hold a leadership position in the International Federation of Musculoskeletal Research Societies.**

---

Overall Considerations: This is a carefully conducted and clearly reported scoping review of observational studies reporting associations of 4 inflammatory rheumatic diseases with osteoporosis, fractures, and falls. Limitations of this review reflect limitations of the literature on the topic. I've offered a few suggestions for improving this useful work.

Major Points:

1. It would be helpful if you were to assess the uniformity of the inclusion criteria for each of the 4 diseases considered. Does this literature suffer from inconsistent definition of the at-risk population?

2. Similarly, it would be helpful to address how outcomes were defined and assessed in the papers you reviewed. This is particularly important in gauging the uniformity of what was studied. With regard to fractures, please add information about sites studied/included/excluded. With regard to osteoporosis, what were diagnostic criteria and were these limited solely to BMD or were clinical criteria also included? With regard to falls, please include information about specific inclusion/exclusion criteria. Readers will want to know about methodological heterogeneity. You might consider (but need not) proposing criteria for defining outcomes.

3. It would be helpful if tables 2-5 included bibliography numbers for the relevant papers.

Minor Points:

1. Figure 1 (matrix) has minimal informative value, would either delete or add a scale to give symbol sizes meaning.

2. There is a recently published update to ref 1.

## VERSION 1 - AUTHOR RESPONSE

Reply to Reviewer 1:

1. **While I agree with the information provided in the first two paragraph of this introduction, this one is incomplete. Specify the rationale of the study. This sentence "This is in the landscape of improving disease control, with increasing expectations for physicians to manage related co-morbidities, which carry significant morbidity and mortality in those with CIRDs. Our research is focussed on better understanding "how big," the problem of osteoporosis, falls and related fractures are in this high-risk patient cohort and "how can we do better." on lines 5-9 (page 1) could help you specify the rationale of this review.**

Thank you for your comment. This scoping review forms the first part of our current research program, which is inclusive of several other studies stemming from the gaps in literature identified in this scoping review. We have added a further rationale for the study in the Background (Paragraph 2 and 3).

2. **Furthermore, why did you choose a scoping review over a systematic review and meta-analysis or other data synthesis vehicles?**

We chose a scoping review rather than a systematic review as we had the broader aim to understand and explore the breadth of literature currently available in the epidemiology and factors associated with osteoporosis, falls and osteoporotic fractures in individuals with established CIRDs, to identify gaps in literature and areas for improvement in design of future observational studies. Specifically for our team, this scoping review identified a need for Australian epidemiological studies in osteoporosis, falls and fractures in CIRD, which we are currently undertaking as a separate study, inclusive of assessment of lifestyle and socio-demographic risk factors.

Additionally, the number of associated factors identified in this scoping review confirms that it would not have been practical to conduct a single systematic reviews and meta-analyses. Our review enabled assessment of the feasibility of focussed systematic reviews on specific risk factors for osteoporosis, falls and fractures in CIRD which confirms the usefulness of the scoping review approach.

3. **This justification is incomplete. Why was it important to set the beginning of the search period at the time when infliximab was introduced in the therapeutic arsenal of RA? DOes infliximab have a proven influence on osteoporosis (including osteoporotic fractures) and fall risk in the studied populations?**

This is a very valid comment, and we have made amendments to the described Search Strategy to make our reasoning clearer. The rationale for January 2000 as the time of introduction of infliximab is the shift in therapeutic landscape with introduction of

bDMARDs has dramatically altered disease outcomes. We wanted our scoping review to reflect the current rheumatic disease population for greater external validity. We do not have direct evidence that these therapeutic advances have affected fracture and fall risk but we anticipate that the improved functional outcomes would translate to reduced fall risk so did not consider it useful to consider older studies.

4. **Did you undertake a consultation exercise with other colleagues or experts to help fine-tune the quality of data included in your scoping review as recommended by some experts in scoping reviews?**

We did not undertake a formal consultation exercise, but we did undertake informal consultation during multiple stages of this scoping review. For example, the design of the scoping review was conducted with input from researchers and multidisciplinary clinicians with expertise in falls, fractures and rheumatic disease. Our search strategy was completed in consultation with a librarian from our institution with experience in conducting scoping reviews. Given the informal nature of our consultation exercises and the lack of consensus on reporting recommendations, we did not specifically report this in our manuscript.

Buus N, Nygaard L, Berring LL, et al. Arksey and O′ Malley's consultation exercise in scoping reviews: A critical review. Journal of advanced nursing. 2022;78(8):2304-12.

5. **I suggest presenting the results in way such that the parameters of interest (osteoporosis, osteoporotic fractures, falls) are sub-sections the review and data for each of those parameters are presented separately for each disease. results by disease category.**

Thank you for this very valid opinion. We considered presenting the results with parameters of interest as subsections, rather than separated by disease. However, we prefer to present the results by each disease category to summarise the current literature available on the epidemiology and factors associated with osteoporosis, falls and osteoporotic fractures within each of these conditions, as we considered this approach provided clearer guidance to treating rheumatologists about the current state of the literature. We consider our current approach more clearly highlights the paucity of literature in PsA and AS, in comparison to RA and SLE; and in falls, compared to osteoporosis and fractures.

6. **Furthermore, in my perspective, you should not pool estimates from studies with considerable methodological differences. If they talk about risk factors, readers are expected to see risk factor metrics (e.g., relative risks, risk ratios, harad ratios) throughout the description of those risk factors. Otherwise, they should consider replacing the terminology "risk factors" with "associated factors" throughout the manuscript. When assessing associated factors, they should try their best to present those factors as described in evidence-based reports on risk/associated factors of osteoporosis, osteoporotic fractures and falls in the published general health literature.**

This is a very valid point. We have made amendments throughout the manuscript, changing risk factors to associated factors. Additionally, we have changed the title of our manuscript. Our results are descriptive on the number of studies reporting on associated factors and are not pooled estimates.

7. **Articles meeting inclusion criteria to be presented in the main body.**

Thank you for your comment. Ideally, we would present the entire table in the main body of the article, however, given the number of articles identified for inclusion in the scoping review (n=279), we felt the table was too large for inclusion in the main text, thus is available in Appendix 3. As per reviewer 2's feedback, we have included direct references on studies reporting on relevant associated factors in Appendix 5.

8. **Why merging data from many studies that are likely heterogeneous in terms of methodology? Same remark for results provided in table 2? This concern should be addressed throughout the results section and lead to the revision of the discussion section as well. I ws expecting ranges of estimates given methodological differences not pooled estimates.**

We apologise for lacking clarity in presenting the results. The purpose of this scoping review was to describe the literature available on this topic rather than the actual results presented in each study. The data presented in the results are a summary of the number of studies which report on factors associated with osteoporosis, falls and fractures, in each CIRD. Given the methodological differences, the actual reported prevalence or risks in individual studies are not presented as part of this scoping review. We have amended the wording in the results and titles of Tables 1-5.

9. **You can add a small comment about artificial intelligence use for the prediction os osteoporosis in those patient populations in the coming years given its importance in Medicine actually.**

We agree that artificial intelligence (AI) will have increasing utility in the healthcare setting, with AI enhanced tools in current development in various settings regarding risk stratification. We have added a comment about AI in our discussion (Discussion, Paragraph 4).

10. **Was it in sub-Saharan Africa or only in North Africa? please, specify throughout the manuscript and in case there was no study from sub-Saharan Africa, consider calling for studies from that part of the world.**

All studies published from Africa were from Egypt and Morrocco. We have amended the manuscript to reflect this and included the need for studies from Sub-Saharan Africa (Discussion, Paragraph 2).

11. **This data extraction form should be extended to better capture methodological information on included studies and study participants because this is a scoping review of epidemiological data. Furthermore, I think you could extend information on associated factors of osteoporosis. For instance, social determinants of osteoporosis have not been addressed in this review: see https://doi.org/10.1177/00469580231155719 dor example. Please, assess the associated factors of studied parameters as extensively as possible. These articles may be helpful:**

**-Leslie WD et al. Curr Osteoporos Rep**
**. 2020 Jun;18(3):115-129. doi: 10.1007/s11914-020-00590-7.**

**Vandenput et al. Osteoporosis International (2024) 35:469–494.**
**https://doi.org/10.1007/s00198-023-07012-1**
**Hong et al.**

We entirely agree with the importance of social determinants of osteoporosis. Demographic data collected included ethnicity, educational attainment, marital status and socioeconomic status as outlined in Tables 2-5. Thank you for the suggested references. We have included in our discussion the limitation in current literature in this topic in addressing social determinants of osteoporosis, falls and fractures (Discussion, Paragraph 2).

<u>Reply to Reviewer 2</u>
**Dr. Robert D Blank, Medical College of Wisconsin**
**Comments to the Author:**
Overall Considerations: This is a carefully conducted and clearly reported scoping review of observational studies reporting associations of 4 inflammatory rheumatic diseases with osteoporosis, fractures, and falls. Limitations of this review reflect limitations of the literature on the topic. I've offered a few suggestions for improving this useful work.

Major Points:
**1. It would be helpful if you were to assess the uniformity of the inclusion criteria for each of the 4 diseases considered. Does this literature suffer from inconsistent definition of the at-risk population?**

Thank you for your review and comment. The inclusion criteria for each of the rheumatic diseases included in this review was deliberately kept broad, to capture the general breadth of literature available in these conditions. However, there we acknowledge inconsistencies regarding the definition of rheumatic diseases utilised in these studies. This has now been described in the results and noted in the discussion. A new appendix (Appendix 4) has been included, summarising the definitions of each of the rheumatic conditions, and the outcomes of osteoporosis, falls and fractures, provided by each paper.

**2. Similarly, it would be helpful to address how outcomes were defined and assessed in the papers you reviewed. This is particularly important in gauging the uniformity of what was studied. With regard to fractures, please add information about sites studied/included/excluded. With regard to osteoporosis, what were diagnostic criteria and were these limited solely to BMD or were clinical criteria also included? With regard to falls, please include information about specific inclusion/exclusion criteria. Readers will want to know about methodological heterogeneity. You might consider (but need not) proposing criteria for defining outcomes.**

We included studies of osteoporosis, falls and osteoporotic fractures using all definitions (and fracture sites) to capture the breadth of literature available. The definition of outcomes is summarised in Appendix 4. The definitions used have been added to the results (Study Characteristics) and the challenge of methodological heterogeneity discussed in the Discussion, Paragraph 1.

**3. It would be helpful if tables 2-5 included bibliography numbers for the relevant papers.**

We agree with this comment. Our initial manuscript did not include these bibliography numbers due to the large number of total references in the main paper. We have made amendments and added bibliography references in additional tables available in Appendix 5 (the tables inclusive of bibliography numbers were too large for the main manuscript as per journal requirements and returned to us prior to re-submission).

**Minor Points:**
**1. Figure 1 (matrix) has minimal informative value, would either delete or add a scale to give symbol sizes meaning.**

This has been deleted, thank you.

**2. There is a recently published update to ref 1.**

This has been updated, thank you.

---

## VERSION 2 - REVIEW

---

| | |
|---|---|
| **Reviewer** | **1** |
| **Name** | **Essouma, Mickael** |
| **Affiliation** | **Faculty of Medicine and Biomedical Sciences, University of Yaoundé I, Yaoundé, Cameroon, Department of Internal Medicine and Specialties** |

**Date** **21-May-2025**

**COI**

---

Peer review report of manuscript bmjopen-2024-096226.R1 titled "The epidemiology and factors associated with osteoporosis, falls and fractures in patients with chronic inflammatory rheumatic disease: A scoping review"

1. General overview and recommendation

The authors have partly addressed my comments of the initially submitted version of the manuscript. However, I believe it is a service you owe to yourselves (given your ongoing primary study on osteoporosis, fractures and falls in people with CIRDs), to BMJ Open and to the CIRD patient and professional populations to thoroughly revise this rich review currently including 279 articles, and which will potentially include more studies once epistemic justice is made through the objective inclusion of data from Africa. Data from Africa are available here https://scholar.google.com/scholar?hl=en&as_sdt=0%2C5&q=autoimmune+diseases+AND+osteoporosis+AND+Africa&btnG=). Data from Africa are also available from the website of African Journal of Rheumatology, among other sources. I therefore propose a major revision of the current version of the article.

2. Specific comments

They are appended in the document bmjopen-2024-096266.R1_Proof_hi_Mickael Essouma comments.pdf attached to this review report.

Review report available online on 21/05/2025

By Mickael Essouma

**** **The reviewer provided a marked copy with additional comments. Please contact the publisher for full details.** ****

---

| | |
|---|---|
| **Reviewer** | **2** |
| **Name** | **Blank, Robert D** |
| **Affiliation** | **Medical College of Wisconsin** |
| **Date** | **01-Jun-2025** |
| **COI** | |

---

This MS is a scoping review of epidemiological studies exploring falls, fractures, and osteoporosis in 4 chronic inflammatory diseases: rheumatoid arthritis, systemic lupus erythematosis, ankylosing spondylitis, and psoriatic arthritis, spanning publications from

2000 through 2023. Methods are appropriate and clearly presented. Findings are fairly presented and interpretation is sound. Several notable gaps in the literature are identified. Notably, the vast majority of prior work addresses RA, with limited coverage of the AS and PsA. Authors identify important methodological limitations of the included papers, including heterogeneity of study populations and definitions of study outcomes. This is therefore a useful review that will help those planning new investigations in this space.

Specific points:

1. The statement at p 3 lines 11-13 is misleading IMO: While longer disease duration would increase exposure to risk of falls and fractures, DMARDs might also have a beneficial effect on the magnitude of those risks. These are competing factors whose net impact is uncertain.

2. Discussion of geographic coverage, p. 18, lines 10-13. You overlooked Latin America. Please add.

3. The WHO is presently considering the possibility of updating the definition of osteoporosis. This is a work in progress that may have an impact on future work. You may wish to alert readers to this.

---

## VERSION 2 - AUTHOR RESPONSE

Reviewer 1:

1. **The authors have partly addressed my comments of the initially submitted version of the manuscript. However, I believe it is a service you owe to yourselves (given your ongoing primary study on osteoporosis, fractures and falls in people with CIRDs), to BMJ Open and to the CIRD patient and professional populations to thoroughly revise this rich review currently including 279 articles, and which will potentially include more studies once epistemic justice is made through the objective inclusion of data from Africa. Data from Africa are also available from the website of African Journal of Rheumatology, among other sources. I therefore propose a major revision of the current version of the article.**

Thank you for your very valid comment. Our original search strategy searched three large electronic databases. After identification of paucity of identified studies from geographic areas including Africa, Oceania, Latin America and the Middle East and feedback, we have conducted an additional manual literature search to ensure the broad spectrum of relevant literature has been conducted. A manual literature search was conducted of search engine Google Scholar and with the search strategy added to Appendix 1 and results updated in the manuscript accordingly. Unfortunately, we were unable to conduct a search through the website of African Journal of Rheumatology due to the difficult nature of creating a focussed keyword search with date restrictions with the available search function. We identified an additional 9 articles, with Africa (6), Middle East (2) and South America (1). These additionally identified articles are as follows:

Aghaei M, Sedighi S, Behnampour N, Hezarkhani S, Shirashiani M, Mohebi EG, et al. Change in bone mineral density in post menopausal women with rheumatoid arthritis. Bangladesh Journal of Medical Science. 2013;12(2):158-63.

Fadda S, Hamdy A, Abulkhair E, Elsify HM, Mostafa A. Serum levels of osteoprotegerin and RANKL in patients with rheumatoid arthritis and their relation to bone mineral density and disease activity. The Egyptian Rheumatologist. 2015;37(1):1-6.

Franco CJV, Henao MPZ, Pineda FL, Montoya SP, Vallejo EO, Padilla LMR, et al. Low bone mass and osteoporosis in patients with systemic lupus erythematosus. Revista Colombiana de Reumatología (English Edition). 2017;24(1):4-10.

Hmamouchi I, Allali F, El Handaoui B, Amine H, Rostom S, Abouqal R, et al. The relation between disease activity, vitamin D levels and bone mineral density in men patients with ankylosing spondylitis. Rheumatology Reports. 2013;5(1):e3-e.

Ketabforoush AHME, Aleahmad M, Qorbani M, Mehrpoor G, Afrashteh S, Mardi S, et al. Bone mineral density status in patients with recent-onset rheumatoid arthritis. Journal of Diabetes & Metabolic Disorders. 2023;22(1):775-85.

Lala V, Tikly M, Musenge E, Govind N. Comorbidities in Black South Africans with established rheumatoid arthritis. International Journal of Rheumatic Diseases. 2022;25(6):699-704.

Mahdi MA, Erraoui M, Ngeuleu A, Tahiri L, Rkain H, Allali F. Falls, fear of falling and risk of falls in patients with rheumatoid arthritis: prevalence and associated factors. The Pan African Medical Journal. 2017;28(16).

Majjad A, Ghassem MA, Toufik H, Sadni S, Debbarh Z, Djossou JH, et al. Relationship between vertebral fracture prevalence and abdominal aortic calcification in women with rheumatoid arthritis. Bone. 2020;141:115599.

Wafa H, Raja A, Dhia K, Nada B, Imene Z, Montacer KM. Risk factors associated with bone loss and occurrence of fragility fractures in rheumatoid arthritis patients. The Egyptian Rheumatologist. 2019;41(1):1-5.

Amendments have been made to include these newly identified studies through Appendix 3, 4 and 5.

2. **Specific comments are appended in the document bmjopen-2024-096266.R1_Proof_hi_Mickael Essouma comments.pdf attached to this review report. We have addressed the major comments in the document as below:**

**It was difficult to imagine that this review would include as many studies as 279. So, highlighting the paucity of studies as a considerable limitation of this review is inconsistent with the number of included studies. Is there any aspect of the epidemiology of osteoporosis, fractures and falls in people with CIRDs that was studied in a relatively few number of studies? Specify.**

Thank you for this comment. This study highlights the contrast between the widely available literature in osteoporosis and osteoporotic fractures in CIRDs, in contrast to the limited number of studies on falls, rather than the overall paucity of literature. We have presented these results in Table 1. Of the 288 studies which report on outcomes of osteoporosis, falls and fractures, only 27 presented falls as an outcome measure. This discrepancy is particularly evident in non-RA CIRDs of PsA, AS and SLE.

Our comment on paucity of literature is a comment on the need for further research on the epidemiology and risk factors for falls in CIRDs. We have clarified this in the manuscript in the Strengths and Limitations section as below (Page 3, Line 25):

**STRENGTHS AND LIMITATIONS**

- ~~Comprehensive~~ Extensive literature review of studies available conducted on the epidemiology and factors associated with osteoporosis, falls and fractures in CIRD.
- Identification of a paucity of falls research in CIRD, compared to available literature in osteoporosis and osteoporotic fractures, highlighting the need for further research.
- Included publications from developed and/or resource-rich countries in the English language only is a limitation of the study.

3. **I do not think this is the most important limitation. There is likely two very important limitations to this review owing to limitations of included studies: 1) large heterogeneity od osteoporosis, fragility fractures and falls detection methods across studies. This impedes a proper comparison of osteoporosis, fractures and falls frequencies across studies. It also has the potential to decrease the reliability of osteoporosis, fragility fractures and falls frequencies reported in this review.**

Thank you. We have amended the manuscript to better acknowledge the limitations of conducting a review synthesising the frequencies of osteoporosis, osteoporotic fractures and falls in CIRD cohorts and relationships between associated factors, due to marked study heterogeneity. We have included comment regarding limitation in our Discussion (Page 17, Line 9 and Page 17, Line 22).

There is marked methodological heterogeneity, with differences in definitions for the outcome measures of osteoporosis, falls and osteoporotic fractures. Due to heterogeneity of study methodologies and case definitions, a comparison of the frequency of osteoporosis, osteoporotic fractures and falls in CIRDs is challenging and should be considered in the future design of observational studies and systematic reviews.

In addition, the heterogeneity of definitions of osteoporosis, fractures and falls; or the absence of a definition, leads to wide variability in reported frequencies and relevance of associated factors, highlighting the need to adopt a consensus definition for future observational studies to enable synthesis of findings.

**2) lack of thorough analysis of the relationship between the main review variables (osteoporosis, fragility fractures and falls) and the potential associated factors: were those relationship causal? linear? dose-response? Non-linear? Bidirectional relationships? Or a combination thereof? What type of associated factors were the ones recorded in this review: risk factors? Protective factors? A combination of both? Unspecified type of associated factors? It is so important to highlight these issues**

**because they help make sizeble recommendations that can substantially help advance future research on the epidemiology and mechanisms of osteoporosis in people with the studied CIRDs and ultimately lead to the development of more evidence-based and dignity-based recommendations for the management of osteoporosis, fractures and falls in people with CIRDs.**

Comparison of reported frequencies and relationship between osteoporosis, fractures and falls with associated factors in the included studies was not the aim of our study. Our review aimed to provide a comprehensive overview of the current literature available on the epidemiology and factors associated with osteoporosis, osteoporotic fractures and falls in CIRDs, including identifying limitations of currently available literature. The results focus on describing the characteristics of observational studies available, the number of studies reporting on each outcome and associated factors, to identify gaps in current literature, topics for further research and areas for improvement in future observational studies. We have made amendments to the Introduction to better clarify the aim of this scoping review (Page 4, Line 24).

> The purpose of this scoping review is to provide a comprehensive overview of the current literature available on the epidemiology and factors associated with osteoporosis, falls and fractures in RA, PsA, AS and SLE, to explore the methodologies utilised, identify gaps in current literature, topics for further research and areas for improvement in the design of future observational studies. This may lead to improved risk stratification of osteoporotic fractures in CIRD patients which may enable earlier diagnosis and intervention.

**While a 2021 pooled analysis of data on osteoporosis and osteopenia in people with psoriatic disease did not show an association between psoriatic disease and both osteopenia and osteoporosis, cautious should be exercised when stating that the association between axial spondyloarthritis (notably ankylosing spondylitis) and osteoporosis is debated because the most compelling published findings from the real world tend to link axial spondyloarthritis with low bone mass density: see Ramirez et al. Prevalence and risk factors for osteoporosis and fractures in axial spondyloarthritis: A systematic review and meta-analysis. Semin Arthritis Rheum . 2018 Aug;48(1):44-52. doi: 10.1016/j.semarthrit.2017.12.001. And Zuchowski et al. Osteoporosis in axial radiographic spondyloarthritis: diagnostic limitations of bone mineral density and the need for comprehensive fracture risk assessment. Rheumatology . 2024;62(6):466-474. doi: 10.5114/reum/194107. Epub 2024 Dec 24.**

Thank you. We agree with the currently available evidence that axial spondyloarthropathies have been shown to be associated with low bone density and higher fracture risk. In comparison to RA however, there does remain less certainty, due to the challenges of interpreting bone density using different diagnostic methods, fracture risk stratification (given spondyloarthropathy patients are usually younger, with a higher proportion of males compared to RA patients, bone formation as part of its pathophysiology) and marked bias in existing studies. We have made a clarification to this statement in the Introduction (Page 4, Line 9), inclusive of the suggested references.

> There is less certainty about the risk of osteoporosis, falls and fractures in spondyloarthropathies, including psoriatic arthritis (PsA) and ankylosing spondylitis (AS), with widely variable results in literature due to challenges with interpreting bone density data, the younger patient demographic and bias in existing studies [6, 7].

**Altogether, consider revising this statement with updated evidence and thoughts about the epidemiology of osteoporosis in patients with CIRDs. After this, the authors need to comment on the pathogenesis of osteoporosis in people with CIRDs, including the role of traditional risk factors of osteoporosis, CIRD-specific risk/driving factors of osteoporosis, and of the driving factors of osteoporosis related to disease-modifying drugs. While making these comments, the authors also need to consider the shared pathogenetic pathways between CIRDs and osteoporosis: see Wu et al. Front Endocrinol (Lausanne). 2023 Aug 29;14:1196269. doi: 10.3389/fendo.2023.1196269**

We have included a sentence to provide an overview of the proposed pathogenetic mechanisms through which osteoporosis is increased in CIRDs (Page 4, Line 4). Due to word count limitations, with the study focussed on an overview of available literature rather than on disease pathogenesis, we did not include an in-depth discussion on pathogenesis.

> Chronic inflammatory rheumatic diseases (CIRD), particularly rheumatoid arthritis (RA) and systemic lupus erythematosus (SLE) are associated with higher prevalence of osteoporosis, falls and fractures due to an interplay of complex mechanisms, including disease pathophysiology, treatments, joint deformities and impact on physical activity [2].

**This comment also needs to be tempered. Is reference 23 sufficient to state that FRAX has been validated to accurately detect osteoporosis in RA patients? Likewise, is reference 24 sufficient to conclude that FRAX has been validated to accurately detect osteoporosis in the SLE population?**

Thank you. There have been several large cohort studies through which FRAX score validity has been assessed. We have included additional references and changed the wording of the original sentence to avoid misconstruing currently available literature (Page 4, Line 18).

> The Fracture Risk Assessment Tool (FRAX), is a widely utilised method of fracture risk assessment inclusive of RA as a risk factor, with its validity studied in several large RA cohorts [9,

**Reference 25 is outdated to back up your comment on appropriate osteoporosis assessment tools in the axial spondylarthritis population: see Zuchowski et al. Osteoporosis in axial radiographic spondyloarthritis: diagnostic limitations of bone mineral density and the need for comprehensive fracture risk assessment. Reumatologia**
**. 2024;62(6):466-474. doi: 10.5114/reum/194107. Epub 2024 Dec 24.**

Reference 25 is a study which met inclusion criteria at the time of the conducted search. In the manuscript, we commented that this study utilised Trabebular Bone Scores (TBS) to define osteoporosis, rather than comment on its use on its appropriateness as an osteoporosis assessment tool. We therefore prefer to retain the use of this reference.

**Going through the published literature, I believe that a high-quality scoping review is needed to thoroughly describe the frequency and associated factors of osteoporosis, fractures and falls in people with the studied chronic inflammatory rheumatological diseases. However, this paragraph fails to convince me that such a review is actually necessary. You need to clearly specify in this paragraph the CIRDs assessed: RA, SLE, AS and PsA. Consider using these references to build a strong rationale for conducting this scoping review:**
**1. Cronin and George. The Why and How of the**
**Integrative Review. Organizational Research Methods.**
**https://doi.org/10.1177/1094428120935507.**
**2. Munn et al. BMC Medical Research Methodology (2018) 18:143**
**https://doi.org/10.1186/s12874-018-0611-x**

Thank you, we have utilised the above studies to better clarify our rationale for conducting this scoping review. We have made amendments to the third paragraph of the Introduction based on the above recommendations (Page 4, Line 24).

The purpose of this scoping review is to provide a comprehensive overview of the current literature available on the epidemiology and factors associated with osteoporosis, falls and fractures in RA, PsA, AS and SLE, to explore the methodologies utilised, identify gaps in current literature, topics for further research and areas for improvement in the design of future observational studies.

**The study selection process is usually a multiple-step process (transfer of records from the searched electronic databases to the software where study selection takes place, removal of duplicates, screening of titles and abstracts of articles potentially eligible, and final selection based on an assessment of the full-texts of articles included at the step of screening by title and abstract. These steps should be clearly described in the sequential order of execution.**

We have amended the study selection process as suggested in sequential order (Page 5, Line 17).

Records obtained from searched electronic databases were transferred into Covidence systematic review software. Duplicates were removed, with titles and abstracts were screened for relevance. A full-text review was performed by two authors (CC, GB) with exclusion of those which did not meet inclusion criteria. Articles obtained from the manual search, titles and abstracts were screened by author CC, with author GB verifying if inclusion criteria met.

**It is difficult for me to understand how this review helps advance the field of osteoporosis, fragility fractures and falls in people with systemic autoimmune and autoinflammatory diseases when going to the current results section. To improve the readability of the results section and also help advance the field, I suggest four main sub-sections in the results section:**

a. **Search results**
b. **Study characteristics**
c. **Frequency of osteoporosis, fragility fractures, and falls in people with CIRDs**
d. **Factors associated with osteoporosis, fragility fractures and falls in people with CIRDs**

**In each of the sub-sections "Frequency of osteoporosis, fragility fractures, and falls in people with CIRDs" and "Factors associated with osteoporosis, fragility fractures and falls in people**

**with CIRDs", the authors would then present data on each CIRD population (RA, SLE, AS and PsA) separately. The sub-section "Factors associated with osteoporosis, fragility fractures and falls in people with CIRDs" would be further subdivided into "driving /risk factors", "protective factors", and unspecified type of associated factor". You may summarize the associated factors in a figure. This article may help the authors build that figure and other figures that would enahnce the quality of this review:**
**Mozaffarian D. Dietary and Policy Priorities for Cardiovascular Disease, Diabetes, and Obesity – A Comprehensive Review. Circulation. 2016 January 12; 133(2): 187–225. doi:10.1161/CIRCULATIONAHA.115.018585.**

We agree a review which synthesises the frequency of osteoporosis, osteoporotic fractures and falls across each CIRD would be meaningful. Reporting and comparison of frequencies of these outcomes, or the associated factors, was beyond the scope of the present study. Rather we sought to describe the breadth of literature available (including number of studies, study characteristics), which may support design of further reviews to describe the frequencies of osteoporosis, osteoporotic fractures and falls, and its associated factors, as outcomes. We were able to highlight through our scoping review the potential challenges which may arise with comparison of frequencies based on study heterogeneity and case definitions, which is a consideration for other researchers who embark on designing a study aimed to address the frequency of osteoporosis, osteoporotic fractures and falls and the direction of associated factors.

Therefore, keeping in mind our aim of providing a comprehensive overview of the available literature of osteoporosis, osteoporotic fractures and falls in RA, PsA, AS and SLE, we prefer to maintain the structure of our Results section to report outcomes within each of these conditions, as we considered this approach provided clearer guidance to treating rheumatologists and researchers about the current state of literature of osteoporosis, osteoporotic fractures and falls within each disease category. We consider our current approach more clearly highlights the paucity of literature in PsA and AS, in comparison to RA and SLE; and in falls, compared to osteoporosis and fractures.

**I suggest the authors thoroughly discuss the definition of osteoporosis in included studies in a dedicated sub-section termed "Definitions of osteoporosis across included studies".**

We have included a paragraph in the Results section pertaining to definitions of osteoporosis, osteoporotic fractures and falls (Page 6, Line 18).

### Definitions of osteoporosis, osteoporotic fractures and falls

CIRD and outcome definitions utilised by included studies are summarised in Appendix 4. The WHO definition of osteoporosis, with BMD ≤-2.5 below the average value for young healthy women (T score of ≤-2.5) was the most frequently utilised definition. Several retrospective cohort studies utilised diagnostic codes, including International Classification of Diseases (ICD) and READ codes to identify cases of osteoporosis. Less frequently used definitions of osteoporosis included Trabecular Bone Scores (TBS) [13, 14] and lateral lumbar BMD values [15]. One study utilised the clinical definition of osteoporosis, of either osteoporosis based on BMD or history of osteoporotic fracture [16]. Twelve studies reported the definition of falls utilised, all following the Prevention of Falls Network Europe (ProFANE) definition of an unexpected event resulting in one to come to rest on the ground, floor or other level [17]. There was heterogeneity in the reported outcome of osteoporotic fractures, in the methods used to identify fractures and the fracture site. Methods ranged from unvalidated self-report to self-report validated by medical records and/or imaging, and vertebral based on imaging and ICD codes.

**I would delete this table (Table 1) to keep the table available in appendix 1. This table (Table 2, 3, 4, 5) could be moved to the appendix. What does this mean: the number of studies assessing osteoporosis in all the CIRDs assessed? What does this mean: mean age of study participants?**

Thank you. Table 1 provides a summary of the characteristics of studies which met our inclusion criteria. It highlights the discrepancy between available literature in falls, particularly in non-RA CIRDs, the geographic region of study publication (including the paucity of available literature from Australia/New Zealand, Middle East, Africa) and study methodology. We therefore prefer to retain Table 1.

Table 2-5 are also important in summarising the results relevant to the aim of our scoping review. Tables 2-5 summarise the number of studies which report on osteoporosis, osteoporotic fractures and falls in each of the included CIRDs (RA, PsA, AS and SLE) and the number of studies reporting on associated factors. The numbers in the table represent the number of studies identified, as outlined by the title of each table ("Number of studies reporting on the…) For example, Table 2 summarises how many studies report on osteoporosis, osteoporotic fractures and falls in patients with RA. There are 84 studies reporting on the frequency of osteoporosis in RA, compared to only 24 reporting on the frequency of falls in RA. The rows represent the number of studies reporting on associated factors with the outcome measure in RA. Out of the 84 studies reporting on the frequency of osteoporosis in RA, 51 studies report age as an associated factor.

**It is difficult for the current discussion to be a good interpretation of published data on osteoporosis, fragility fractures and falls in people with CIRDs given the methodological biases highlighted above and the current cumbersome presentation of review results. In any case, the discussion section would better summarize the main findings initially, subsequently discuss the main findings based on their sequential order of apparition in the results section, make a limitations and strengths statement, and finally make recommendations for future studies on osteoporosis, fragility fractures and falls, policies and clinical practice.**

We consider that the discussion reflects the aims of the scoping review, which is to provide a comprehensive overview of the available literature on osteoporosis, osteoporotic fractures and falls in CIRD, to identify gaps in literature, problems with methodologies and areas for improvement for future research, rather than a synthesis of the reported frequencies and directions of associations found in each included study. We found the gaps in literature are in falls research, particularly in non-RA CIRDs, geographic disparities of available research, marked variability in case definitions and few studies exploring DMARD use as associated factors. We have commented on recommendations for future studies, including need for large, prospective cohorts of patients inclusive of patients from low-middle income countries, inclusion of falls, with consistent definitions needed for osteoporosis, fractures and falls as outcome measures (Page 19, Line 25).

> Future research should utilise large, prospective cohorts of CIRD patients, inclusive of patients from low-middle income countries, inclusion of falls as an outcome with consistent definitions used for osteoporosis, osteoporotic fractures and falls. This may enable better understanding of the complex interaction between risk factors for osteoporosis, fractures and falls and enable development of disease-specific risk calculators which could guide preventive interventions.

**Where in this data extraction form did you record data on the frequency of osteoporosis, falls and fractures? I do not understand these variables, nor why you recorded so few variables in a scoping review addressing epidemiological data. The study variables recorded should be those that are expected to enable a proper assessment of the credibility and generalizability of findings in epidemiological studies: study period/duration, setting (community versus hospital), study's locality (urban versus semi-urban versus rural), data sources, sampling technique, method of sample size estimation, duration of follow-up in longitudinal studies, timeline of data collection (prospective versus retrospective versus ambispective), methods of case identification for the CIRDs and osteoporosis, falls and fractures, the types of frequencies estimated in included studies (incidence rate versus cumulative incidence/incidence proportion, point prevalence versus period prevalence), how were the frequencies reported (with 95% uncertainty intervals?), statistical methods used to assess the associated factors (logistic regression analysis? Others? Including the effect magnitude measures used to quantify the associations found), and the types of relationships found between osteoporosis, fractures and falls and their driving factors (linear and/or non-linear relationships? Causal relationships?), to name a few. (Regarding Appendix 2: Data extraction form)**

Reporting on the frequency of osteoporosis, falls and fractures in CIRD from included studies was not the aim of our scoping review, thus was not included on the Data extraction form.

<u>Reviewer 2</u>

1. **The statement at p 3 lines 11-13 is misleading IMO: While longer disease duration would increase exposure to risk of falls and fractures, DMARDs might also have a beneficial effect on the magnitude of those risks. These are competing factors whose net impact is uncertain.**

Thank you – we have made amendments to this statement to better clarify our intended message, that our patients with CIRD have improved disease control, leading to improved function and life-expectancy, enabling increased clinical focus on associated co-morbidities (Page 4, Line 14). We agree that DMARDs may have a beneficial impact on the magnitude of the risk of falls and fractures.

> With the advent of efficacious disease-modifying agents leading to improved function and life-expectancy, there has been an increased focus on addressing CIRD-associated co-morbidities, including osteoporosis, falls and related fractures.

The potential impact of DMARDs on risk of osteoporosis, osteoporotic fractures and falls is additional discussed in the Discussion (Page 18, Line 29).

> Despite the rapid uptake of advanced therapeutics in clinical practice, we found limited studies reported prospective data analysing the impact of advanced therapeutics on osteoporotic fractures and falls, translating to limited specific recommendations of osteoporosis management in CIRD [77].

2. **Discussion of geographic coverage, p. 18, lines 10-13. You overlooked Latin America. Please add.**

We agree with this statement - we have added Latin America (we used the term South America) to our discussion as an area with limited coverage of research in this area, thank you (Page 17, Line 5).

There is discrepancy in the geographic distribution of studies, with most studies published across Western Europe, East Asia and North America, with paucity of research from Oceania, Middle East, Sub-Saharan Africa and South America.

3. **The WHO is presently considering the possibility of updating the definition of osteoporosis. This is a work in progress that may have an impact on future work. You may wish to alert readers to this.**

Thank you for informing of the consideration of a change in the WHO definition. We agree this is of utmost importance across many aspects of clinical care, including research. Unfortunately, we were unable to find a supporting reference for this thus have not included this statement in the current manuscript. We will remain vigilant as any change in definition may have implications for our future research.
